# Associations of amyloid-β oligomers and plaques with neuropathology in the *App*^NL-G-F^ mouse

**DOI:** 10.1093/braincomms/fcae218

**Published:** 2024-06-25

**Authors:** Jiabin Tang, Helen Huang, Robert C J Muirhead, Yue Zhou, Junheng Li, John DeFelice, Maksym V Kopanitsa, Lutgarde Serneels, Karen Davey, Bension S Tilley, Steve Gentleman, Paul M Matthews

**Affiliations:** UK Dementia Research Institute, Uren Building, Imperial College London, White City Campus, London W12 0BZ, UK; Department of Brain Sciences, Burlington Danes Building, Imperial College London, Hammersmith Campus, London W12 0NN, UK; Department of Anesthesiology, Weill Cornell Medicine, Cornell University, New York, NY 11106, USA; Department of Metabolism, Digestion and Reproduction, Imperial College London, South Kensington Campus, London SW7 2AZ, UK; UK Dementia Research Institute, Uren Building, Imperial College London, White City Campus, London W12 0BZ, UK; Randall Centre for Cell and Molecular Biophysics, Kings College London, London SE5 9RX, UK; Department of Mechanical Engineering, Roberts Engineering Building, University College London, London WC1E 7JE, UK; UK Dementia Research Institute, Uren Building, Imperial College London, White City Campus, London W12 0BZ, UK; Department of Brain Sciences, Burlington Danes Building, Imperial College London, Hammersmith Campus, London W12 0NN, UK; UK Dementia Research Institute, Uren Building, Imperial College London, White City Campus, London W12 0BZ, UK; The Francis Crick Institute, London NW1 1AT, UK; Centre for Brain and Disease Research, Flanders Institute for Biotechnology (VIB), 9052 Gent, Belgium; UK Dementia Research Institute, Uren Building, Imperial College London, White City Campus, London W12 0BZ, UK; UK Dementia Research Institute, Kings College London, Denmark Hill Campus, London SE5 9RX, UK; Department of Brain Sciences, Burlington Danes Building, Imperial College London, Hammersmith Campus, London W12 0NN, UK; Department of Brain Sciences, Burlington Danes Building, Imperial College London, Hammersmith Campus, London W12 0NN, UK; UK Dementia Research Institute, Uren Building, Imperial College London, White City Campus, London W12 0BZ, UK; Department of Brain Sciences, Burlington Danes Building, Imperial College London, Hammersmith Campus, London W12 0NN, UK

**Keywords:** amyloid-β, oligomers, *App* ^NL-G-F^, imaging mass cytometry, neuropathology

## Abstract

Amyloid-β pathology and neurofibrillary tangles lead to glial activation and neurodegeneration in Alzheimer’s disease. In this study, we investigated the relationships between the levels of amyloid-β oligomers, amyloid-β plaques, glial activation and markers related to neurodegeneration in the *App*^NL-G-F^ triple mutation mouse line and in a knock-in line homozygous for the common human amyloid precursor protein (*App*^hu^ mouse). The relationships between neuropathological features were characterized with immunohistochemistry and imaging mass cytometry. Markers assessing human amyloid-β proteins, microglial and astrocytic activation and neuronal and synaptic densities were used in mice between 2.5 and 12 months of age. We found that amyloid-β oligomers were abundant in the brains of *App*^hu^ mice in the absence of classical amyloid-β plaques. These brains showed morphological changes consistent with astrocyte activation but no evidence of microglial activation or synaptic or neuronal pathology. In contrast, both high levels of amyloid-β oligomers and numerous plaques accumulated in *App*^NL-G-F^ mice in association with substantial astrocytic and microglial activation. The increase in amyloid-β oligomers over time was more strongly correlated with astrocytic than with microglia activation. Spatial analyses suggested that activated microglia were more closely associated with amyloid-β oligomers than with amyloid-β plaques in *App*^NL-G-F^ mice, which also showed age-dependent decreases in neuronal and synaptic density markers. A comparative study of the two models highlighted the dependence of glial and neuronal pathology on the nature and aggregation state of the amyloid-β peptide. Astrocyte activation and neuronal pathology appeared to be more strongly associated with amyloid-β oligomers than with amyloid-β plaques, although amyloid-β plaques were associated with microglia activation.

## Teaser

Tang *et al*. characterized the immunohistopathology of two human amyloid knock-in models. They found evidence for astrocyte activation with amyloid-β oligomers in *App*^hu^ mice expressing normal human peptide. In *App*^NL-G-F^ mice expressing disease-associated variant peptides, both high levels of amyloid-β oligomers and plaques existed with microglia and astrocyte activation and synaptic loss.

## Graphical Abstract

**Graphical Abstract:**
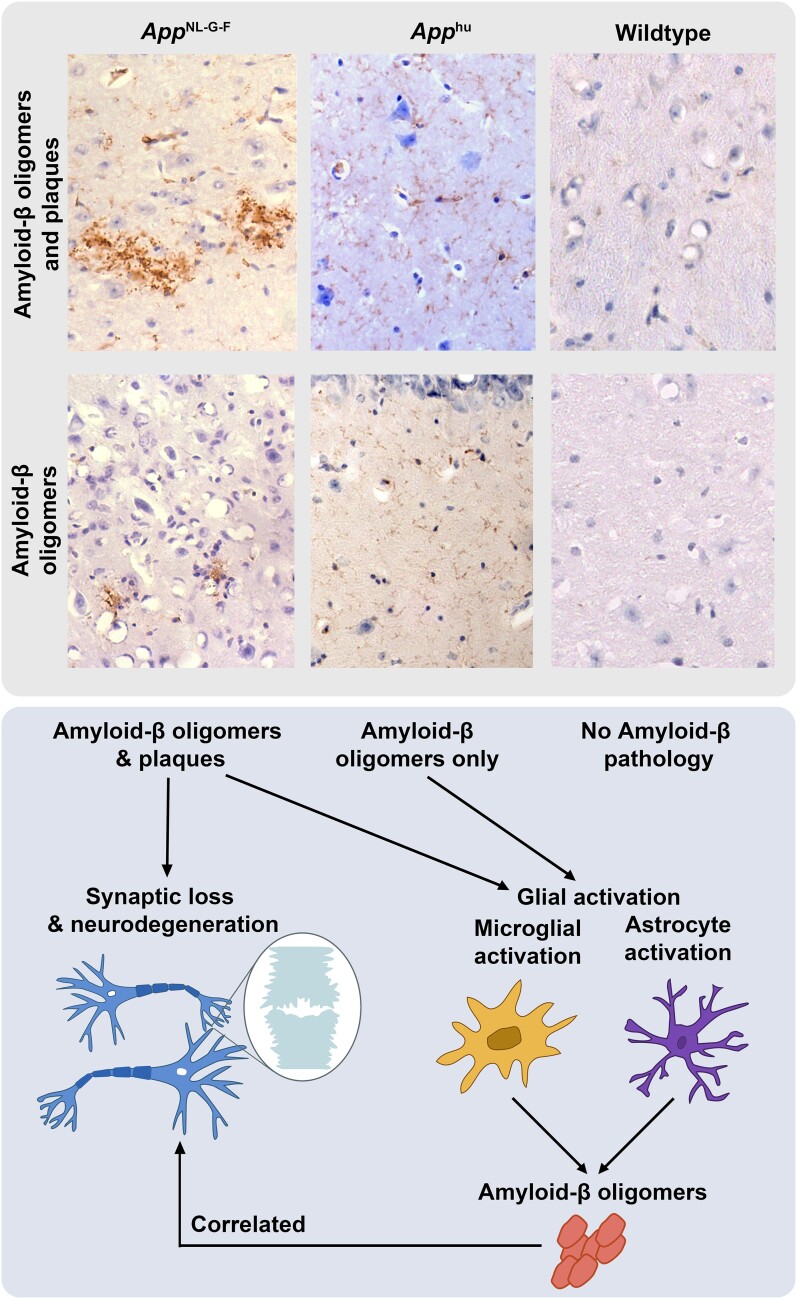

## Introduction

Amyloid-β (Aβ) pathology and tau neurofibrillary tangles, in association with glial activation and neurodegeneration, are hallmarks of Alzheimer’s disease.^1^ Microglia maintain tissue homeostasis, but when activated by Aβ or phosphorylated tau, can adopt a proinflammatory phenotype promoting neurodegeneration.^2^ Astrocyte activation can be associated with the release of cytokines, inflammatory factors and reactive oxygen species.^3-5^ Single-cell RNA sequencing has demonstrated consistent transcriptional changes in microglial and astrocytes that are associated with the presence of Aβ pathology.^6,7^

There are various forms of Aβ protein in the human brain, including monomers, dimers, oligomers and fibrils.^8^ Aβ fibrils tend to aggregate into plaques, which can be detected using *in vivo* PET imaging in patients with Alzheimer’s disease, while Aβ oligomers can be hardly detected.^9,10^ Different Aβ peptide conformations are associated with differential kinetics of aggregation or degradation.^11^ It has been assumed by many that neurotoxic species are associated primarily with Aβ plaques, but this has not been explored widely in pre-clinical models, in part because it has been difficult to distinguish between the consequences of increased levels of Aβ plaques and those of Aβ oligomers.^12,13^ The availability of knock-in mouse models expressing wild-type (WT) or mutated forms of human amyloid precursor protein (APP), under the control of an endogenous mouse promoter, may help to address this problem. Investigating the relationships between peptide sequence and clinically relevant neuropathological responses could help to identify epitopes for new therapeutic antibodies.^14^ Previous research suggested that Aβ oligomers accumulate around synapses.^15^ This could promote synaptic loss in Alzheimer’s disease. This highlights the need to investigate cellular crosstalk between Aβ proteins, glia and neurons.

In this study, we characterized brain cellular pathology in two mouse models. The *App*^NL-G-F^ is a knock-in mouse model that avoids transgenic artefacts caused by transfecting multiple copies of the *APP* gene.^16^ With humanization of the mouse *App* gene, the *App*^NL-G-F^ mouse includes three functionally relevant mutations: the Swedish mutation that promotes the total Aβ protein production, the Beyreuther/Iberian mutation that increases the Aβ_42_/Aβ_40_ ratio and the Arctic mutation that accelerates Aβ fibril assembly.^17,18^ We also characterized the brains of the *App*^hu^ mice, which express the human *APP* sequence under the mouse endogenous promoter.^19^ We studied the frontal cortex and hippocampus, two areas that are severely affected in a brain with Alzheimer’s disease. Cognitive dysfunction in Alzheimer’s disease is associated with dysconnectivity between the hippocampus and the frontal cortex;^20,21^ amnestic and spatial memory defects also depend largely on the connection of these two regions in the *App*^NL-G-F^ mouse.^22^

In this study, both immunohistochemistry (IHC) and imaging mass cytometry (IMC) have been used to image the histological expression of different Aβ forms and their spatial relationship with neuronal and glial cells displaying different functional phenotypes. We employed the NAB61 antibody, which targets potentially disease-relevant Aβ oligomers to complement the broader range of antibodies recognizing epitopes on higher-order aggregates and plaques.^23^

## Materials and methods

### Mouse tissue preparation

#### Ethical approval

Mouse brains were obtained under protocols approved by Animal Welfare and Ethical Review Bodies of the Medical Research Council Harwell Institute and Charles River UK Ltd.

#### Mouse breeding

WT and *App*^NL-G-F^ mice (or *App*^tm3.1Tcs^ mice, MGI: 5637817) were housed in a specific-pathogen-free condition (Mary Lyon Centre, MRC Harwell) and a specific-and-opportunistic-pathogen-free (Charles River UK) condition adhering to environmental conditions as outlined in the UK Home Office Code of Practice. *App*^hu^ mice (or *App*^em1Bdes^ mice, MGI: 6512851) were housed in a specific-pathogen-free condition in KU Leuven (Belgium). All animals were of the C57Bl/6J background. All animals had *ad libitum* access to water and standard rodent food and were kept on a 12-h light and dark cycle. All animal experiments were performed in accordance with UK Home Office Project Licenses for breeding genetically altered animals at the Medical Research Council Harwell Institute and Charles River UK Ltd.

#### Sample size

Sample size was estimated based on previous experiments performed in the laboratory.^24^ All experiments with *n* = 6 were carried out with 3 male and 3 female mice. A sex comparison was conducted with pathology, and no consistent difference was found (Supplementary Fig. 1). The following experiments with *n* = 3 were carried out with male mice only. All experiments were randomized to avoid sex, litter and batch effects. Investigators were blinded when performing all experiments. For staining with SV2A and PSD95, all samples were prepared at the same time to reduce optical density batch effects.

#### Tissue preparation

The mice were euthanized by sodium pentobarbital injection, exsanguinated, and their brains were quickly dissected on ice. Then, a transverse cut along the midline was used to separate the hemispheres. Right hemispheres were used for this study and were post-fixed in 4% paraformaldehyde for 24 h at 4°C. Then, after a brief wash with phosphate-buffered saline (PBS), they were cryo-protected in 30% sucrose in PBS for 2–3 days until they had sunk and kept in the same solution at 4°C. Finally, the brains were immersed into an optimum cutting temperature compound (Tissue-Tek 4583) and snap-frozen on dry ice. The cryostat (Leica, CM1900) was used to section the tissue at 10 μm, and the slides were stored at −80°C until further use.

### Genotype confirmation

#### DNA extraction and polymerase chain reaction

The DNA was extracted with DNeasy Blood & Tissue Kits (Qiagen 69504), according to the manufacturer’s instructions. DNA concentration was then tested with a nanodrop spectrophotometer.

The polymerase chain reaction (PCR) was carried out with the Q5 High-Fidelity 2X Master Mix (New England Biolabs M0492S), and the manufacturer’s setup protocol for a 25-µL reaction was followed (Supplementary Tables 1 and 2). Three primers were used according to Saito *et al*.,^16^ namely, 5′-ATCTCGGAAGTGAAGATG-3′ (WT primer), 5′-ATCTCGGAAGTGAATCTA-3′ (*App*^NL-G-F^ primer) and 5′-TGTAGATGAGAACTTAAC-3 (common primer). For the bioanalyzer step, the template DNA was diluted to a concentration of 10–20 ng/µl. For the electrophoresis step, 5 µl template DNA was added. The assembly of all reaction components was carried out quickly at room temperature (RT), and the PCR tubes were transferred to a thermal cycler (Bio-Rad, C1000).

#### Bioanalyzer

The Select-a-Size DNA Clean & Concentrator Kit (Zymo Research, D4080) was used, according to the manufacturer’s instructions. For a 636-bp DNA sample, 25 µl of the DNA solution was added to 125 µl of the Select-a-Size DNA Binding Buffer. After DNA elution, the High-Sensitivity DNA Kit (Agilent, 5067-4626) was used, and the DNA high-sensitivity bioanalyzer chips were run in accordance with the manufacturer’s guidelines. The chips were put into an Agilent 2100 Bioanalyzer after vortexing on an IKA vortex mixer for 1 min at 700 × *g*. The instrument’s software was used, and the programme for dsDNA was chosen. After ∼45 min run, the data were exported.

#### Electrophoresis

To prepare a 1.5% gel, 1.5 g agarose was added to 100 ml of Tris-borate-EDTA. Then, 5 µl of Gel Loading Dye (Biolabs, B7024S) was added to each 25 µl DNA sample, and Quick-Load 100 bp DNA ladder (Biolabs, N0551G) was added to the ladder well. Next, the gel was run at 120 V for 20–30 min. Finally, the gel was placed into a UVP BioDoc-It Imaging System.

### IHC staining

The IHC 3,3′-diaminobenzidine (DAB) staining was carried out with two different kits, Supersensitive Polymer HRP Kit (BioGenex) or ImmPRESS Polymer Detection Kit (Vector). Three sections separated by ∼300 µm were selected in the hippocampus and frontal cortex separately in each mouse to represent the whole region. The on-slide sections were air dried for at least 1 h, and put into three changes of PBS (5 min each). Then, the sections were incubated for 30 min in PBS containing 0.3% H_2_O_2_. Next, the sections were subsequently rinsed in distilled water (5 min) and PBS (3 × 5 min) before further procedures. The primary antibodies were diluted with PBS-containing 0.3% Triton (PBST), and the sections were incubated with primary antibodies overnight at 4°C. The primary antibody selection and dilution, as well as the incubation time of DAB are shown in Supplementary Table 3. No antigen retrieval step was used.

#### Supersensitive Polymer HRP Kit (SS kit)

Following incubation with primary antibodies in a humid chamber, the sections were incubated with Super Enhancer Reagent for 20 min, and Polymer HRP for 30 min. Sections were washed twice for 5 min with PBS between each step, and the sections were visualized with DAB at RT after three 5-min PBS washes. Subsequently, the sections were washed with distilled water (2 × 5 min), and incubated in haematoxylin (Mayer, MHS32-1L) for about ∼1 min before being rinsing with tap water for 5 min. Finally, after dehydration (70, 90, 100 and 100% industrial methylated spirit; 3 min for each step) and clearing steps (three changes of 100% xylene, 5 min each), the sections were coverslipped with dibutylphthalate polystyrene xylene mountant.

#### ImmPRESS Polymer Detection Kit (Ip Kit)

Sections were treated with horse or goat serum (according to the host species of secondary antibodies) for 20 min prior to immediate incubation with primary antibodies in a humid chamber. Following this, the tissue was washed in PBS, and appropriate secondary antibodies were applied for 30 min. Subsequently, the tissue was washed in PBS (3 × 5 min) and visualized with DAB at RT. Finally, the haematoxylin, dehydration and clearing steps were performed as above.

### Immunofluorescence staining

Sections were air dried for at least 1 h and washed with PBS (3 × 5 min each). Following antigen retrieval (Supplementary Table 4), the sections were incubated at 4°C overnight or RT for 2.5 h in a solution with a mixture of primary antibodies after subsequent wash in distilled water (5 min) and PBS (3 × 5 min). The list of primary antibodies, dilutions and incubation times are shown in Supplementary Table 4. The incubation conditions were optimized for the best signal-to-noise ratio in each case. Next, sections were washed twice in PBS and incubated with appropriate secondary antibodies (Supplementary Table 5) for 60 min. After washing with PBS (3 × 5 min each), 0.4% Sudan Black (Thermo Fisher Scientific, 4197-25-5) in 70% industrial methylated spirit was applied for 10 min to reduce the autofluorescence. Finally, the sections were rinsed with distilled water for 15 min and mounted with Antifade Mounting Media containing 4′,6-diamidino-2-phenylindole (Vector, H-1200).

### Imaging mass cytometry

IMC is an advanced technology, combining a novel laser ablation system with mass cytometry that allows visualization of the simultaneous expression of up to 40 markers in the same section, providing a powerful tool to study spatial relationships between proteins.^25^

#### Antibody conjugation with metal

The process of antibody conjugation with metal was carried out with Maxpar X8 Antibody Labeling Kits (Fluidigm, 201300), according to the User Guides. All of the solution cocktails were mixed thoroughly before centrifugation or incubation.

Ninety-five microlitres of L buffer were added to the X8 polymer tube for resuspension, and 5 μl of Ln metal solution was added before incubation at 37°C for 40 min in a water bath. Next, the mixture was added to a 3-kDa filter unit, with another 200 μl of L buffer added before centrifugation at 12 000 *× g* for 25 min at RT. Then, 400 μl of C buffer was added before another centrifugation at 12 000 *× g* for 30 min at RT.

Next, 100 μg of the antibody was loaded onto a 50-kDa filter, and the total volume was adjusted to 400 μl with R buffer before centrifugation at 12 000 *× g* for 10 min at RT. Next, 100 μl of a freshly prepared 4 mM tris(2-carboxyethyl)phosphine hydrochloride solution (MilliporeSigma, 646547) was added before incubation at 37°C for 30 min in a water bath for antibody reduction. Then, 300 μl of C buffer was added immediately after the incubation before centrifugation at 12 000 *× g* for 10 min at RT, and another 400 μl of C buffer was added with a repeated centrifugation step.

The purified Ln-loaded polymer and purified partially reduced antibody were retrieved separately, and the Ln-loaded polymer was resuspended with 60 μl of C buffer before mixing with a corresponding partially reduced antibody. The mixture was then incubated at 37°C for 90 min in a water bath for conjugation. Next, 200 μl of W buffer was used to wash the conjugation mixture with centrifugation at 12 000 *× g* for 10 min, with three washes with 400 μl of W buffer, each followed by centrifugation. After the final wash with W buffer, 80 μl of W buffer was added to dilute the conjugate for protein quantification, and another centrifugation at 12 000 *× g* for 10 min was carried out to remove W buffer. The 50 kDa columns were used for the centrifugation steps. Finally, Antibody Stabilizer PBS (Boca Scientific, 131 050) with 0.05% sodium azide (MilliporeSigma, 71289) was added to the conjugated antibody to obtain a final 0.5 mg/ml solution.

Protein quantification was carried out using the Qubit Protein Assay (Thermo Fisher Scientific, Q33212). Protein buffer and dye were mixed at a ratio of 200:1. Three Qubit Protein Standards (10 μl of each) were added to 190 μl of the mixture separately for calibration in sequence, and 2 μl of the sample was added to 198 μl of the mixture for quantification. Afterwards, the quantification was carried out with a Qubit 4 Fluorometer (Thermo Fisher Scientific, Q33226).

#### Staining and metal detection

Three sections separated by ∼300 µm between each other were selected in the hippocampus and frontal cortex in each mouse to represent the whole region. The tissue sections were air dried for at least 2 h, and washed with PBS (3 × 5 min). Then, followed by heating with EDTA (pH = 8) at 96°C for 20 min, the sections were incubated in a primary antibody cocktail (Supplementary Table 6) overnight at 4°C. The primary antibodies were diluted with 0.5% bovine serum albumin in PBST. After washing with PBS (2 × 8 min), Intercalator-Ir (Fluidigm, 201192A, 1:400) was applied for 30 min at RT for nuclei staining. Finally, the sections were washed with distilled water (2 × 5 min), and air dried for at least 2 h before IMC ablation and metal detection with Helios System connected to Hyperion Imaging System (Fluidigm). Image processing was performed with MCD Viewer, and images were exported in Tiff format.

#### Pixel classification and single-cell segmentation

All of the image channels were merged with ImageJ (Fiji, version 2.1.0), and put into Grayscale with the extended macros provided by Stephen Rothery in Imperial FILM Facilities. Only the Ir channel, indicating nuclear labelling, was left blue. Then, the composite images were saved in Jpeg format and processed with Ilastik 1.3 (University of Heidelberg, Germany) for pixel classification. The Ilastik was trained manually by selecting pixels of interest to identify and differentiate signal, nuclei or background. We used ∼2500 contiguous pixels (50 × 50 pixels matrix) for training on each brain section, applying the following feature selection parameters: colour/intensity, 10; edge, 10; texture, 10. Finally, probability maps were created and exported in the Tiff format. CellProfiler 4.2.1 (Broad Institute, USA) was then used to process the probability maps. Seven modules were added, including Color To Gray, Identify Primary Objects, Identify Secondary Objects, Identify Tertiary Objects, Mask Objects, Convert Objects To Image and Save Images. The images were then analysed automatically to create a mask for single-cell segmentation.

### Microscopy

IHC representative images were captured by a light microscope (Vanox, AHBT3) using a 20× objective. IHC images for quantitative analysis were captured with Digital Pathology Slide Scanners (Leica, Aperio AT2) using a 20× objective. Immunofluorescence (IF) images were captured by a Zeiss Axio Observer Inverted Microscope (Carl Zeiss Limited) in the FILM Facility of Imperial College London with a 20× objective, which was controlled by Zen acquisition software.

### Statistical analysis

#### IHC analysis

The cell counting, process length, process area and soma area were done using Halo v2.1 software. The analysis plan was set before the experiments. Process length and process area were measured within 10 μm around the cell soma. The data analysis was performed with one- or two-way ANOVA (illustrated in figures) using GraphPad Prism 8.4 software. The Tukey test as implemented in GraphPad Prism 8.4 software was used. The data were tested for normality with a Shapiro–Wilk test.

#### Phenograph and correlation plots

HistoCAT 1.73 (University of Zurich, Switzerland) was used to run t-SNEs, phenographs, heat maps and correlation plots. The cell mask was saved in the same folder with all correlated Tif images exported from ImageJ. The whole folders were then loaded to histoCAT, and t-SNEs as well as phenographs were run to differentiate the cells into different clusters. Then, heat maps were created to show how the clusters were defined, and correlation plots were used to show the spatial relationships between two specific cell markers. Pearson correlation analysis was then carried out. Correlation was defined by 4-pixel expansion. Data were tested for normality with a Shapiro–Wilk test.

#### Sholl analysis

IMC images were processed with ImageJ 2.1.0 (National Institute of Health, USA). The regions of interest (ROIs) of Aβ plaques or oligomers were manually selected, and added to ROI manager. A code was then run to enlarge ROIs with the same distance (Supplementary File). The number of required rings was set to be 3, and the thickness of rings was set to be 30 μm. The area coverage % in each ring was then measured. The data analysis was processed with GraphPad Prism 8.4 software.

## Results

### Differences in the abundance of Aβ oligomers and plaques in the *App*^NL-G-F^ and *App*^hu^ mice

**Figure 1:**
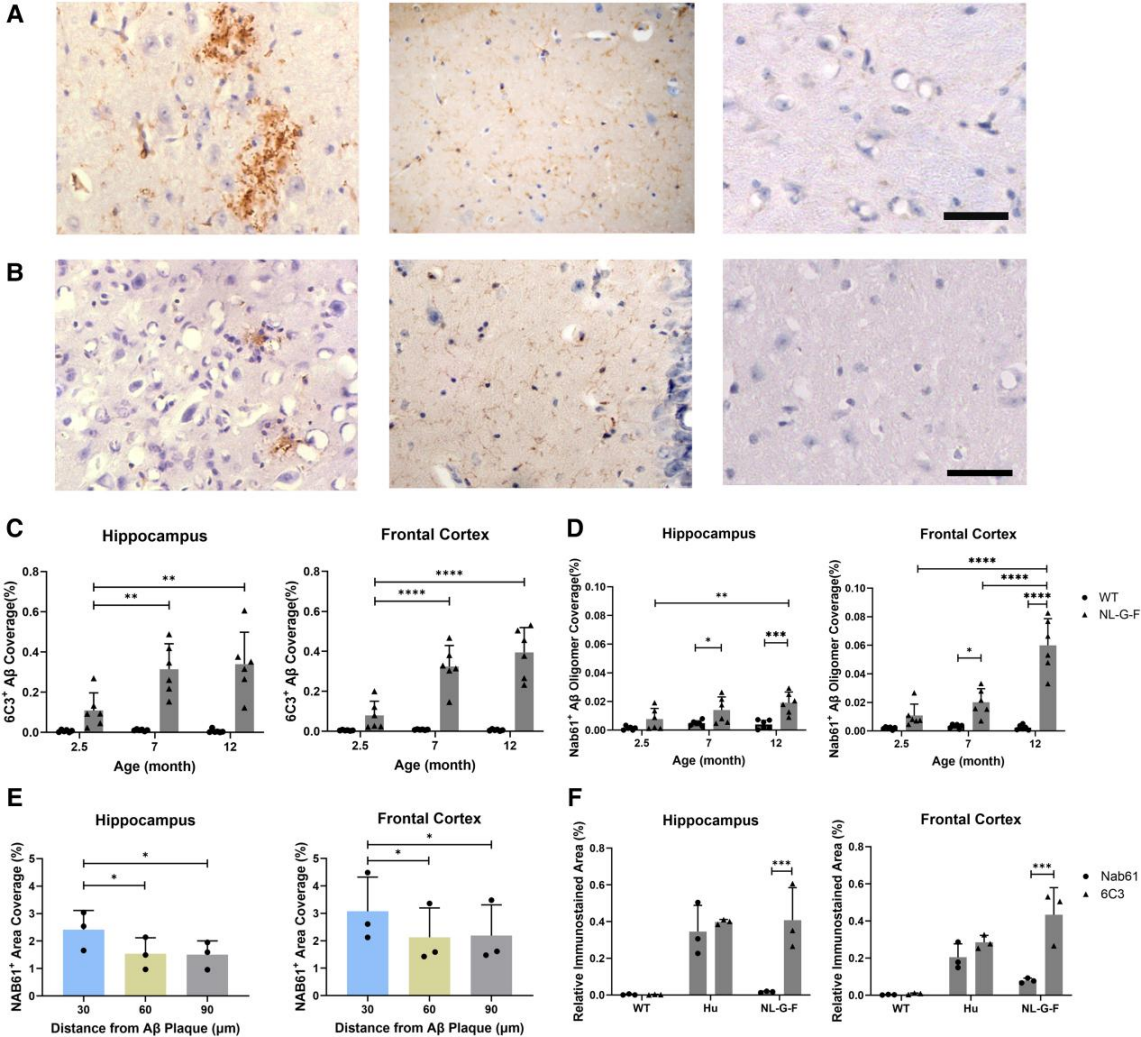
**A comparison of Aβ pathology in the HIP and FC of *App*^NL-G-F^, *App*^hu^ and WT mice.** (**A**) Representative images of 6C3^+^ Aβ plaques and oligomers in 12-month-old mice. Scale bar = 50 μm. (**B**) Representative images of NAB61^+^ Aβ oligomers in 12-month-old mice. Scale bar = 50 μm. (**C**) Relative areas (%) occupied by 6C3^+^ Aβ plaque of *App*^NL-G-F^ and WT mice (*n* = 6). HIP: *F*(2, 30) = 5.817, *P* = 0.0073. FC: *F*(2, 30) = 15.3, *P* < 0.0001. (**D**) Relative areas (%) occupied by NAB61^+^ Aβ oligomer of *App*^NL-G-F^ and WT mice (*n* = 6). HIP: *F*(2, 30) = 4.378, *P* = 0.0215. FC: *F*(2, 30) = 16.54, *P* < 0.0001. (**E**) A Sholl analysis using IMC of 6C3^+^ and NAB61^+^ Aβ staining in *App*^NL-G-F^ mice at 12 months (*n* = 3, one-way ANOVA). HIP: *F*(1, 2.001) = 47.43, *P* = 0.0204. FC: *F*(1.001, 2.001) = 160.9, *P* = 0.0061. (**F**) A comparison between IHC signals for Aβ plaques and Aβ oligomers in 12-month-old mice (*n* = 3). HIP: *F*(2, 12) = 23.85, *P* < 0.0001. FC: *F*(2, 12) = 25.82, *P* < 0.0001. Columns represent the mean ± standard deviation (SD). Statistical analysis was performed using two-way ANOVA unless specifically labelled. WT, wild type; HIP, hippocampus; FC, frontal cortex. Each data point represents the mean of three technical replicates in one mouse. *P < 0.05; **P < 0.01; ***P < 0.001; ****P < 0.0001.

We first assessed Aβ plaque and Aβ oligomer staining in the hippocampus and frontal cortex of 2.5-, 7- and 12-month-old mice (*n* = 6 for both *App*^NL-G-F^ and WT mice at each age). Aβ plaques with variably dense appearances (Fig. 1A) increased in the *App*^NL-G-F^ mouse by 2- to 3-fold between 2.5 and 7 months, without further significant change at 12 months (Fig. 1C). The Aβ oligomer staining (Fig. 1B) increased progressively between 2.5 and 12 months (Fig. 1D). NAB61^+^ Aβ oligomers were localized in or immediately around plaques (Fig. 1E and Supplementary Table 7).

Highly dense, process-like oligomeric Aβ staining was observed in 12-month-old *App*^hu^ mice (Fig. 1A and B). There was a significant difference between 6C3^+^ and NAB61^+^ areas in *App*^NL-G-F^ mice (Fig. 1F), indicating predominant staining of Aβ plaques. However, in *App*^hu^ mice, the areas stained for Aβ oligomers and plaques were similar (Fig. 1F), with process-like staining mostly attributable to Aβ oligomers. No specific Aβ staining was seen with either antibody in the hippocampus or frontal cortex of WT mice.

### Neuronal and synaptic loss were independent of Aβ plaque load in *App*^NL-G-F^ mice

**Figure 2:**
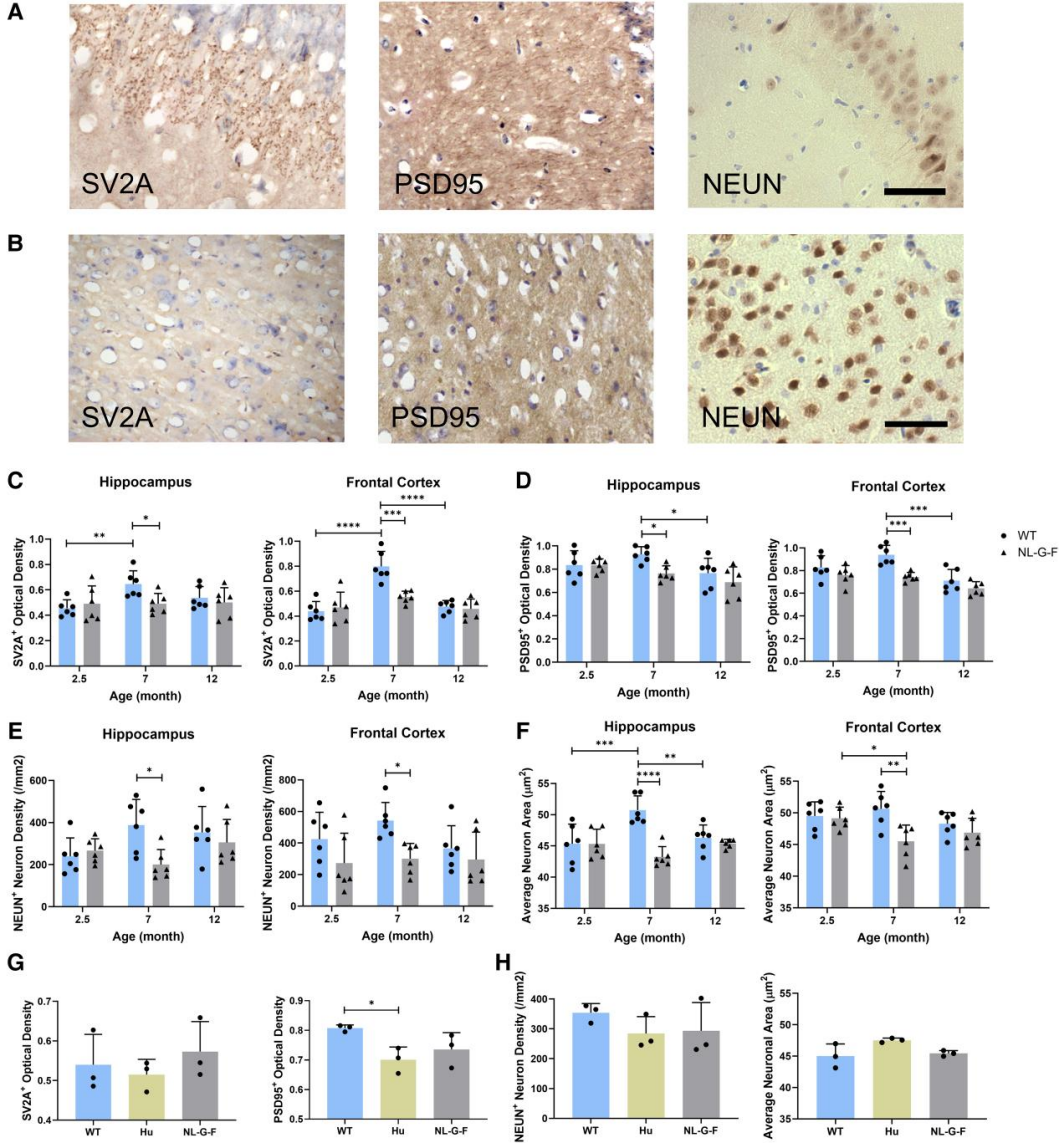
**A comparison of neuronal and synaptic changes in the HIP and FC of *App*^NL-G-F^, *App*^hu^ and WT mice.** (**A**) Representative images of IHC staining for SV2A^+^ pre-synapses, PSD95^+^ post-synapses proteins and NEUN^+^ neurons in HIP. (**B**) Representative images of IHC staining for SV2A^+^ pre-synapses, PSD95^+^ post-synapses proteins and NEUN^+^ neurons in FC. (**C**) Optical density of SV2A^+^ pre-synapses (*n* = 6). HIP: *F*(2, 30) = 2.629, *P* = 0.0887. FC: *F*(2, 30) = 8.456, *P* = 0.0012. (**D**) Optical density of PSD95^+^ post-synapses (*n* = 6). HIP: *F*(2, 30) = 5.08, *P* = 0.0126. FC: *F*(2, 30) = 13.06, *P* < 0.0001. (**E**) NEUN^+^ neuronal density. HIP: (**F**) NEUN^+^ average neuronal area (*n* = 6). HIP: *F*(1, 30) = 4.521, *P* = 0.0418. FC: *F*(1, 30) = 9.349, *P* = 0.0047. (**G**) Optical density of pre-synaptic (SV2A^+^) and post-synaptic (PSD95^+^) signals in the HIP at 12 months (*n* = 3, one-way ANOVA). SV2A: *F*(2, 6) = 0.5834, *P* = 0.5868. PSD95: *F*(2, 6) = 5.141, *P* = 0.05. (**H**) NEUN^+^ neuronal density and average neuronal area in the HIP at 12 months (*n* = 3, one-way ANOVA). Density: *F*(2, 6) = 0.9782, *P* = 0.4289. Area: *F*(2, 6) = 4.061, *P* = 0.0767. Columns represent the mean ± SD. Statistical analysis was performed using two-way ANOVA unless specifically labelled. Density is calculated as cell count/area. Scale bar = 50 μm. SV2A, synaptic vesicle glycoprotein 2A; PSD95, post-synaptic density protein 95; NEUN, neuronal nuclear protein; WT, wild type; HIP, hippocampus; FC, frontal cortex. Each data point represents the mean of three technical replicates in one mouse. *P < 0.05; **P < 0.01; ***P < 0.001; ****P < 0.0001.

We assessed synaptic and neuronal staining in the hippocampus and frontal cortex of 2.5-, 7- and 12-month-old mice (Fig. 2A and B). At 7 months, WT mice had significantly higher SV2A^+^ and PSD95^+^ staining than *App*^NL-G-F^ mice (Fig. 2C and D). At the same time point, the average neuronal soma areas and NEUN^+^ optical density were significantly lower in *App*^NL-G-F^ mice, suggesting neuronal dystrophy (Fig. 2E and F).^26^ No substantial differences were seen in synaptic or neuronal staining in the samples from *App*^NL-G-F^ and WT mice at 12 months. We also assessed these measures in the *App*^hu^ mice, in which we found an ∼13% decrease in PSD95^+^ staining optical density in the hippocampus of *App*^hu^ mice compared with that in WT mice (Fig. 2G and H and Supplementary Fig. 2A and B). This was not accompanied by a significant change in SV2A staining optical density.

### Spatial proximity Sholl analysis of NAB61^+^ Aβ oligomers and neuronal markers in *App*^NL-G-F^ mice

**Figure 3:**
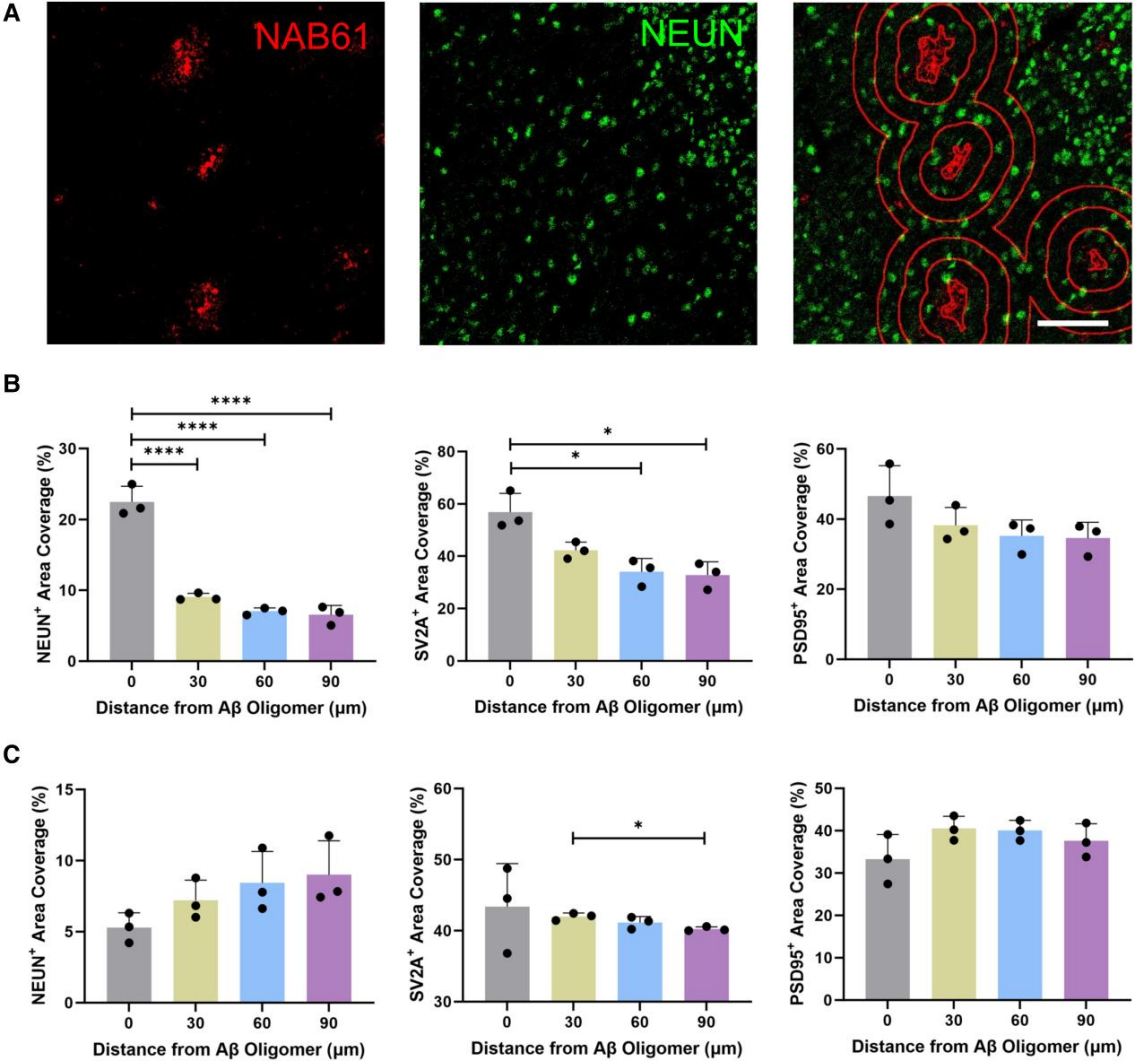
**A Sholl analysis of Aβ oligomers and neuronal markers in the hippocampus of *App*^NL-G-F^ mice (*n* = 3).** (**A**) Representative IMC images with a Sholl analysis of NAB61^+^ Aβ oligomers (red) and NEUN/PSD95/SV2A (green) in a 12-month-old mouse. (**B**) A Sholl analysis of NAB61^+^ Aβ oligomers and NEUN/PSD95/SV2A in 2.5-month-old mice. NEUN: *F*(3, 8) = 97.07, *P* < 0.0001. SV2A: *F*(3, 6.816) = 12.24, *P* = 0.0039. PSD95: *F*(1.002, 2.005) = 5.511, *P* = 0.1432. (**C**) A Sholl analysis of NAB61^+^ Aβ oligomers and NEUN/PSD95/SV2A in 12-month-old mice. NEUN: *F*(1.312, 2.625) = 11.06, *P* = 0.0538. SV2A: *F*(1, 2) = 0.5427, *P* = 0.538. PSD95: *F*(1.022, 2.044) = 13.77, *P* = 0.0635. The columns represent the mean ± SD. Statistical analysis was performed using one-way ANOVA. Ring distance = 30 μm. Scale bar = 100 μm. NEUN, neuronal nuclear protein; SV2A, synaptic vesicle glycoprotein 2A; PSD95, post-synaptic density protein 95. Each data point represents the mean of three technical replicates in one mouse. *P < 0.05; ****P < 0.0001.

IMC and Sholl analyses were used to explore the spatial relationships between Aβ pathology and neuronal or synaptic markers in 2.5- and 12-month-old *App*^NL-G-F^ mice (*n* = 3, male) in the hippocampus and frontal cortex (Fig. 3A and Supplementary Fig. 2C). At 2.5 months, mice showed a high degree of proximity of neuronal and synaptic marker staining signals to NAB61^+^ Aβ oligomers (Fig. 3B and Supplementary Fig. 3A, E and I). 6C3^+^ Aβ staining was also more abundant near SV2A^+^ synapses, but this trend was not found with NEUN^+^ neurons or PSD95^+^ synapses (Supplementary Fig. 3B, F and J). However, by 12 months, there was lower proximity of neuronal and synaptic markers with NAB61^+^ Aβ staining (Fig. 3C and Supplementary Fig. 3C, G and K). There was also a trend for lower co-localization of SV2A^+^ and PSD95^+^ synapses with 6C3^+^ Aβ staining, potentially as a consequence of local synaptic loss and neuronal dystrophy (Supplementary Fig. 3D, H and L).

### Differences in associations of Aβ with glial activation in *App*^NL-G-F^ and *App*^hu^ mice

**Figure 4:**
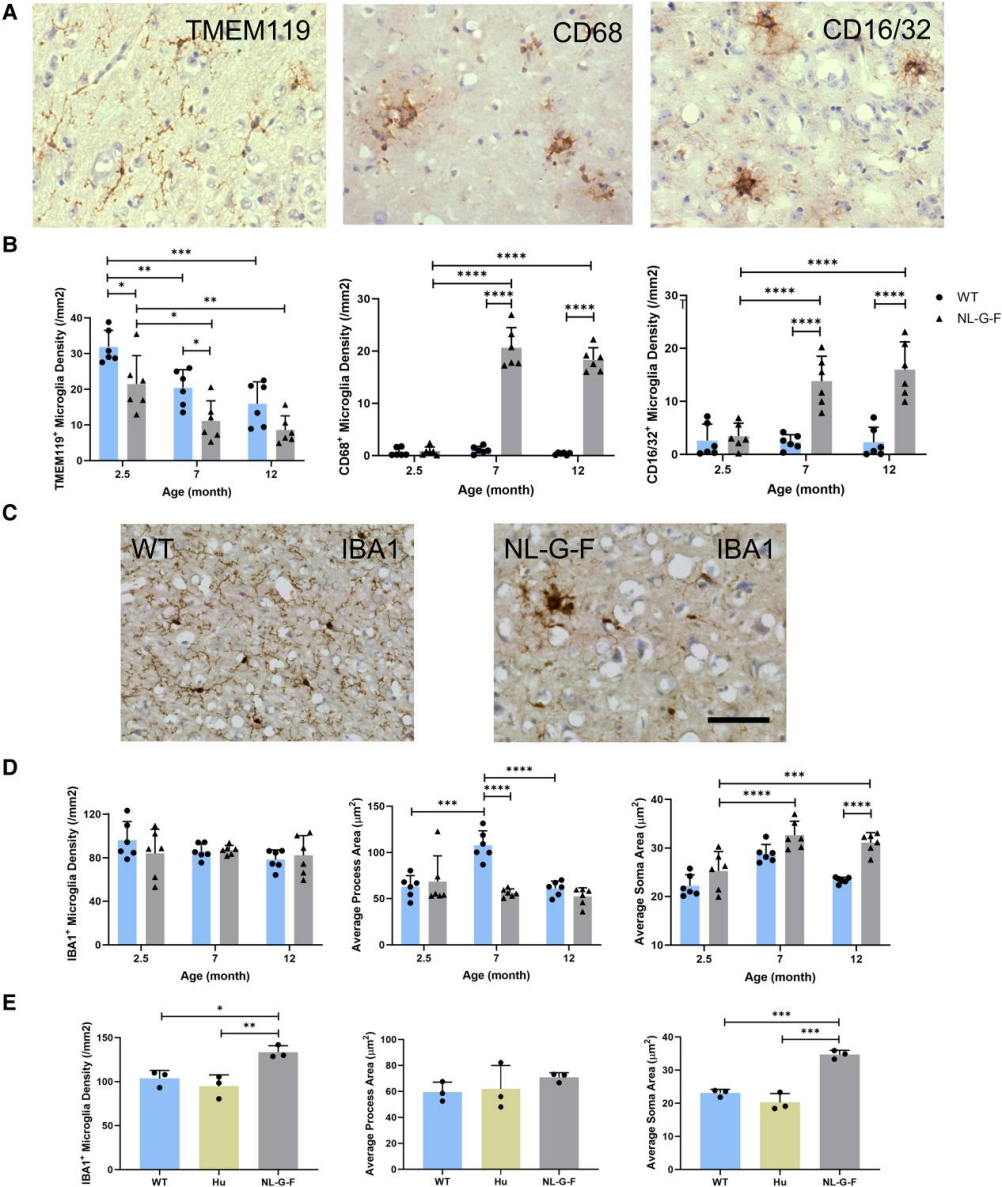
**Age-related changes of microglia density and morphology in the HIP and FC of *App*^NL-G-F^, *App*^hu^ and WT mice.** (**A**) Representative images of IHC staining for TMEM119^+^ inactive microglia, CD68^+^-activated microglia and CD16/32^+^ proinflammatory microglia. (**B**) Microglia density of TMEM119^+^, CD68^+^ and CD16/32^+^ cells in frontal cortex (*n* = 6). TMEM119: *F*(2, 30) = 20.87, *P* < 0.0001. CD68: *F*(2, 30) = 96.19, *P* < 0.0001. CD16/32: *F*(2, 30) = 10.13, *P* = 0.0004. (**C**) IHC staining images of IBA1^+^ microglia in 12-month-old mice. (**D**) IBA1^+^ microglia density and morphology in frontal cortex (*n* = 6). Density: *F*(2, 30) = 1.066, *P* = 0.3571. Process: *F*(2, 30) = 11.9, *P* = 0.0002. Soma: *F*(2, 30) = 23.57, *P* < 0.0001. (**E**) IBA1^+^ microglia density and morphology in the hippocampus of 12-month-old mice (*n* = 3, one-way ANOVA). Density: *F*(2, 6) = 12.27, *P* = 0.0076. Process: *F*(2, 6) = 0.8096, *P* = 0.4883. Soma: *F*(2, 6) = 53.16, *P* = 0.0002. The columns represent the mean ± SD. Statistical analysis was performed using two-way ANOVA unless specifically labelled. Density is calculated as cell count/area. Scale bar = 50 μm. TMEM119, transmembrane protein 119; CD68, cluster of differentiation 68; CD16/32, cluster of differentiation 16/32; IBA1, ionized calcium-binding adaptor molecule 1; WT, wild type; HIP, hippocampus; FC, frontal cortex. Each data point represents the mean of three technical replicates in one mouse. *P < 0.05; **P < 0.01; ***P < 0.001; ****P < 0.0001.

We explored age-dependent associations between glial and Aβ markers in 2.5-, 7- and 12-month-old *App*^NL-G-F^ and WT mice (*n* = 6 at each age point) in the hippocampus and frontal cortex. The total IBA1^+^ microglial density did not change significantly with age in either group (Fig. 4D), but there was a significant decrease of homeostatic TMEM119^+^ microglia density at 7 months (*P* < 0.05 for both *App*^NL-G-F^ and WT; Fig. 4A and B and Supplementary Figs 4A and 5A). There were increases in both CD68^+^ and CD16/32^+^ microglia in the 7- and 12-month-old *App*^NL-G-F^ mice relative to the levels in WT animals (Fig. 4A and B and Supplementary Figs 4A and 5B, C). At 7 and 12 months, microglia in *App*^NL-G-F^ mice had shorter processes and larger cell soma in both the hippocampus and frontal cortex than at 2.5 months, suggesting microglial activation (Fig. 4C and D and Supplementary Fig. 4B).

**Figure 5:**
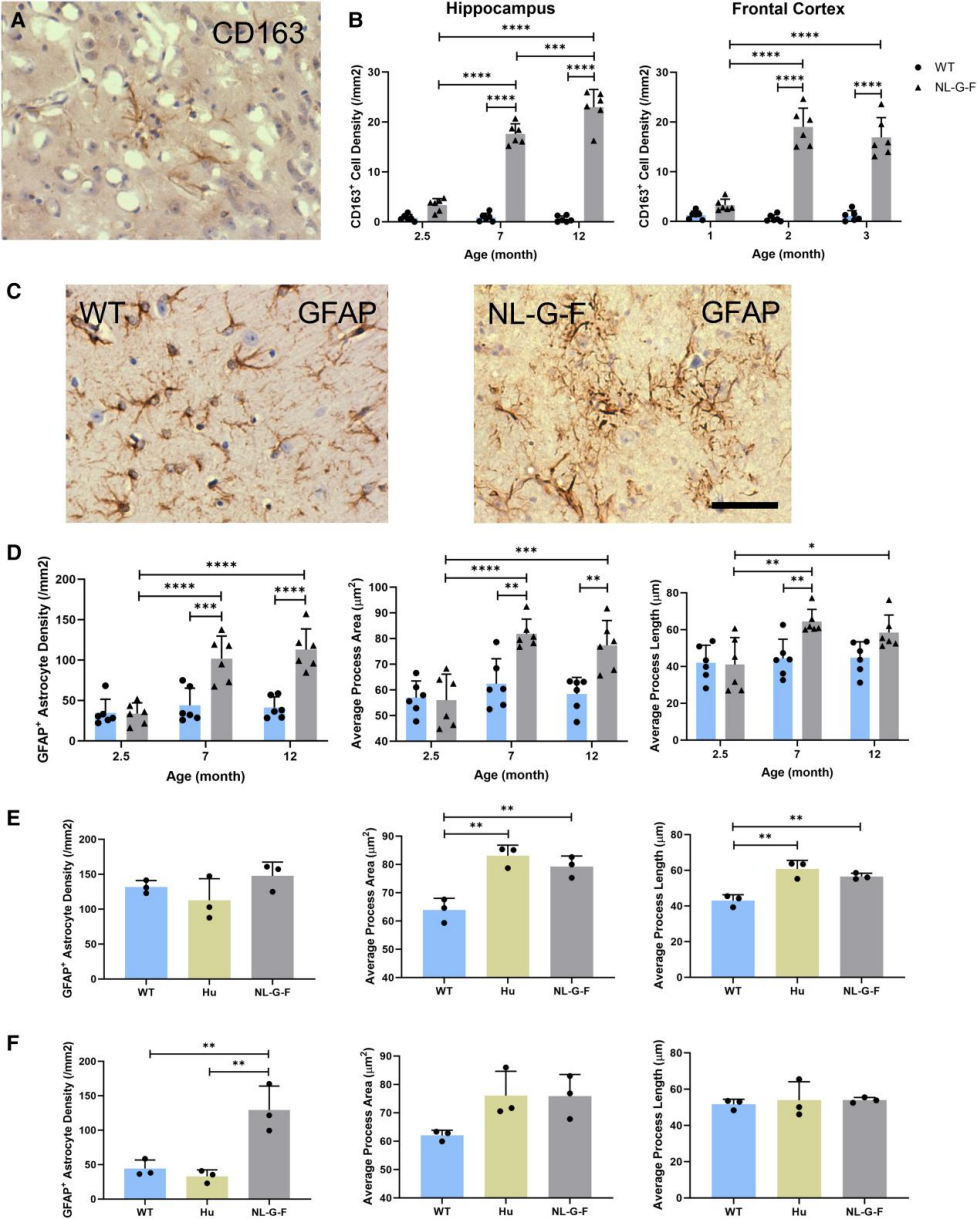
**Age-related changes of glial density and morphology in the HIP and FC of *App*^NL-G-F^, *App*^hu^ and WT mice.** (**A**) IHC staining images of CD163^+^ anti-inflammatory microglia and astrocytes in 12-month-old App^NL-G-F^ mice. (**B**) CD163^+^ cell density (*n* = 6). HIP: *F*(2, 30) = 94.49, *P* < 0.0001. FC: *F*(2, 30) = 41.2, *P* < 0.0001. (**C**) IHC staining images of GFAP^+^ astrocytes in 12-month-old mice. (**D**) GFAP^+^ astrocyte density and average process area in the frontal cortex (*n* = 6). Density: *F*(2, 30) = 10.51, *P* = 0.0003. Area: *F*(2, 30) = 6.009, *P* = 0.0064. Length: *F*(2, 30) = 3.337, *P* = 0.0491. (**E**) GFAP^+^ astrocyte density and morphology in the HIP of 12-month-old mice (*n* = 3, one-way ANOVA). Density: *F*(2, 6) = 1.924, *P* = 0.2261. Area: *F*(2, 6) = 20.41, *P* = 0.0021. Length: *F*(2, 6) = 20.84, *P* = 0.002. (**F**) GFAP^+^ astrocyte density and morphology in the FC of 12-month-old mice (*n* = 3, one-way ANOVA). Density: *F*(2, 6) = 17.39, *P* = 0.0032. Area: *F*(2, 6) = 4.32, *P* = 0.0688. Length: *F*(2, 6) = 0.1361, *P* = 0.8754. The columns represent the mean ± SD. Statistical analysis was performed using two-way ANOVA unless specifically labelled. Density is calculated as cell count/area. Scale bar = 50 μm. CD163, cluster of differentiation 163; GFAP, glial fibrillary acidic protein; WT, wild type; HIP, hippocampus; FC, frontal cortex. Each data point represents the mean of three technical replicates in one mouse. *P < 0.05; **P < 0.01; ***P < 0.001; ****P < 0.0001.

CD163^+^ cell density also increased with age in the hippocampus of *App*^NL-G-F^ mice; the frontal cortex staining for this marker was 5-fold higher in 7-month-old mice than in 2.5-month-old mice, although we did not find a further significant increase at 12 months (Fig. 5B). Whereas the majority of the CD163^+^ cells were microglia, a small proportion expressed GFAP^+^ and had an astrocyte-like morphology (Fig. 5A and Supplementary Fig. 5D and E). Phenotypic transition of microglia into astrocyte-like cells has been reported previously in a rodent neurodegeneration model.^27^ The total GFAP^+^ astrocyte density increased significantly with age in the brains of *App*^NL-G-F^ mice, with longer and thicker processes, especially in the frontal cortex, where the density increased ∼3-fold between 2.5 and 7 months of age (Fig. 5C and D and Supplementary Fig. 4C). Consistent with this observation, we found a progressive increase in PBR^+^ (suggesting activated microglia or astrocytes) cell density with greater NAB61^+^ Aβ oligomer area (Supplementary Figs 5F, G and 6A, B). Marker co-localizations show that, although the majority of PBR^+^ cells in 2.5-month-old mice were astrocytes, most PBR^+^ cells were microglia at 12 months (Supplementary Fig. 4D and E).

In contrast, the IBA1^+^ (Fig. 4E and Supplementary Fig. 4F), TMEM119^+^ (Supplementary Fig. 7A and B), CD16/32^+^ (Supplementary Fig. 7C and D), CD68^+^ (Supplementary Fig. 7E and F) and CD163^+^ (Supplementary Fig. 7G and H) microglia densities and morphology were similar in *App*^hu^ and WT mice at 12 months. GFAP^+^ and PBR^+^ cell densities also were not different, although the GFAP^+^ astrocytes in *App*^hu^ mice had significantly longer and thicker processes than in WT mice, similar to changes observed in 12-month-old *App*^NL-G-F^ mice (Fig. 5E and F and Supplementary Fig. 6A and C). This suggests that NAB61^+^ Aβ oligomers in the *App*^hu^ mice may activate astrocytes selectively.

### Spatial relationships between glial markers and Aβ pathology identified using IMC

**Figure 6:**
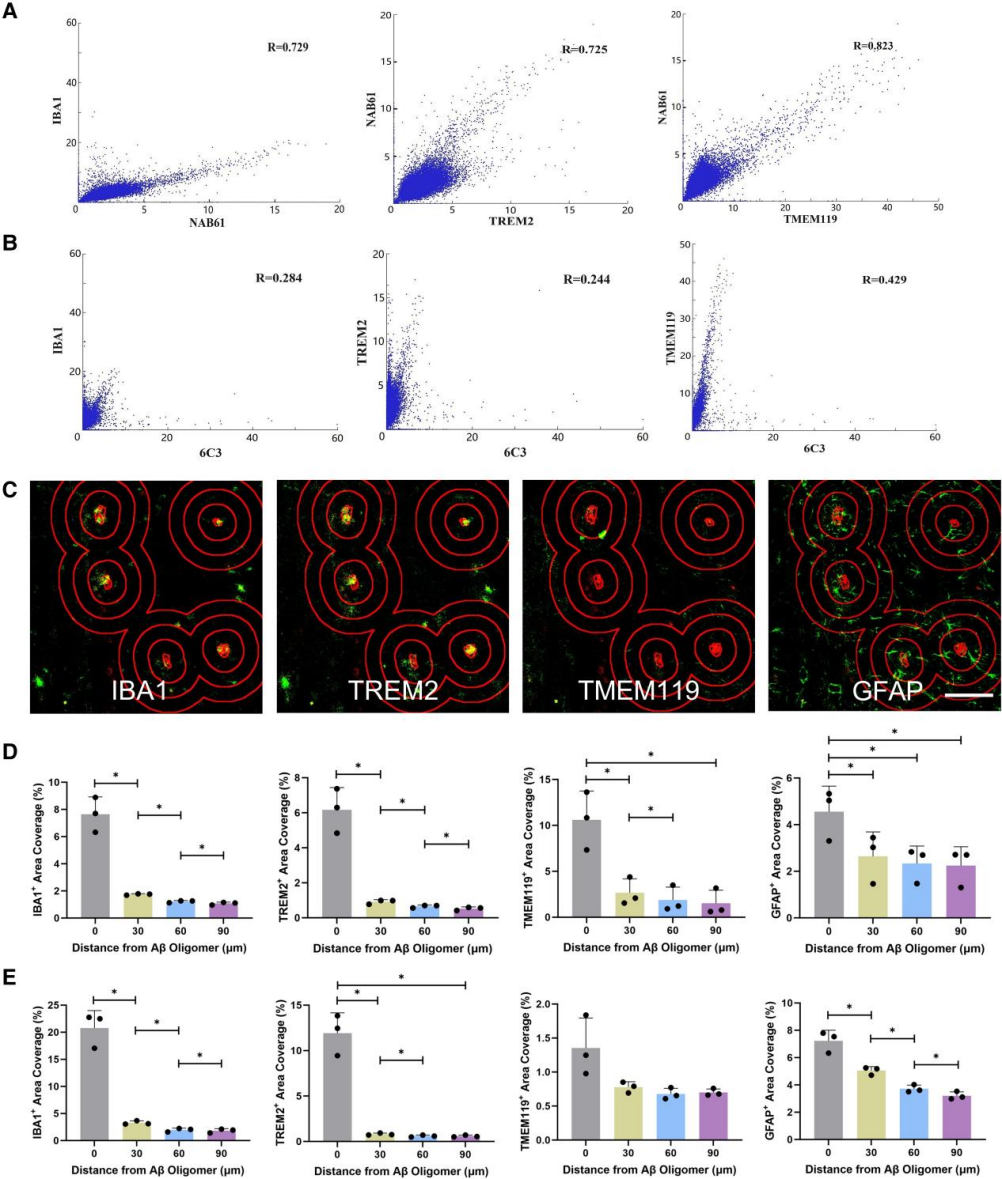
**A Sholl analysis of Aβ oligomers and glial markers in the hippocampus of *App*^NL-G-F^ mice (*n* = 3).** (**A**) Correlation plots between NAB61^+^ Aβ oligomers and microglia markers in 2.5-month-old mice. *P* < 0.0001 for all. (**B**) Correlation plots between 6C3^+^ Aβ plaques and microglia markers in 2.5-month-old mice. *P* < 0.0001 for all. (**C**) Representative IMC images with a Sholl analysis of NAB61^+^ Aβ oligomers (red) and glial markers (green) in 12-month-old mice. (**D**) A Sholl analysis of NAB61^+^ Aβ oligomers and glial markers in 2.5-month-old mice. IBA1: *F*(1.011, 2.023) = 66.55, *P* = 0.0142. TREM2: *F*(1.001, 2.001) = 64.98, *P* = 0.015. TMEM119: *F*(1.01, 2.02) = 53.49, *P* = 0.0177. GFAP: *F*(1.951, 3.902) = 71.46, *P* = 0.0009. (**E**) A Sholl analysis of NAB61^+^ Aβ oligomers and glial markers in 12-month-old mice. IBA1: F(1,2) = 95.75, *P* = 0.0103. TREM2: *F*(1.008, 2.016) = 80.58, *P* = 0.0119. TMEM119: *F*(1.008, 2.016) = 6.57, *P* = 0.1236. GFAP: *F*(1.102, 2.203) = 73.66, *P* = 0.0098. The columns represent the mean ± SD, statistical analysis was performed using one-way ANOVA. Ring distance = 30 μm. Scale bar = 100 μm. IBA1, ionized calcium-binding adaptor molecule 1; TREM2, triggering receptor expressed on myeloid cells 2; TMEM119, transmembrane protein 119; GFAP, glial fibrillary acidic protein. Each data point represents the mean of three technical replicates in one mouse. *P < 0.05.

We extended the observations above using IMC to study the spatial relationship between glial markers and Aβ pathology (Supplementary Fig. 2C). In 12-month-old mice, expression of NAB61^+^ Aβ oligomers showed moderate to strong correlations with all microglia phenotypic markers (IBA1, APOE, TMEM119, TREM2, CD68, CD163 and CD16/32) and immunoproteosome marker LMP7 (Supplementary Fig. 8A and B and Supplementary Table 8). Aβ plaques weakly correlated with those markers except for a correlation with TREM2 (Supplementary Fig. 8C and D and Supplementary Table 8). IBA1, TMEM119 and TREM2 expression levels correlated more strongly with NAB61^+^ Aβ oligomers than with Aβ plaques at 2.5 months, and these correlations were weaker at 12 months (Fig. 6A and B and Supplementary Table 8). Aβ plaque expression at 2.5 months showed weaker correlations with IBA1 and TREM2 compared with those in 12-month-old mice, whereas correlation with TMEM119 was stronger (Supplementary Table 8).

Sholl analyses were carried out to support the correlation analyses. This also provided evidence for closer proximity of NAB61^+^ Aβ oligomers and IBA1^+^, TREM2^+^ or TMEM119^+^ microglia than was found for the 6C3^+^ Aβ plaques in 2.5-month-old mice (*P* < 0.01, Supplementary Table 7, Fig. 6C and D and Supplementary Fig. 9A–F). In 12-month-old mice, proximity of IBA1^+^ microglia and TREM2^+^ microglia to both Aβ oligomers and Aβ plaques increased significantly than in 2.5-month-old mice (*P* < 0.0001, Supplementary Table 7, Fig. 6E and Supplementary Fig. 9G–J). Proximity of TMEM119^+^ microglia to NAB61^+^ Aβ oligomers decreased significantly from 2.5 to 12 months (*P* < 0.001), despite similar proximity to 6C3^+^ Aβ plaques (Supplementary Table 7 and Supplementary Fig. 9K and L). CD16/32^+^ proinflammatory microglia, APOE^+^ astrocytes and LMP7^+^ proteasomes in 12-month-old *App*^NL-G-F^ mice also had higher proximity to Aβ oligomers than to Aβ plaques (*P* < 0.05, Supplementary Table 7). However, no significant difference in relative proximities was found for GFAP^+^ or PBR^+^ cells, and they were both closer to Aβ oligomers and plaques in 12-month-old mice than in 2.5-month-old mice (*P* < 0.001, Supplementary Table 7). Generally, in 12-month *App*^NL-G-F^ mice, microglia were mostly proximal to Aβ oligomers and plaques, whereas astrocytes tended to surround amyloid species in a more scattered pattern (Fig. 6E).

### Identification of subtypes of microglia and astrocytes in 12-month-old *App*^NL-G-F^ mice

**Figure 7:**
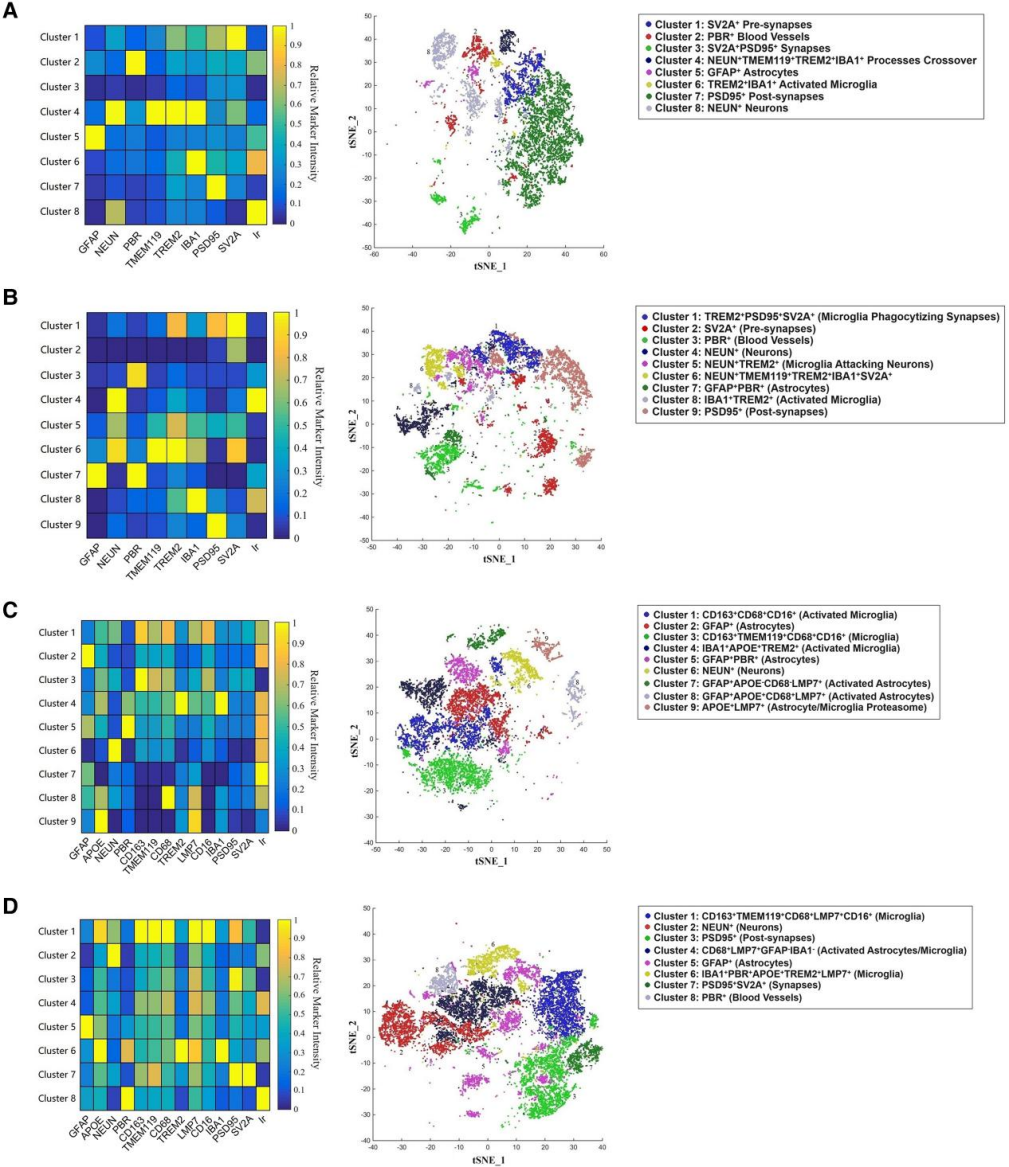
**A spatial IMC analysis of cellular markers in *App*^NL-G-F^ mice (*n* = 3).** (**A**, **B**) Heat map and phenograph clustering with *t*-distributed stochastic neighbour embedding (tSNE) in the hippocampus (**A**) and frontal cortex (**B**) of 2.5-month-old mice. (**C**, **D**) Heat map and phenograph clustering with tSNE in the hippocampus (**C**) and frontal cortex (**D**) of 12-month-old mice. GFAP, glial fibrillary acidic protein; APOE, apolipoprotein E; NEUN, neuronal nuclear protein; PBR, peripheral benzodiazepine receptor; CD163, cluster of differentiation 163; TMEM119, transmembrane protein 119; CD68, cluster of differentiation 68; TREM2, triggering receptor expressed on myeloid cells 2; LMP7, low-molecular mass protein-7; CD16, cluster of differentiation 16; IBA1, ionized calcium-binding adaptor molecule 1; PSD95, post-synaptic density protein 95; SV2A, synaptic vesicle glycoprotein 2A. Ir (intercalator) marks cell nuclei.

The pairwise correlations highlighted the multivariate relationships between markers expected from their functionally related pathways. We explored glial subtypes in the data using tSNE plots of cellular markers detected simultaneously in the IMC images. In 12-month-old *App*^NL-G-F^ mice, we defined nine main cell clusters based on their distinct marker phenotypes in the hippocampus, including two astrocytic clusters expressing the immunoproteasome marker LMP7 (GFAP^+^APOE^−^CD68^−^LMP7^+^- and GFAP^+^APOE^+^CD68^+^LMP7^+^-activated astrocytes, Fig. 7C and Supplementary Fig. 10A and B). We have also found IBA1^+^APOE^+^TREM2^+^-activated microglia, consistent with a disease-associated microglia (DAM) response in Alzheimer’s disease.^28^ CD163^+^CD68^+^CD16/32^+^-activated microglia, which are likely to show both proinflammatory and anti-inflammatory potential, were found as well. Eight main clusters were defined in the frontal cortex, including microglial clusters expressing LMP7 (CD68^+^LMP7^+^GFAP^−^IBA1^−^- and IBA1^+^PBR^+^APOE^+^TREM2^+^LMP7^+^-activated microglia, Fig. 7D and Supplementary Fig. 10C and D). In 2.5-month-old mice, we defined eight main clusters in the hippocampus, and nine main clusters in the frontal cortex, including TREM2^+^PSD95^+^SV2A^+^ and TREM2^+^NEUN^+^ microglial clusters that highlight potential microglial–neuronal interactions in the younger mice (Fig. 7A and B and Supplementary Fig. 10E and F).

## Discussion

Understanding relationships between neurodegeneration, synaptic loss and glial activation associated with different forms of aggregated pathological amyloidogenic proteins^29,30^ is important for the design of optimal therapies to reduce brain Aβ load in early Alzheimer’s disease. Here, we have used two mouse models, one expressing the human APP allele (*App*^hu^) and another expressing the human APP sequence with three variants that promote abnormal Aβ accumulation associated with early onset familial Alzheimer’s disease (*App*^NL-G-F^), to characterize relationships between NAB61^+^ Aβ oligomers or 6C3^+^ Aβ plaques and glial activation. We found that microglial activation correlated most strongly with age-related increases in NAB61^+^ Aβ oligomer expression in the *App*^NL-G-F^ model. Unexpectedly, we also found morphological evidence suggesting astrocyte activation with increased NAB61^+^ Aβ oligomer expression in the *App*^hu^ model. Reduced neuronal and synaptic densities near Aβ oligomers were observed in *App*^NL-G-F^ mice but not in *App*^hu^ mice. Most microglia in *App*^NL-G-F^ model expressed an IBA1^+^ or CD68^+^ activation phenotype and many showed an IBA1^+^APOE^+^TREM2^+^ DAM phenotype.^28^ Spatial proximity Sholl analyses provided evidence for a stronger association of Aβ oligomers (relative to Aβ plaques) with proinflammatory microglia; microglia expressing CD163 did not show differences in localization relative to Aβ oligomers and plaques. These data highlight a pathological role for Aβ oligomers (rather than Aβ plaques) in the early inflammatory activation with Aβ pathology. Our results also suggest that Aβ oligomers with conformations adopted by the WT human allele may activate astrocytes in the *App*^hu^ model, a mechanism that could contribute to the early astrocyte activation in Alzheimer’s disease.^31,32^

Both microglia and astrocytes show prominent activation signatures in the *App*^NL-G-F^ model. Microglia were mostly proximal to Aβ oligomers and plaques, whereas astrocytes tended to surround amyloid species in a more scattered pattern (Fig. 6C–E). GFAP^+^, GFAP^+^LMP7^+^ and GFAP^+^APOE^+^CD68^+^LMP7^+^ clusters constituted large proportions of the total astrocytes characterized. Microglia with a phagocytic DAM-like phenotype (IBA1^+^APOE^+^TREM2^+^ or IBA1^+^APOE^+^TREM2^+^PBR^+^LMP7^+^) were prominent. The App^hu^ model suggested astrocyte activation by Aβ oligomers generated from the human APP common allele, but this model was not associated with clear evidence for neuronal or synaptic pathology. Astrocyte activation, which is prominent *in vivo* in early Alzheimer’s disease in the absence of proinflammatory microglia activation may not be neurotoxic.^7,33^ Recent single nuclear transcriptomic characterization of astrocytes in Alzheimer’s disease showed that although nuclear factor κB and NLRP3 inflammatory pathways were upregulated with greater total tissue p-tau, Aβ expression was associated most strongly with increased expression of genes involved in metal ion homeostasis, chaperone functions and responses to unfolded proteins.^34^ This emphasizes the protective functions of astrocyte activation. In future work, astrocyte activation in the two models should be characterized to better define molecular phenotypes.

Synaptic loss, which has been well described in healthy ageing humans and rodents,^35,36^ is a strong correlate of cognitive deficits in patients with Alzheimer’s disease.^37,38^ In *App*^NL-G-F^ mice, prior work described synaptic impairment starting at 3–4 months,^39^ which is in line with our results of a significant difference between *App*^NL-G-F^ and WT mice at 7 months (Fig. 2B and C). Synaptic loss has also been observed in App transgenic mouse models independent of Aβ plaque formation,^40^ suggesting direct or indirect toxicities of Aβ oligomers. Aβ oligomers have been shown to bind specifically with stronger interactions to excitatory neurons.^41^ Aβ oligomers can activate microglia to phagocytize synapses via complement activation^42^ and may be directly neurotoxic.^43^ Here, we have also provided further evidence that aggregation-prone Aβ oligomers lead to proinflammatory activation of microglia, which release neurotoxic cytokines, complement and reactive oxygen species.^33^ Both are likely to contribute to neuronal and cognitive dysfunction in the *App*^NL-G-F^ model.^44,45^ Additionally, we made the incidental observation that Aβ pathology was associated with increased expression of CD163 in both microglia and astrocytes. The phenotypic transition of microglia into astrocyte-like cells was previously reported a study of brain injury and chronic neurodegeneration in a rodent model.^27,46^

A strength of our study is that we have used two Aβ models based on a common C57BL/6 genetic background, expressing APP under the control of the endogenous mouse App promoter, which facilitated their comparison. Future work may benefit from more detailed measures over the lifespan with App^hu^ mice. Aβ oligomers have been attracting our attention due to the neurotoxic properties, and our study is a novel and timely contribution to the field to claim the neurotoxicity on the basis of aggregation state.^47^ However, a limitation is that structural and conformational differences in the generated Aβ peptides can only be inferred, although there are undoubtedly differences. We also performed only a limited analysis of glial phenotypes based on classical immunohistological markers. This was particularly limited for astrocytes, the molecular phenotypes of which need to be described comprehensively in future work. The reliance on IMC for characterization of glial cells and markers, while powerful because it simultaneously allows multiple markers to be characterized, is limited by the lower sensitivity of IMC detection relative to that afforded by IF and imaging in a single plane only. This is expected to artificially lower cell numbers sampled and could lead to a bias towards the activated glia with their enlarged cell bodies and thicker processes. Finally, we can only speculate about the probable mechanisms of neuronal injury as direct toxicity of Aβ species and indirect toxicity from inflammatory factors could only be hypothesized.

## Conclusion

Our study has focused on the relationships between brain Aβ pathology, glial activation and neurodegeneration. Our results support evidence that the neurotoxicity of Aβ oligomers may be greater than that of Aβ plaques, which have been the main focus of human clinical imaging biomarker studies.^48,49^ They highlight how glial responses to Aβ oligomers can depend on the conformation or aggregation state of the oligomers.^50^ Therapeutic challenges for Alzheimer’s disease must be tackled by taking steps to further reduce concentrations of the most toxic oligomeric species,^51^ limit the post-translational modifications leading to toxic conformations^52^ and reduce neurotoxic glial inflammatory responses without compromising glial contributions to the clearance of Aβ oligomers.^1^

## Supplementary Material

fcae218_Supplementary_Data

## Data Availability

The authors will make images and quantification available to researchers on reasonable request.
