# Supplementary material for: Associations of amyloid-β oligomers and plaques with neuropathology in the AppNL-G-F mouse
Source: Brain Commun. 2024 Jun 25;6(4):fcae218. doi: 10.1093/braincomms/fcae218 (PMC11258573; doi:10.1093/braincomms/fcae218)
Supplement: fcae218_Supplementary_Data [file fcae218_supplementary_data.zip › Original_submission_manuscript.pdf]

OXFORD UNIVERSITY PRESS | Brain Communications

Neuropathology Associated with Amyloid-β Oligomers in the AppNL-G-F Mouse

Journal:	*Brain Communications*
Manuscript ID	BRAINCOM-2023-360
Manuscript Type:	Original Article
Date Submitted by the Author:	29-Aug-2023
Complete List of Authors:	Tang, Jiabin; UK Dementia Research Institute, Imperial College London; Imperial College London, Department of Brain Sciences; Cornell University, Weill Cornell Medicine<br>Huang, Helen; Imperial College London, Department of Metabolism, Digestion and Reproduction<br>Muirhead, Robert; UK Dementia Research Institute, Imperial College London; King's College London, Randall Centre for Cell & Molecular Biophysics<br>Zhou, Yue; University College London, Department of Mechanical Engineering<br>DeFelice, John; Imperial College London, Department of Brain Sciences<br>Kopanitsa, Maksym; UK Dementia Research Institute, Imperial College London; The Francis Crick Institute<br>Serneels, Lutgarde; Flanders Institute of Biotechnology Department of Medical Protein Research, Centre for Brain and Disease Research<br>Davey, Karen; UK Dementia Research Institute, Imperial College London; The Francis Crick Institute<br>Tilley, Bension; Imperial College London Centre for Neuroscience, Brain Sciences<br>Li, Junheng; UK Dementia Research Institute, Imperial College London<br>Gentleman, Steve; Imperial College London, Department of Brain Sciences<br>Matthews, Paul M; UK Dementia Research Institute, Imperial College London; Imperial College London, Department of Brain Sciences
Keywords:	Amyloid-β, oligomers, AppNL-G-F, imaging mass cytometry, neuropathology

SCHOLARONE™
Manuscripts

Neuropathology Associated with Amyloid-β Oligomers in the *App*[NL-G-F] Mouse

Jiabin Tang[1,2,6], Helen Huang[3], Robert C.J. Muirhead[1,9], Yue Zhou[4], John DeFelice[2], Maksym V. Kopanitsa[1,7], Lutgarde Serneels[5], Karen Davey[1,8], Bension S. Tilley[2], Junheng Li[1], Steve Gentleman[2]* & Paul M. Matthews[1,2]*

1. UK Dementia Research Institute, Uren Building, White City Campus, Imperial College London, London W12 0BZ, UK
2. Department of Brain Sciences, Burlington Danes Building, Hammersmith Campus, Imperial College London, London W12 0NN, UK
3. Department of Metabolism, Digestion and Reproduction, South Kensington Campus, Imperial College London, London SW7 2AZ, UK
4. Department of Mechanical Engineering, Roberts Engineering Building, University College London, London WC1E 7JE, UK
5. Centre for Brain and Disease Research, Flanders Institute for Biotechnology (VIB), Leuven, Belgium
6. Department of Anesthesiology, Weill Cornell Medicine, Cornell University, New York 11106, NY, USA
7. The Francis Crick Institute, London NW1 1AT, UK
8. UK Dementia Research Institute, Denmark Hill Campus, Kings College London, London SE5 9RX, UK
9. Randall Centre for Cell & Molecular Biophysics, Kings College London, London SE5 9RX, UK

*Corresponding authors: s.gentleman@imperial.ac.uk, p.matthews@imperial.ac.uk

For Review Only

Keywords: Amyloid-β, oligomers, *App*[NL-G-F], imaging mass cytometry, neuropathology

Abbreviations: Aβ = amyloid-β; AD = Alzheimer's disease; APOE = apolipoprotein E; APP = amyloid precursor protein; CD16/32 = cluster of differentiation 16/32; CD163 = cluster of differentiation 163; CD68 = cluster of differentiation 68; DAB = 3,3′-diaminobenzidine; DAM = disease-associated microglia; EDTA = ethylenediaminetetraacetic acid; GFAP = glial fibrillary acidic protein; IBA1 = ionized calcium binding adaptor molecule 1; IF = immunofluorescence; IHC = immunohistochemistry; IMC = imaging mass cytometry; Ip Kit = ImmPRESS Polymer Detection Kit; LMP7 = low-molecular mass protein-7; NEUN = neuronal nuclear protein; NF-κB = nuclear factor-κB; NLRP3 = NLR family pyrin domain containing 3; PBR = peripheral benzodiazepine receptor; PBS = phosphate-buffered saline; PBST = PBS containing 0.3% Triton; PSD95 = postsynaptic density protein 95; ROI = regions of interest;

SS Kit = Supersensitive Polymer HRP Kit; SV2A = synaptic vesicle glycoprotein 2A; TMEM119 = transmembrane Protein 119; TREM2 = triggering receptor expressed on myeloid cells 2; tSNE = t-distributed stochastic neighbor embedding; WT = wildtype;

Abstract

Amyloid-β (Aβ) pathology and neurofibrillary tangles lead to glial activation and neurodegeneration in Alzheimer's disease (AD). Here we have investigated relationships between the expression of Aβ oligomers, Aβ plaques, glial activation and markers related to neurodegeneration in the *App*$^{NL-G-F}$ triple mutation mouse line and in a knock-in line homozygous for the common human amyloid precursor protein (*App*^{hu} mouse). Relationships between neuropathological features were characterized with immunohistochemistry and imaging mass cytometry. Markers assessing human Aβ proteins, microglial and astrocytic activation, and neuronal and synaptic densities were used in mice between 2.5 and 12 months of age. We found that Aβ oligomers were abundant in the brains of *App*^{hu} mice in the absence of classical Aβ plaques. These brains showed morphological changes consistent with astrocyte activation but no evidence of microglial activation or synaptic or neuronal pathology. By contrast, both high levels of Aβ oligomers and numerous plaques accumulated in *App*$^{NL-G-F}$ mice in association with substantial astrocytic and microglial activation. The increase in Aβ oligomers over time were more strongly correlated with astrocytic than with microglia activation. Spatial analyses suggested that activated microglia were more closely associated with Aβ oligomers than with Aβ plaques in *App*$^{NL-G-F}$ mice, which also showed age-dependent decreases in neuronal and synaptic density markers. Comparative study of the two models highlights dependence of glial and neuronal pathology on the nature and aggregation state of the Aβ oligomeric species expressed.

For Review Only

Introduction

Amyloid-β (Aβ) pathology and tau neurofibrillary tangles, in association with glial activation and neurodegeneration, are hallmarks of AD.[1] Microglia maintain tissue homeostasis, but when activated by Aβ or phosphorylated tau, can adopt an pro-inflammatory phenotype promoting neurodegeneration.[2] Astrocyte activation can be associated with the release of cytokines, inflammatory factors and reactive oxygen species.[3-5] Single-cell RNA sequencing has demonstrated consistent transcriptional changes in microglial and astrocytes that are associated with the presence of Aβ pathology.[6,7]

There are various forms of Aβ protein in human brains, including monomers,

dimers, oligomers and fibrils.[8] Aβ fibrils tend to aggregate into plaques, which can be detected using *in vivo* PET imaging in AD patients, while Aβ oligomers can be hardly detected.[9,10] Different Aβ peptide conformations are associated with differential kinetics of aggregation or degradation.[11] It has been assumed by many that neurotoxic species are associated primarily with Aβ plaques, but this has not been explored widely in preclinical models, in part because it has been difficult to distinguish between consequences of increased levels of Aβ plaques and those of Aβ oligomers.[12,13] The availability of knock-in mouse models expressing wild-type or mutated forms of human amyloid precursor protein (APP), under the control of an endogenous mouse promoter, may help to address this problem. Investigating the relationships between peptide sequence and clinically-relevant neuropathological responses could help identify epitopes for new therapeutic antibodies.[14] Previous research suggested that Aβ oligomers accumulate around synapses.[15] This could promote synaptic loss in AD. This highlights the need to investigate cellular crosstalk between Aβ proteins, glia and neurons.

Here we have characterized brain cellular pathology in two mouse models. The *App*$^{NL\text{-}G\text{-}F}$ is a knock-in mouse model which avoids transgenic artefacts caused by transfecting multiple copies of the *APP* gene.[16] With humanization of the mouse *App* gene, the *App*$^{NL\text{-}G\text{-}F}$ mouse includes three functionally relevant mutations: the Swedish mutation which promotes the total Aβ protein production, the Beyreuther/Iberian mutation which increases the $A\beta_{42}/A\beta_{40}$ ratio, and the Arctic mutation which accelerates Aβ fibril assembly.[17,18] We also characterized brains of the *App*^{hu} mice, which express the human *APP* sequence under the mouse endogenous promoter.[19] We have studied the frontal cortex and hippocampus, two areas that are severely affected in AD brain. Cognitive dysfunction in AD is associated with dysconnectivity between hippocampus and frontal cortex;[20,21] amnestic and spatial memory defects also depend largely on the connection of these two regions in the *App*$^{NL\text{-}G\text{-}F}$ mouse.[22]

In this study, immunohistochemistry (IHC) and imaging mass cytometry (IMC) have both been used to image histological expression of different Aβ forms, and their spatial relationship with neuronal and glial cells displaying different functional phenotypes. We employed the NAB61 antibody, which targets potentially disease-relevant Aβ oligomers to complement the broader range of antibodies recognizing epitopes on higher order aggregates and plaques.[23]

Materials & Methods

Mouse Tissue Preparation

For Review Only

Ethical Approval

Mouse brains were obtained under protocols approved by Animal Welfare and Ethical Review Bodies of the Medical Research Council Harwell Institute and Charles River UK Ltd.

Mouse Breeding

WT and *App*$^{NL\text{-}G\text{-}F}$ mice (or *App*$^{tm3.1Tcs}$ mice, MGI: 5637817) were housed in specific-pathogen-free condition (Mary Lyon Centre, MRC Harwell) and specific-and-opportunistic-pathogens-free (Charles River UK) condition adhering to environmental conditions as outlined in the UK Home Office Code of Practice. *App*^{hu} mice (or *App*^{em1Bdes} mice, MGI: 6512851) were housed in specific-pathogen-free condition in KU Leuven (Belgium). All animals were on the C57Bl/6J background. All animals had ad libitum access to water and standard rodent food, and were kept on a 12-h light and dark cycle. All animal experiments were performed in accordance with UK Home Office Project Licenses for breeding genetically altered animals at the Medical Research Council Harwell Institute and Charles River UK Ltd.

Sample Size

Sample size was estimated based on previous experiments performed in the laboratory.[24] All experiments with n=6 were carried out with 3 male and 3 female mice. Experiments with n=3 were carried out with male mice only. All experiments were randomized to avoid sex, litter, and batch effects. Investigators were blinded when performing all experiments.

Tissue Preparation

The mice were euthanized by sodium pentobarbital injection, exsanguinated, and their brains were quickly dissected free over ice. Then, a transverse cut along the midline was used to separate the hemispheres. Right hemispheres were used for this study, and were post-fixed in 4% paraformaldehyde for 24 h at 4 °C. Then, after a brief wash with phosphate-buffered saline (PBS), they were cryoprotected in 30% sucrose in PBS for 2–3 days until sunk, and kept in the same solution at 4 °C. Finally, the brains were immersed into optimum cutting temperature compound (Tissue-Tek 4583) and snap frozen on dry ice. The cryostat (Leica, CM1900) was used to section the tissue at 10 μm, and the slides were stored at −80 °C until further use.

Genotype Confirmation

DNA Extraction & PCR

The DNA was extracted with DNeasy Blood & Tissue Kits (Qiagen 69504) according to the manufacture's instructions. DNA concentration was then tested with Nanodrop Spectrophotometer.

For Review Only

The PCR reaction was carried out with the Q5 High-Fidelity 2X Master Mix (New England Biolabs M0492S), and the manufacturer's setup protocol for a 25 µL reaction was followed (Supplementary Table 1 and 2). Three primers were used according to Saito et al. [16], including 5′-ATCTCGGAAGTGAAGATG-3′ (WT primer), 5′-ATCTCGGAAGTGAATCTA-3′ (*App*$^{NL-G-F}$ primer) and 5′-TGTAGATGAGAACTTAAC-3 (common primer). For the bioanalyzer step, the template DNA was diluted to a concentration of 10–20 ng/µL. For electrophoresis step, 5 µL template DNA was added. The assembly of all reaction components was carried out quickly at RT, and the PCR tubes were transferred to a thermal cycler (BIO-RAD, C1000).

<u>Bioanalyzer</u>
The Select-a-Size DNA Clean & Concentrator Kit (Zymo Research, D4080) was used as per manufacturer's instructions. For a 636 bp DNA sample, 25 µL of the DNA solution was added to 125 µL of the Select-a-Size DNA Binding Buffer. After DNA elution, the High Sensitivity DNA Kit (Agilent, 5067-4626) was used, and the DNA high sensitivity bioanalyzer chips were run in accordance with the manufacturer's guidelines. The chips were put into an Agilent 2100 Bioanalyzer after vortexing on a IKA vortex mixer for 1 min at 2400 rpm. The instrument's software was used and the program for dsDNA was chosen. After ~45 min run, the data was exported.

<u>Electrophoresis</u>
To prepare a 1.5% gel, 1.5 g agarose was added to 100 mL of Tris-Borate-EDTA. Then, 5 µL of Gel Loading Dye (Biolabs, B7024S) was added to each 25 µL DNA sample, and Quick-Load 100 bp DNA ladder (Biolabs, N0551G) was added to the ladder well. Next, the gel was run at 120 V for 20–30 min. Finally, the gel was placed into a UVP BioDoc-It Imaging System.

For Review Only

IHC Staining
The IHC 3,3′-diaminobenzidine (DAB) staining was carried out with two different kits, Supersensitive Polymer HRP Kit (BioGenex) or ImmPRESS Polymer Detection Kit (Vector). Three sections separated by ~300 µm were selected in the hippocampus and frontal cortex separately in each mouse to represent the whole region. The on-slide sections were air dried for at least 1 h, and put into three changes of PBS (5 min each). Then, the sections were incubated for 30 minutes in PBS containing 0.3% H_2O_2. Next, the sections were subsequently rinsed in distilled water (5 min) and PBS (3 × 5 min) before further procedures. The primary antibodies were diluted with PBS containing 0.3% Triton (PBST), and the sections were incubated with primary antibodies overnight at 4 °C. The primary antibody selection and dilution, as well as the incubation time of DAB are shown in Supplementary

Table 3. No antigen retrieval step was used.

Supersensitive Polymer HRP Kit (SS Kit)

Following incubation with primary antibodies in a humid chamber, the sections were incubated with Super Enhancer Reagent for 20 min, and Polymer-HRP for 30 min. Sections were washed twice for 5 min with PBS between each step, and the sections were visualized with DAB at RT after three 5-min PBS washes. Subsequently, the sections were washed with distilled water (2 × 5 min), and incubated in hematoxylin (Mayer, MHS32-1L) for about ~1 min before being rinsing with tap water for 5 min. Finally, after dehydration (70%, 90%, 100%, 100% industrial methylated spirit; 3 min for each step) and clearing steps (three changes of 100% xylene, 5 min each), the sections were coverslipped with DPX mountant.

ImmPRESS Polymer Detection Kit (Ip Kit)

Sections were treated with horse or goat serum (according to the host species of secondary antibodies) for 20 min prior to immediate incubation with primary antibodies in a humid chamber. Following this, the tissue was washed in PBS and appropriate secondary antibodies were applied for 30 min. Subsequently, the tissue was washed in PBS (3 × 5 min) and visualized with DAB at RT. Finally, the hematoxylin, dehydration and clearing steps were performed as above.

Immunofluorescence (IF) Staining

Sections were air dried for at least 1 h and washed with PBS (3 × 5 min each). Following antigen retrieval (Supplementary Table 4), the sections were incubated at 4 °C overnight or RT for 2.5 h in a solution with a mixture of primary antibodies after subsequent wash in distilled water (5 min) and PBS (3 × 5 min). The list of primary antibodies, dilutions and incubation times are shown in Supplementary Table 4. Next, sections were washed twice in PBS and incubated with appropriate secondary antibodies (Supplementary Table 5) for 60 min. After washing with PBS (3 × 5 min each), 0.4% Sudan Black (Thermo Fisher Scientific, 4197-25-5) in 70% industrial methylated spirit was applied for 10 min to reduce the autofluorescence. Finally, the sections were rinsed with distilled water for 15 min and mounted with Antifade Mounting Media containing 4′,6-diamidino-2-phenylindole (Vector, H-1200).

IMC

IMC is an advanced technology combining a novel laser ablation system with mass cytometry that allows visualization of the simultaneous expression of up to 40 markers in the same section, providing a powerful tool to study spatial relationships between proteins.[25]

For Review Only

Antibody Conjugation with Metal

The process of antibody conjugation with metal was carried out with Maxpar X8 Antibody Labeling Kits (Fluidigm, 201300) according to the User Guides. All of the solution cocktails were mixed thoroughly before centrifugation or incubation.

95 µL of L-Buffer was added to the X8 polymer tube for resuspension, and 5 µL of Ln metal solution was added before incubation at 37 °C for 40 min in a water bath. Next, the mixture was added to a 3 kDa filter unit, with another 200 µL of L-Buffer added before centrifugation at 12,000×g for 25 min at RT. Then, 400 µL of C-Buffer was added before another centrifugation at 12,000×g for 30 min at RT.

Next, 100 µg of the antibody was loaded onto a 50 kDa filter, and the total volume was adjusted to 400 µL with R-Buffer before centrifugation at 12,000×g for 10 min at RT. Next, 100 µL of a freshly prepared 4 mM TCEP solution (MilliporeSigma, 646547) was added before incubation at 37 °C for 30 min in a water bath for antibody reduction. Then, 300 µL of C-Buffer was added immediately after the incubation before centrifugation at 12,000 g for 10 min at RT, and another 400 µL of C-Buffer was added with a repeated centrifugation step.

The purified Ln-loaded polymer and purified partially reduced antibody were retrieved separately, and the Ln-loaded polymer was resuspended with 60 µL of C-Buffer before mixing with a corresponding partially reduced antibody. The mixture was then incubated at 37 °C for 90 min in a water bath for conjugation. Next, 200 µL of W-Buffer was used to wash the conjugation mixture with centrifugation at 12,000×g for 10 min, with three washes with 400 µL of W-Buffer, each followed by centrifugation. After the final wash with W-buffer, 80 µL of W-buffer was added to dilute the conjugate for protein quantification, and another centrifugation at 12,000×g for 10 min was carried out to remove W-Buffer. 50 kDa columns were used for the centrifugation steps. Finally, Antibody Stabilizer PBS (Boca Scientific, 131 050) with 0.05% sodium azide (MilliporeSigma, 71289) was added to the conjugated antibody to obtain a final 0.5 mg/mL solution.

Protein Quantification was carried out using the Qubit Protein Assay (Thermo Fisher Scientific, Q33212). Protein buffer and dye were mixed at a ratio of 200:1. Three Qubit Protein Standards (10 µL of each) were added to 190 µL of the mixture separately for calibration in sequence, and 2 µL of the sample was added to 198 µL of the mixture for quantification. Afterwards, the quantification was carried out with a Qubit 4 Fluorometer (Thermo Fisher Scientific, Q33226).

For Review Only

Staining & Metal Detection

Three sections separated by 300 µm between each other were selected in the hippocampus and frontal cortex in each mouse to represent the whole region. The tissue sections were air dried for at least 2 h, and washed with PBS (3 × 5 min). Then, followed by heating with EDTA (pH=8) at 96 °C for 20 min, the sections were incubated in a primary antibody cocktail (Supplementary Table 6) overnight at 4 °C. The primary antibodies were diluted with 0.5% bovine serum albumin in PBST. After washing with PBS (2 × 8 min), Intercalator-Ir (Fluidigm, 201192A, 1:400) was applied for 30 min at RT for nuclei staining. Finally, the sections were washed with distilled water (2 × 5 min), and air-dried for at least 2 h before IMC ablation and metal detection with Helios System connected to Hyperion Imaging System (Fluidigm). Image processing was performed with MCD Viewer and ImageJ (Fiji, version 2.1.0), and images were exported in Tiff format.

Pixel Classification & Single Cell Segmentation

All of the image channels were merged with ImageJ, and put into Grayscale with the extended macros provided by Stephen Rothery in Imperial FILM Facilities. Only the Ir channel, indicating nuclear labeling, was left blue. Then, the composite images were saved in Jpeg format and processed with Ilastik 1.3 (University of Heidelberg, Germany) for pixel classification. The Ilastik was trained manually by selecting pixels of interest to identify and differentiate signal, nuclei or background. Finally, probability maps were created and exported in the Tiff format. CellProfiler 4.2.1 (Broad Institute, USA) was then used to process the probability maps. Seven modules were added, including Color To Gray, Identify Primary Objects, Identify Secondary Objects, Identify Tertiary Objects, Mask Objects, Convert Objects To Image and Save Images. The images were then analyzed automatically to create a mask for single cell segmentation.

Microscopy

IHC representative images were captured by a light microscope (Vanox, AHBT3) using a 20× objective. IHC images for quantitative analysis were captured with Digital Pathology Slide Scanners (Leica, Aperio AT2) using a 20× objective. IF images were captured by a Zeiss Axio Observer Inverted Microscope (Carl Zeiss Limited) in the FILM Facility of Imperial College London with a 20× objective, which was controlled by Zen acquisition software.

Statistical Analysis

IHC Analysis

The cell counting, process length, process area and soma area were done using Halo v2.1 software. The analysis plan was set before the experiments. The data analysis was performed with one-way or two-way ANOVA

For Review Only

(illustrated in figures) using GraphPad Prism 8.4 software.

Phenograph & Correlation Plots

HistoCAT 1.73 (University of Zurich, Switzerland) was used to run t-SNEs, phenographs, heatmaps and correlation plots. The cell mask was saved in the same folder with all correlated Tif images exported from ImageJ. The whole folders were then loaded to histoCAT, and t-SNEs as well as phenographs were run to differentiate the cells into different clusters. Then, heatmaps were created to show how the clusters were defined, and correlation plots were used to show the spatial relationships between two specific cell markers. Pearson correlation analysis was then carried out. Correlation was defined by 4-pixel expansion.

Sholl Analysis

IMC images were processed with ImageJ 2.1.0 (National Institute of Health, USA). The regions of interest (ROIs) of Aβ plaques or oligomers were manually selected, and added to ROI Manager. A code was then run to enlarge ROIs with the same distance (Supplementary File). The number of required rings was set to be 3, and the thickness of rings was set to be 30 µm. The area coverage % in each ring was then measured. The data analysis was processed with GraphPad Prism 8.4 software.

Results

Differences in the abundance of Aβ oligomers and plaques in the *App*$^{NL-G-F}$ and *App*^{hu} mice. We first assessed Aβ plaque and Aβ oligomer staining in the hippocampus and frontal cortex of 2.5-, 7- and 12-month-old mice (n=6 for both *App*$^{NL-G-F}$ and WT mice at each age). Aβ plaques with a variably dense appearance (Fig. 1A) increased in the *App*$^{NL-G-F}$ mouse by 2~3-fold between 2.5 and 7 months, without further significant change at 12 months (Fig. 1C). The Aβ oligomer staining (Fig. 1B) increased progressively between 2.5 and 12 months (Fig. 1D). NAB61$^+$ Aβ oligomers were localized in or immediately around plaques (Figure 1E and Supplementary Table 7).

Highly dense, process-like oligomeric Aβ staining was observed in 12-month-old *App*^{hu} mice (Fig. 1A and B). There was a significant difference between 6C3$^+$ and NAB61$^+$ areas in *App*$^{NL-G-F}$ mice (Fig. 1F), indicating predominant staining of Aβ plaques. However, in *App*^{hu} mice, the areas stained for Aβ oligomers and plaques were similar (Fig. 1F), with process-like staining mostly attributable to Aβ oligomers. No specific Aβ staining was seen with either antibody in the hippocampus or frontal cortex of WT mice.

For Review Only

Neuronal and synaptic loss was independent of Aβ plaque load in *App*$^{NL-G-F}$ mice. We assessed synaptic and neuronal staining in the hippocampus and frontal cortex of 2.5-, 7- and 12-month-old mice (Figure 2A). At 7 months, WT mice had significantly higher SV2A^{+} and PSD95^{+} staining than *App*$^{NL-G-F}$ mice (Fig. 2B and Supplementary Fig. 1A). At the same timepoint, the average neuronal soma areas and NEUN^{+} optical density were significantly lower in *App*$^{NL-G-F}$ mice, suggesting neuronal dystrophy (Fig. 2C and Supplementary Fig. 1B).[26] No substantial differences were seen in synaptic or neuronal staining in the samples from *App*$^{NL-G-F}$ and WT mice at 12 months. We also assessed these measures in the *App*^{hu} mice, in which we found an approximately 13% decrease in PSD95^{+} staining optical density in the hippocampus of *App*^{hu} mice compared to that in WT mice (Fig. 2D and E and Supplementary Fig. 1C and D). This was not accompanied by a significant change in SV2A staining optical density.

Spatial proximity Sholl analysis of NAB61^{+} Aβ oligomers and neuronal markers in *App*$^{NL-G-F}$ mice. IMC and Sholl analyses were used to explore the spatial relationships between Aβ pathology and neuronal or synaptic markers in 2.5- and 12-month-old *App*$^{NL-G-F}$ mice (n=3, male) in the hippocampus and frontal cortex (Fig. 3A and Supplementary Fig. 1E). At 2.5 months, mice showed a high degree of proximity of neuronal and synaptic marker staining signals to NAB61^{+} Aβ oligomers (Fig. 3B and Supplementary Fig. 2A, E, I). 6C3^{+} Aβ staining was also more abundant near SV2A^{+} synapses, but this trend was not found with NEUN^{+} neurons or PSD95^{+} synapses (Supplementary Fig. 2B, F and J). However, by 12 months, there was lower proximity of neuronal and synaptic markers with NAB61^{+} Aβ staining (Fig. 3C and Supplementary Fig. 2C, G and K). There was also a trend for lower colocalization of SV2A^{+} and PSD95^{+} synapses with 6C3^{+} Aβ staining, potentially as a consequence of local synaptic loss and neuronal dystrophy (Supplementary Fig. 2D, H and L).

Differences in associations of Aβ with glial activation in *App*$^{NL-G-F}$ and *App*^{hu} mice. We explored age-dependent associations between glial and Aβ markers in 2.5-, 7- and 12-month-old *App*$^{NL-G-F}$ and WT mice (n=6 at each age point) in the hippocampus and frontal cortex. The total IBA1^{+} microglial density did not change significantly with age in either group (Figure 4H), but there was a significant decrease of homeostatic TMEM119^{+} microglia density at 7 months ($P < 0.05$ for both *App*$^{NL-G-F}$ and WT) (Fig. 4A, B and Supplementary Fig. 3A, 4A). There were increases in both CD68^{+} and CD16/32^{+} microglia in the 7- and 12-month-old *App*$^{NL-G-F}$ mice relative to the levels in WT animals (Fig. 4C–F and Supplementary Fig. 3A, 4B, 4C). At 7 and 12 months, microglia in *App*$^{NL-G-F}$ mice had shorter processes and larger cell soma in both the hippocampus and frontal cortex than at 2.5 months, suggesting microglial activation (Fig. 4G, H and Supplementary Fig.

3B).

CD163+ cell density also increased with age in the hippocampus of *App*$^{NL-G-F}$ mice; the frontal cortex staining for this marker was 5-fold higher in 7-month-old mice than in 2.5-month-old mice, although we did not find a further significant increase at 12 months (Fig. 5B). Whereas the majority of the CD163+ cells were microglia, a small proportion expressed GFAP+ and had an astrocyte-like morphology (Fig. 5A and Supplementary Fig. 4D, E). Phenotypic transition of microglia into astrocyte-like cells has been reported previously in a rodent neurodegeneration model.[27] The total GFAP+ astrocyte density increased significantly with age in the brains of *App*$^{NL-G-F}$ mice, with longer and thicker processes, especially in the frontal cortex, where the density increased approximately 3-fold between 2.5 and 7 months of age (Fig. 5C, D and Supplementary Fig. 3C). Consistent with this observation, we found a progressive increase in PBR+ (suggesting activated microglia or astrocytes) cell density with greater NAB61+ Aβ oligomer area (Supplementary Fig. 4F, G and 5A, B). Marker co-localizations show that, although the majority of PBR+ cells in 2.5-month-old mice were astrocytes, most PBR+ cells were microglia at 12 months (Supplementary Fig. 3D and E).

By contrast, the IBA1+ (Fig. 5E and Supplementary Fig. 3F), TMEM119+ (Supplementary Fig. 6A and B), CD16/32+ (Supplementary Fig. 6C and D), CD68+ (Supplementary Fig. 6E and F) and CD163+ (Supplementary Fig. 6G and H) microglia densities and morphology were similar in *App*^{hu} and WT mice at 12 months. GFAP+ and PBR+ cell densities also were not different, although the GFAP+ astrocytes in *App*^{hu} mice had significantly longer and thicker processes than in WT mice, similar to changes observed in 12-month-old *App*$^{NL-G-F}$ mice (Fig. 5F, Supplementary Fig. 3G and 5A and C). This suggests that NAB61+ Aβ oligomers in the *App*^{hu} mice may activate astrocytes selectively.

Spatial relationships between glial markers and Aβ pathology identified using IMC. We extended the observations above using IMC to study the spatial relationship between glial markers and Aβ pathology (Supplementary Fig. 1E). In 12-month-old mice, expression of NAB61+ Aβ oligomers showed moderate to strong correlations with all microglia phenotypic markers (IBA1, APOE, TMEM119, TREM2, CD68, CD163, CD16/32) and immunoproteosome marker LMP7 (Supplementary Fig. 7A, B and Supplementary Table 8). Aβ plaques weakly correlated with those markers except for a correlation with TREM2 (Supplementary Fig. 7C, D and Supplementary Table 8). IBA1, TMEM119 and TREM2 expression levels correlated more strongly with NAB61+ Aβ oligomers than with Aβ plaques at 2.5 months, and these correlations were weaker at 12 months (Fig. 6A, B

For Review Only

and Supplementary Table 8). Aβ plaque expression at 2.5 months showed weaker correlations with IBA1 and TREM2 compared with those in 12-month-old mice, whereas correlation with TMEM119 was stronger (Supplementary Table 8).

Sholl analyses were carried out to support the correlation analyses. This also provided evidence for closer proximity of NAB61^{+} Aβ oligomers and IBA1^{+}, TREM2^{+} or TMEM119^{+} microglia than was found for the 6C3^{+} Aβ plaques in 2.5-month-old mice ($P < 0.01$, Supplementary Table 7, Fig. 6C, D and Supplementary Fig. 8A–F). In 12-month-old mice, proximity of IBA1^{+} microglia and TREM2^{+} microglia to both Aβ oligomers and Aβ plaques increased significantly than in 2.5-month-old mice ($P < 0.0001$, Supplementary Table 7, Fig. 6E and Supplementary Fig. 8G–J). Proximity of TMEM119^{+} microglia to NAB61^{+} Aβ oligomers decreased significantly from 2.5 to 12 months ($P < 0.001$), despite similar proximity to 6C3^{+} Aβ plaques (Supplementary Table 7 and Supplementary Fig. 8K, L). CD16/32^{+} proinflammatory microglia, APOE^{+} astrocytes and LMP7^{+} proteasomes in 12-month-old *App*$^{\text{NL-G-F}}$ mice also had higher proximity to Aβ oligomers than to Aβ plaques ($P < 0.05$, Supplementary Table 7). However, no significant difference in relative proximities were found for GFAP^{+} or PBR^{+} cells, and they were both closer to Aβ oligomers and plaques in 12-month-old mice than in 2.5-month-old mice ($P < 0.001$, Supplementary Table 7). Generally, in 12-month *App*$^{\text{NL-G-F}}$ mice, microglia were mostly proximal to Aβ oligomers and plaques, whereas astrocytes tended to surround amyloid species in a more scattered pattern (Fig. 6E).

Identification of subtypes of microglia and astrocytes in 12-month-old *App*$^{\text{NL-G-F}}$ mice. The pairwise correlations highlighted the multivariate relationships between markers expected from their functionally related pathways. We explored glial subtypes in the data using tSNE plots of cellular markers detected simultaneously in the IMC images. In 12-month-old *App*$^{\text{NL-G-F}}$ mice, we defined nine main cell clusters based on their distinct marker phenotypes in the hippocampus, including two astrocytic clusters expressing the immunoproteasome marker LMP7 (GFAP^{+}APOE^{-}CD68^{-}LMP7^{+} and GFAP^{+}APOE^{+}CD68^{+}LMP7^{+} activated astrocytes, Fig. 7A and Supplementary Fig. 9A, B). We have also found IBA1^{+}APOE^{+}TREM2^{+} activated microglia, consistent with a disease-associated microglia (DAM) response in AD.[28] CD163^{+}CD68^{+}CD16/32^{+} activated microglia, which are likely to show both proinflammatory and anti-inflammatory potential, were found as well. Eight main clusters were defined in the frontal cortex, including microglial clusters expressing LMP7 (CD68^{+}LMP7^{+}GFAP^{-}IBA1^{-} and IBA1^{+}PBR^{+}APOE^{+}TREM2^{+}LMP7^{+} activated microglia, Fig. 7B and Supplementary Fig. 9C, D). In 2.5-month-old mice, we defined eight main

For Review Only

clusters in the hippocampus, and nine main clusters in the frontal cortex, including TREM2^{+}PSD95^{+}SV2A^{+} and TREM2^{+}NEUN^{+} microglial clusters that highlight potential microglial-neuronal interactions in the younger mice (Fig. 7C, D and Supplementary Fig. 9E, F).

Discussion

Understanding relationships between neurodegeneration, synaptic loss and glial activation associated with different forms of aggregated pathological amyloidogenic proteins[29,30] is important for the design of optimal therapies to reduce brain Aβ load in early AD. Here, we have used two mouse models, one expressing the human APP allele (*App*^{hu}), and another expressing the human APP sequence with three variants that promote abnormal Aβ accumulation associated with early onset familial AD (*App*$^{NL\text{-}G\text{-}F}$), to characterize relationships between NAB61^{+} Aβ oligomers or 6C3^{+} Aβ plaques and glial activation. We found that microglial activation correlated most strongly with age-related increases in NAB61^{+} Aβ oligomer expression in the *App*$^{NL\text{-}G\text{-}F}$ model. Unexpectedly, we also found morphological evidence suggesting astrocyte activation with increased NAB61^{+} Aβ oligomer expression in the *App*^{hu} model. Reduced neuronal and synaptic densities near Aβ oligomers was observed in *App*$^{NL\text{-}G\text{-}F}$ mice but not in *App*^{hu} mice. Most microglia in *App*$^{NL\text{-}G\text{-}F}$ model expressed an IBA1^{+} or CD68^{+} activation phenotype and many showed an IBA1^{+}APOE^{+}TREM2^{+} DAM phenotype [28]. Spatial proximity Sholl analyses provided evidence for a stronger association of Aβ oligomers (relative to Aβ plaques) with proinflammatory microglia; microglia expressing CD163 did not show differences in localization relative to Aβ oligomers and plaques. These data highlight a pathological role for Aβ oligomers (rather than Aβ plaques) in the early inflammatory activation with Aβ pathology. Our results also suggest that Aβ oligomers with conformations adopted by the WT human allele may activate astrocytes in the *App*^{hu} model, a mechanism that could contribute to the early astrocyte activation in AD.[31,32]

Both microglia and astrocytes show prominent activation signatures in the *App*$^{NL\text{-}G\text{-}F}$ model. GFAP^{+}, GFAP^{+}LMP7^{+} and GFAP^{+}APOE^{+}CD68^{+}LMP7^{+} clusters constituted large proportions of the total astrocytes characterized. Microglia with a phagocytic DAM-like phenotype (IBA1^{+}APOE^{+}TREM2^{+} or IBA1^{+}APOE^{+}TREM2^{+}PBR^{+}LMP7^{+}) were prominent. The App^{hu} model suggested astrocyte activation by Aβ oligomers generated from the human APP common allele, but this model was not associated with clear evidence for neuronal or synaptic pathology. Astrocyte activation, which is prominent in vivo in early AD in the absence of proinflammatory microglia activation may not be neurotoxic.[7,33] Recent single nuclear transcriptomic characterization of astrocytes in AD showed that, although NF-κB and

NLRP3 inflammatory pathways were upregulated with greater total tissue p-tau, Aβ expression was associated most strongly with increased expression of genes involved in metal ion homeostasis, chaperone functions and responses to unfolded proteins.[34] This emphasizes protective functions of astrocyte activation. In future work, astrocyte activation in the two models should be characterized to better define molecular phenotypes.

Synaptic loss, which has been well described in healthy aging humans and rodents,[35,36] is a strong correlate of cognitive deficits in AD patients.[37,38] In *App*$^{NL-G-F}$ mice, prior work described synaptic impairment starting at 3–4 months,[39] which is in line with our results of a significant difference between *App*$^{NL-G-F}$ and WT mice at 7 months (Fig. 2B and Supplementary Fig. 1A). Synaptic loss has also been observed in App transgenic mouse models independent of Aβ plaque formation,[40] suggesting direct or indirect toxicities of Aβ oligomers. Aβ oligomers have been shown to bind specifically with stronger interactions to excitatory neurons.[41] Aβ oligomers can activate microglia to phagocytize synapses via complement activation[42] and may be directly neurotoxic.[43] Here we have also provided further evidence that aggregation-prone Aβ oligomers lead to pro-inflammatory activation of microglia, which release neurotoxic cytokines, complement and reactive oxygen species.[33] Both are likely to contribute to neuronal and cognitive dysfunction in the *App*$^{NL-G-F}$ model.[44,45] Additionally, we made the incidental observation that Aβ pathology was associated with increased expression of CD163 in both microglia and astrocytes. Phenotypic transition of microglia into astrocyte-like cells was reported previously in study of brain injury and chronic neurodegeneration in a rodent model.[27,46]

A strength of our study is that we have used two Aβ models based on a common C57BL/6 genetic background expressing APP under the control of the endogenous mouse App promoter, which facilitated their comparison. However, a limitation is that structural and conformational differences in the generated Aβ peptides can only be inferred, although there are undoubtedly differences. We also performed only a limited analysis of glial phenotypes based on classical immunohistological markers. This was particularly limited for astrocytes, the molecular phenotypes of which need to be described comprehensively in future work. The reliance on IMC for characterization of glial cells and markers, while powerful because it simultaneously allows multiple markers to be characterized, is limited by the lower sensitivity of IMC detection relative to that afforded by immunofluorescence and imaging in a single plane only. This is expected to artificially lower cell numbers sampled and could lead to a bias towards the activated glia with their enlarged cell bodies and thicker processes. Finally, we can only speculate about the probable mechanisms of neuronal injury as direct toxicity of Aβ species and indirect toxicity from inflammatory factors

For Review Only

could only be hypothesized.

In conclusion, our study has focused on the relationships between brain Aβ pathology, glial activation and neurodegeneration. Our results support evidence that the neurotoxicity of Aβ oligomers may be greater than that of the Aβ plaques, which have been the main focus of human clinical imaging biomarker studies.[47,48] They highlight how the Aβ oligomer response can depend on the conformation or aggregation state of the oligomers.[49] Therapeutic challenges for AD must be to better reduce concentrations of the most toxic oligomeric species,[50] limit the post-translational modifications leading to toxic conformations,[51] and reduce neurotoxic glial inflammatory responses without compromising glial contributions to the clearance of Aβ oligomers.[52]

Acknowledgments

The authors would like to thank Stephen Rothery at Imperial FILM Facilities for his assistance in fluorescence microscopy and data analysis. The authors would also like to acknowledge the technical staff from the UK Tissue Bank in Imperial College London, including Ildiko Farkas, Radhi Anand and Djordje Gveric.

Data Availability

The authors will make images and quantification available to researchers on reasonable request.

Funding

This research is supported by the Edmond J Safra Foundation and Lily Safra and an NIHR Senior Investigator Award to PMM. This work also is supported by the UK Dementia Research Institute, which received its funding from UK DRI Ltd., funded by the UK Medical Research Council, Alzheimer's Society and Alzheimer's Research UK. Infrastructure was supported by the National Institute for Health Research (NIHR) Biomedical Research Centre (BRC).

Competing Interests

PMM has received consultancy fees from Biogen, Nodthera, Sangamo and Roche. He has received honoraria or speakers' fees from Novartis and Biogen and has received research or educational funds from Bristol Meyers Squibb, Biogen, Novartis and GlaxoSmithKline. None of these interactions are related directly to this research, however. There are no competing

For Review Only

interests pertaining to this work.

Main Figures

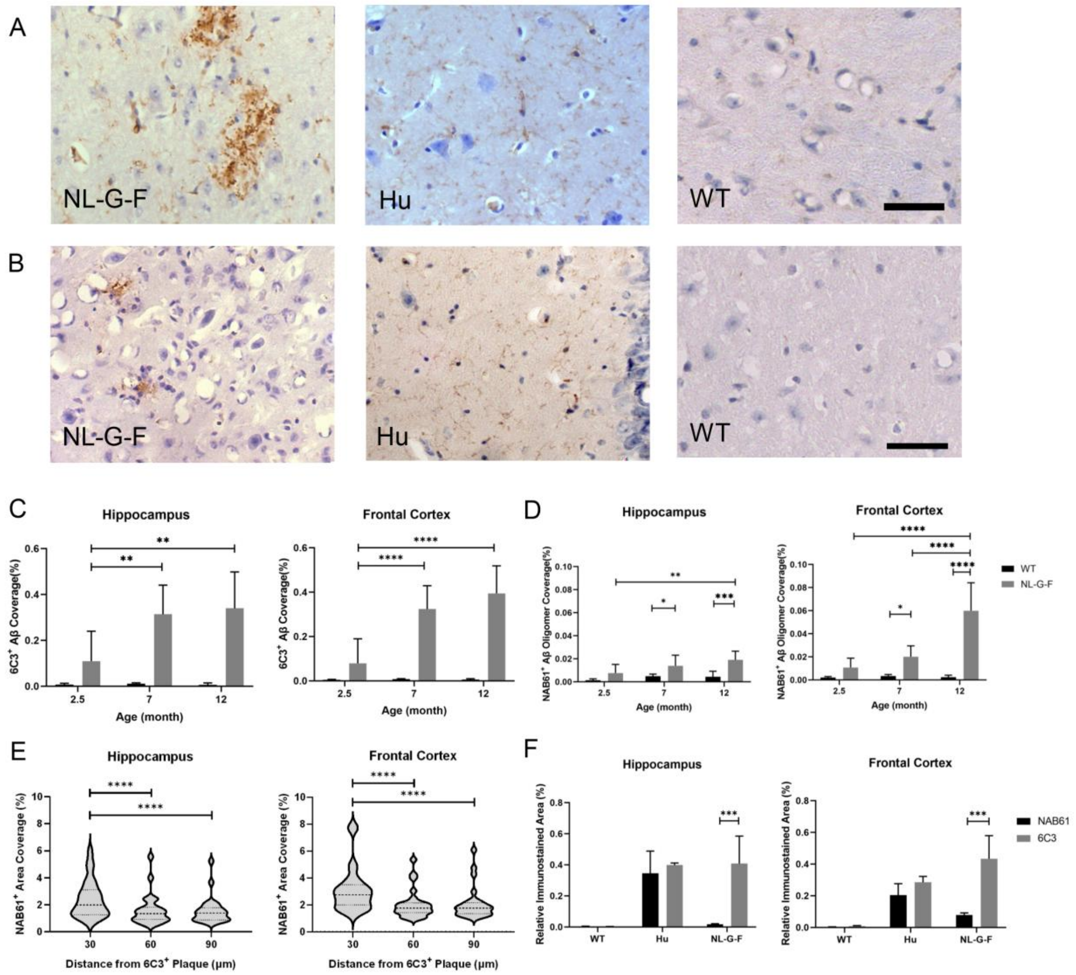

Figure 1. Comparison of Aβ pathology in the hippocampus and frontal cortex of *App*$^{\text{NL-G-F}}$, *App*^{hu} and WT mice. (A) Representative images of 6C3^{+} Aβ plaques and oligomers in 12-month-old mice. Scale bar = 50 μm. **(B)** Representative images of NAB61^{+} Aβ oligomers in 12-month-old mice. Scale bar = 50 μm. **(C, D)** Relative areas (%) occupied by 6C3^{+} Aβ plaque **(C)** and NAB61^{+} Aβ oligomer **(D)** of *App*$^{\text{NL-G-F}}$ and WT mice (n=6). **(E)** Sholl analysis using IMC of 6C3^{+} and NAB61^{+} Aβ staining in *App*$^{\text{NL-G-F}}$ mice at 12 months (n=3, one-way ANOVA). **(F)** Comparison between IHC signals for Aβ plaques and Aβ oligomers in 12-month-old mice (n=3). Columns represent the mean ± SD. Statistical analysis was performed using two-way ANOVA unless specifically labeled.

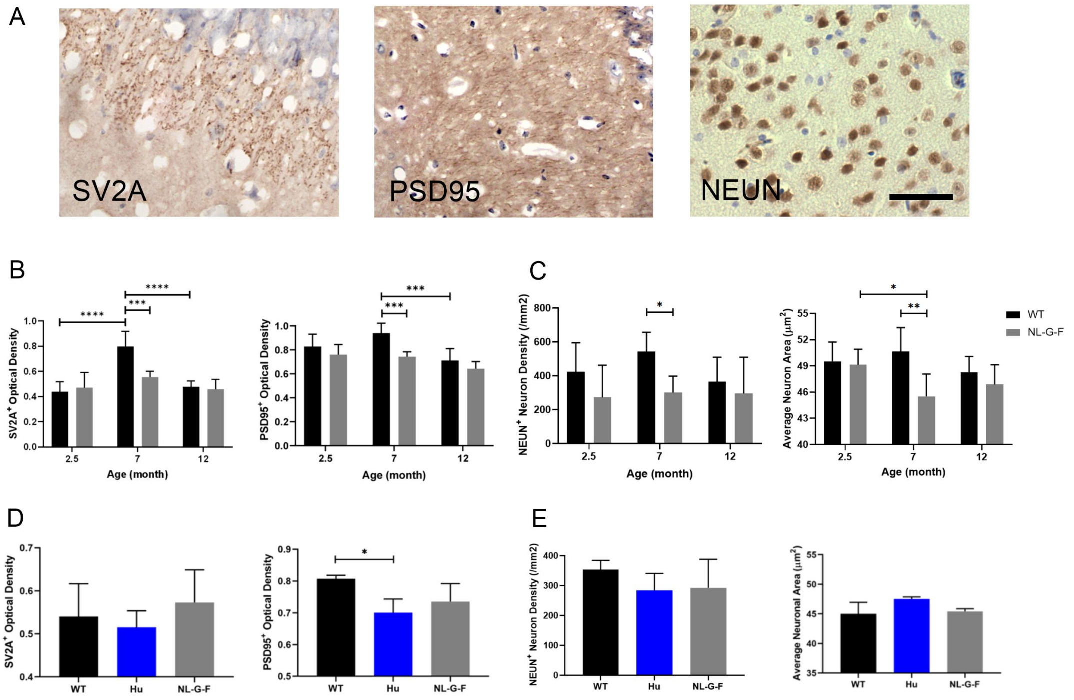

Figure 2. Comparison of neuronal and synaptic changes in the hippocampus and frontal cortex of *App*$^{NL\text{-}G\text{-}F}$, *App*^{hu} and WT mice. (A) Representative images of IHC staining for SV2A^{+} pre-synapses, PSD95^{+} post-synapses proteins and NEUN^{+} neurons. **(B)** Optical density of SV2A^{+} pre-synapses and PSD95^{+} post-synapses in the frontal cortex (n=6). **(C)** NEUN^{+} neuronal density and average neuronal area in the frontal cortex (n=6). **(D)** Optical density of pre-synaptic (SV2A^{+}) and post-synaptic (PSD95^{+}) signals in the hippocampus at 12 months (n=3). **(E)** NEUN^{+} neuronal density and average neuronal area in the hippocampus at 12 months (n=3). Columns represent the mean ± SD. Statistical analysis was performed using two-way ANOVA. Density is calculated as cell count/area. Scale bar = 50 μm.

For Review Only

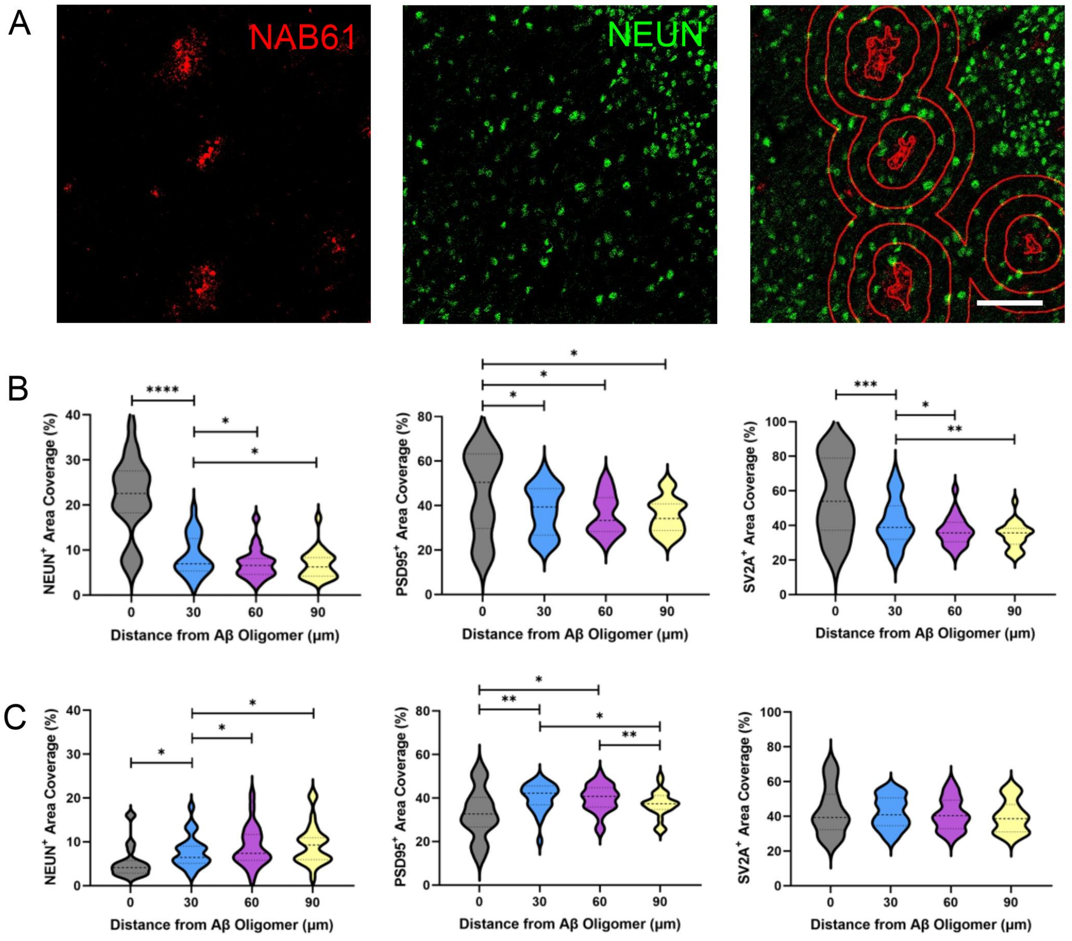

Figure 3. Sholl analysis of Aβ oligomers and neuronal markers in the hippocampus of *App*$^{NL-G-F}$ mice (n=3). (A) Representative IMC images with Sholl analysis of NAB61^{+} Aβ oligomers (red) and NEUN/PSD95/SV2A (green) in a 12-month-old mouse. **(B, C)** Sholl analysis of NAB61^{+} Aβ oligomers and NEUN/PSD95/SV2A in 2.5-month-old mice **(B)** and 12-month-old mice **(C)**. Columns represent the mean ± SD. Statistical analysis was performed using one-way ANOVA. Ring distance = 30 μm. Scale bar = 100 μm.

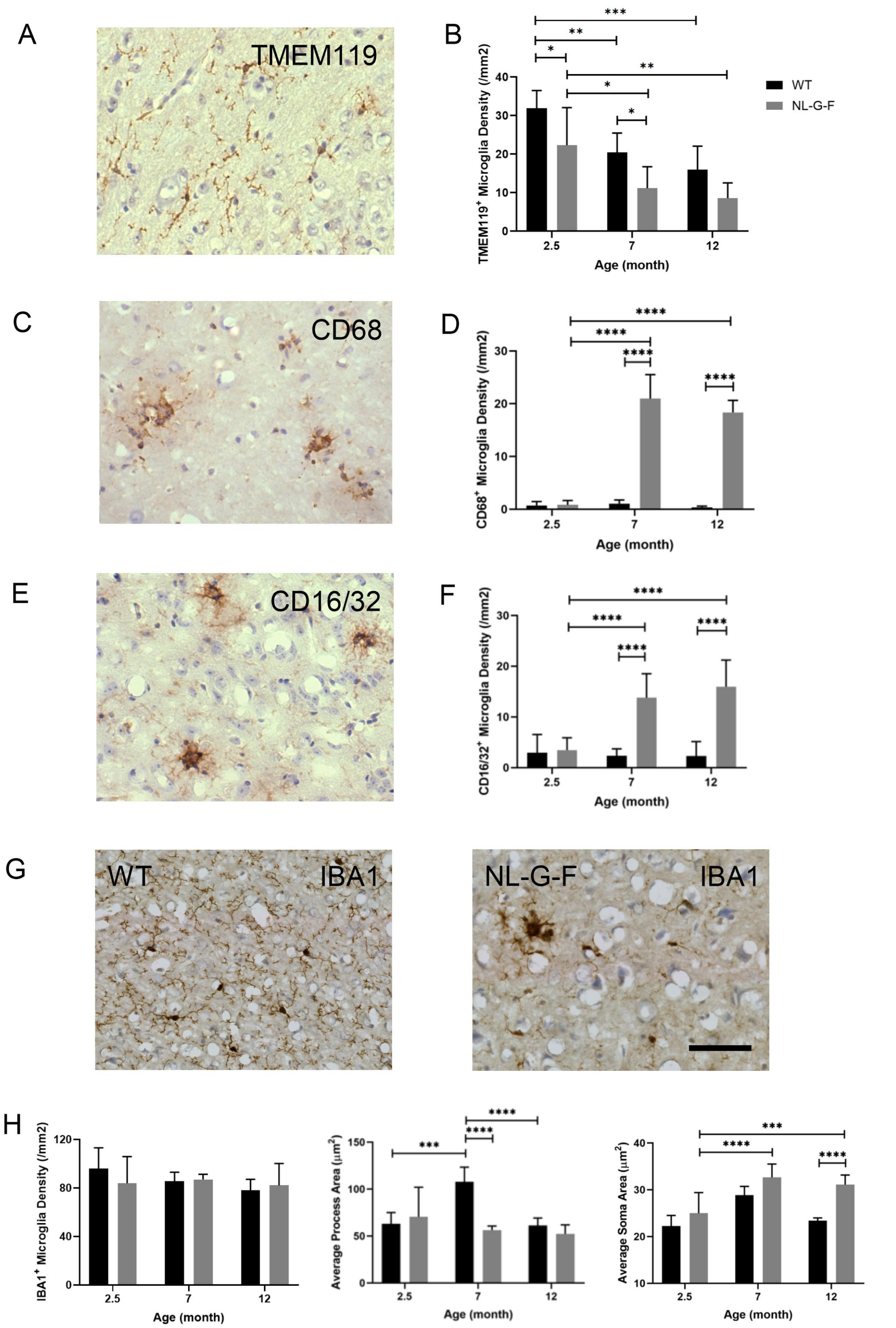

Figure 4. Age-related changes of microglia density and morphology in the frontal cortex of *App*$^{NL-G-F}$ and WT mice (n=6). (A) IHC staining images of TMEM119^{+} inactive microglia. **(B)** TMEM119^{+} microglia density.

(C) IHC staining images of CD68^{+} activated microglia. **(D)** CD68^{+} microglia density. **(E)** IHC staining images of CD16/32^{+} proinflammatory microglia. **(F)** CD16/32^{+} microglia density. **(G)** IHC staining images of IBA1^{+} microglia in 12-month-old mice. **(H)** IBA1^{+} microglia density and morphology. Columns represent the mean ± SD. Statistical analysis was performed using two-way ANOVA. Density is calculated as cell count/area. Scale bar = 50 μm.

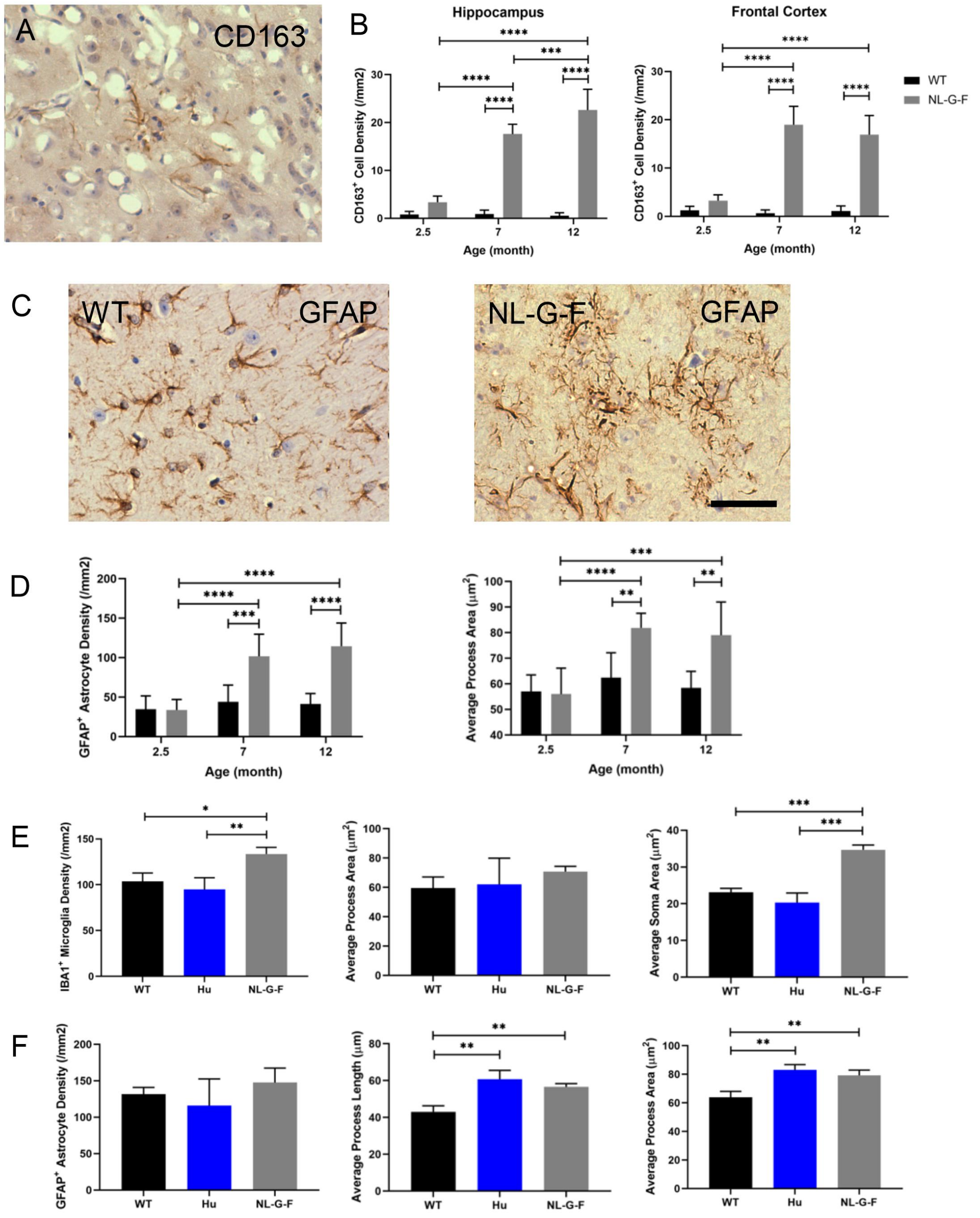

Figure 5. Age-related changes of glial density and morphology in *App*$^{NL\text{-}G\text{-}F}$, *App*^{hu} and WT mice. (A) IHC staining images of CD163^{+}

anti-inflammatory microglia and astrocytes. **(B)** CD163$^+$ cell density (n=6). **(C)** IHC staining images of GFAP$^+$ astrocytes in 12-month-old mice. **(D)** GFAP$^+$ astrocyte density and average process area in the frontal cortex (n=6). **(E)** IBA1$^+$ microglia density and morphology in the hippocampus of 12-month-old mice (n=3). **(F)** GFAP$^+$ astrocyte density and morphology in the hippocampus of 12-month-old mice (n=3). Columns represent the mean ± SD. Statistical analysis was performed using two-way ANOVA. Density is calculated as cell count/area. Scale bar = 50 µm.

For Review Only

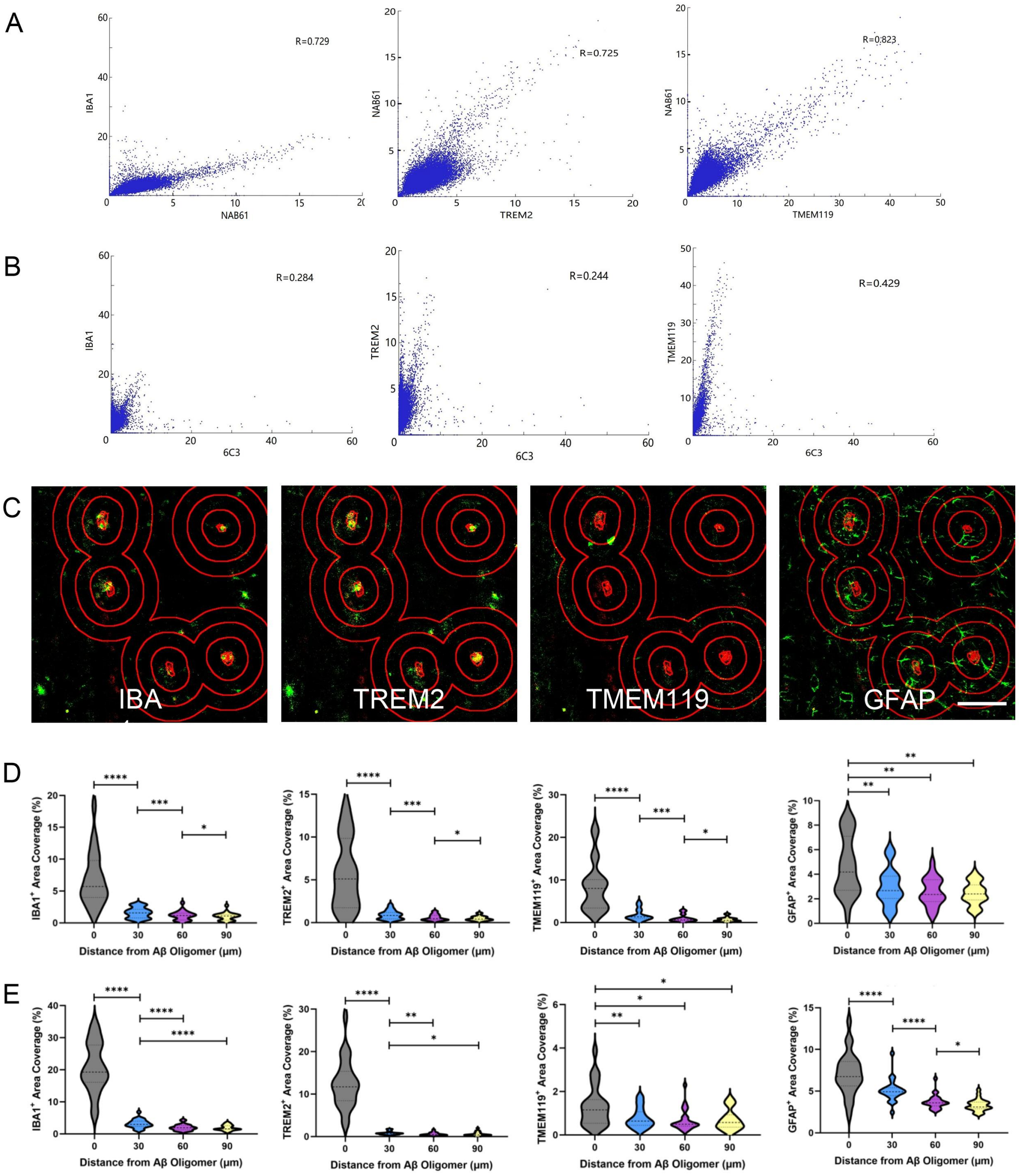

Figure 6. Sholl analysis of Aβ oligomers and glial markers in the hippocampus of *App*$^{NL\text{-}G\text{-}F}$ mice (n=3). (A) Correlation plots between NAB61$^+$ Aβ oligomers and microglia markers in 2.5-month-old mice. **(B)** Correlation plots between 6C3$^+$ Aβ plaques and microglia markers in 2.5-month-old mice. **(C)** Representative IMC images with Sholl analysis of NAB61$^+$ Aβ oligomers (red) and glial markers (green) in 12-month-old mice. **(D, E)** Sholl analysis of NAB61$^+$ Aβ oligomers and glial markers in 2.5-month-old mice **(D)** and 12-month-old mice **(E)**. Columns represent the mean ± SD, statistical analysis was performed using one-way ANOVA. Ring distance = 30 μm. Scale bar = 100 μm.

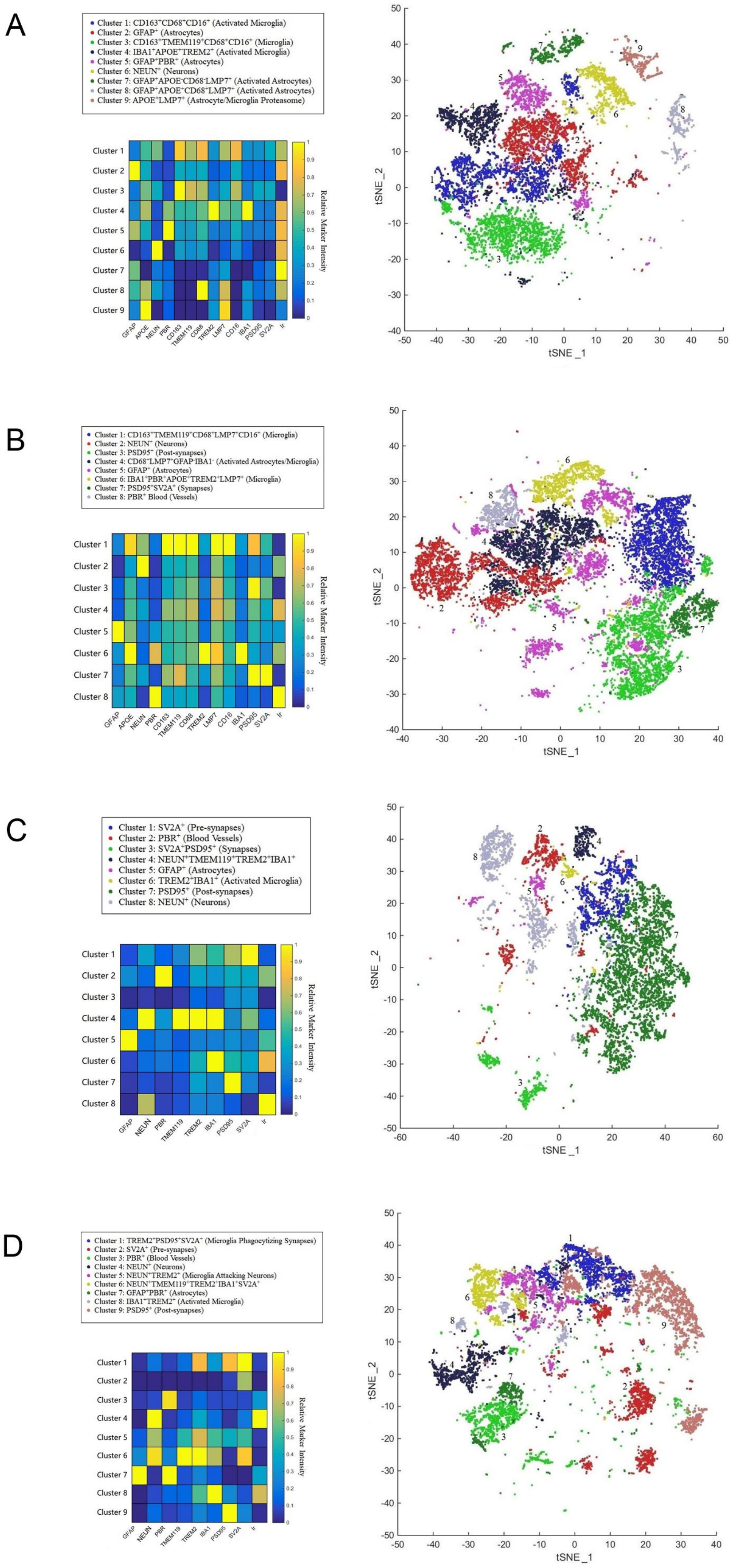
A
Cluster 1: CD163$^+$CD68$^+$CD16$^+$ (Activated Microglia)
Cluster 2: GFAP$^+$ (Astrocytes)
Cluster 3: CD163$^+$TMEM119$^+$CD68$^+$CD16$^+$ (Microglia)
Cluster 4: IBA1$^+$APOE$^+$TREM2$^+$ (Activated Microglia)
Cluster 5: GFAP$^+$PBR$^+$ (Astrocytes)
Cluster 6: NEUN$^+$ (Neurons)
Cluster 7: GFAP$^+$APOE$^-$CD68$^-$LMP7$^+$ (Activated Astrocytes)
Cluster 8: GFAP$^+$APOE$^+$CD68$^+$LMP7$^+$ (Activated Astrocytes)
Cluster 9: APOE$^+$LMP7$^+$ (Astrocyte/Microglia Proteasome)
Cluster 1
Cluster 2
Cluster 3
Cluster 4
Cluster 5
Cluster 6
Cluster 7
Cluster 8
Cluster 9
GFAP APOE NEUN PBR CD163 TMEM119 CD68 TREM2 LMP7 CD16 IBA1 PSD95 SV2A Ir
Relative Marker Intensity
tSNE_2
tSNE_1
B
Cluster 1: CD163$^+$TMEM119$^+$CD68$^+$LMP7$^+$CD16$^+$ (Microglia)
Cluster 2: NEUN$^+$ (Neurons)
Cluster 3: PSD95$^+$ (Post-synapses)
Cluster 4: CD68$^+$LMP7$^+$GFAP$^-$IBA1$^-$ (Activated Astrocytes/Microglia)
Cluster 5: GFAP$^+$ (Astrocytes)
Cluster 6: IBA1$^+$PBR$^+$APOE$^+$TREM2$^+$LMP7$^+$ (Microglia)
Cluster 7: PSD95$^+$SV2A$^+$ (Synapses)
Cluster 8: PBR$^+$ Blood (Vessels)
Cluster 1
Cluster 2
Cluster 3
Cluster 4
Cluster 5
Cluster 6
Cluster 7
Cluster 8
GFAP APOE NEUN PBR CD163 TMEM119 CD68 TREM2 LMP7 CD16 IBA1 PSD95 SV2A Ir
Relative Marker Intensity
tSNE_2
tSNE_1
C
Cluster 1: SV2A$^+$ (Pre-synapses)
Cluster 2: PBR$^+$ (Blood Vessels)
Cluster 3: SV2A$^+$PSD95$^+$ (Synapses)
Cluster 4: NEUN$^+$TMEM119$^+$TREM2$^+$IBA1$^+$
Cluster 5: GFAP$^+$ (Astrocytes)
Cluster 6: TREM2$^+$IBA1$^+$ (Activated Microglia)
Cluster 7: PSD95$^+$ (Post-synapses)
Cluster 8: NEUN$^+$ (Neurons)
Cluster 1
Cluster 2
Cluster 3
Cluster 4
Cluster 5
Cluster 6
Cluster 7
Cluster 8
GFAP NEUN PBR TMEM119 TREM2 IBA1 PSD95 SV2A Ir
Relative Marker Intensity
tSNE_2
tSNE_1
D
Cluster 1: TREM2$^+$PSD95$^+$SV2A$^+$ (Microglia Phagocytizing Synapses)
Cluster 2: SV2A$^+$ (Pre-synapses)
Cluster 3: PBR$^+$ (Blood Vessels)
Cluster 4: NEUN$^+$ (Neurons)
Cluster 5: NEUN$^+$TREM2$^+$ (Microglia Attacking Neurons)
Cluster 6: NEUN$^+$TMEM119$^+$TREM2$^+$IBA1$^+$SV2A$^+$
Cluster 7: GFAP$^+$PBR$^+$ (Astrocytes)
Cluster 8: IBA1$^+$TREM2$^+$ (Activated Microglia)
Cluster 9: PSD95$^+$ (Post-synapses)
Cluster 1
Cluster 2
Cluster 3
Cluster 4
Cluster 5
Cluster 6
Cluster 7
Cluster 8
Cluster 9
GFAP NEUN PBR TMEM119 TREM2 IBA1 PSD95 SV2A Ir
Relative Marker Intensity
tSNE_2
tSNE_1

Figure 7. Spatial IMC analysis of cellular markers in *App*$^{NL\text{-}G\text{-}F}$ mice (n=3). (A,B) Heatmap and phenograph clustering with t-distributed stochastic neighbor embedding (tSNE) in the hippocampus **(A)** and frontal cortex **(B)** of 12-month-old mice. **(C,D)** Heatmap and phenograph clustering with tSNE in hippocampus **(C)** and frontal cortex **(D)** of 2.5-month-old mice. Ir (intercalator) marks cell nuclei.

Supplemental Figure and Table Legends

Supplementary Figure 1. Comparison of neuronal and synaptic changes in the hippocampus and frontal cortex of *App*$^{NL\text{-}G\text{-}F}$, *App*^{hu} and WT mice. (A) Optical density of the pre-synaptic SV2A$^+$ and post-synaptic PSD95$^+$ signals in the hippocampus (n=6). **(B)** NEUN$^+$ neuronal density and average neuronal area in the hippocampus (n=6). **(C)** Optical density of the pre-synaptic SV2A$^+$ and post-synaptic PSD95$^+$ signals in the frontal cortex at 12 months (n=3). **(D)** NEUN$^+$ neuronal density and average neuronal area in the frontal cortex at 12 months (n=3). **(E)** Representative IMC images of 16 markers. Columns represent the mean ± SD, statistical analysis was performed using two-way ANOVA. Density is calculated as cell count/area. Scale bar = 100 µm.

Supplementary Figure 2. Sholl analysis of Aβ proteins and neuronal pathology in *App*$^{NL\text{-}G\text{-}F}$ mice (n=3). (A,B) Sholl analysis of NEUN$^+$ neurons and Aβ oligomers **(A)** or Aβ plaques **(B)** in 2.5-month-old mice. **(C,D)** Sholl analysis between NeuN$^+$ neurons and Aβ oligomers **(C)** or Aβ plaques **(D)** in 12-month-old mice. **(E, F)** Sholl analysis of PSD95$^+$ synapses and Aβ oligomers **(E)** or Aβ plaques **(F)** in 2.5-month-old mice. **(G, H)** Sholl analysis of PSD95$^+$ synapses and Aβ oligomers **(G)** or Aβ plaques **(H)** in 12-month-old mice. **(I, J)** Sholl analysis of SV2A$^+$ synapses and Aβ oligomers **(I)** or Aβ plaques **(J)** in 2.5-month-old mice. **(K, L)** Sholl analysis of SV2A$^+$ synapses and Aβ oligomers **(K)** or Aβ plaques **(L)** in 12-month-old mice. Columns represent the mean ± SD, statistical analysis was performed using one-way ANOVA.

Supplementary Figure 3. Age-related changes of glial density and morphology in *App*$^{NL\text{-}G\text{-}F}$, *App*^{hu} and WT mice. (A) Phenotypic microglia density in the hippocampus (n=6). **(B)** IBA1$^+$ microglia density and morphology in the hippocampus (n=6). **(C)** GFAP$^+$ astrocyte density and morphology in the hippocampus (n=6). **(D, E)** Correlation plots between PBR$^+$ and IBA1$^+$ microglia **(D)** or GFAP$^+$ astrocytes **(E)** in the frontal cortex of *App*$^{NL\text{-}G\text{-}F}$ mice (n=3). **(F)** IBA1$^+$ microglia density and morphology in the frontal cortex of 12-month-old mice (n=3). **(G)** GFAP$^+$ astrocyte density and morphology in the frontal cortex of 12-month-old mice (n=3). Columns represent the mean ± SD. Statistical analysis was performed using two-way

For Review Only

ANOVA. Density is calculated as cell count/area.

Supplementary Figure 4. Immunofluorescence staining reveals distinct cell types with different patterns of phenotypic marker expression. (A) Double staining images of IBA1 (red) and TMEM119 (green). **(B)** Double staining images of CD68 (red) and IBA1 (green). **(C)** Double staining images of CD16/32 (red) and IBA1 (green). **(D)** Double staining images of GFAP (red) and CD163 (green). **(E)** Double staining images of IBA1 (red) and CD163 (green). **(F)** Double staining images of GFAP (red) and PBR (green). **(G)** Double staining images of IBA1 (red) and PBR (green). Scale bar = 100 µm.

Supplementary Figure 5. Age-related changes of PBR^{+} glial density in *App*$^{NL-G-F}$, *App*^{hu} and WT mice. (A) IHC staining images of PBR^{+} proinflammatory microglia and astrocytes. **(B)** Age-related changes of PBR^{+} cell density in *App*$^{NL-G-F}$ and WT mice (n=6). **(C)** PBR^{+} cell density in 12-month-old *App*$^{NL-G-F}$, *App*^{hu} and WT mice. Columns represent the mean ± SD. Statistical analysis was performed using two-way ANOVA. Density is calculated as cell count/area. Scale bar = 50 µm.

Supplementary Figure 6. Comparison of different glial phenotypes in the hippocampus and frontal cortex of *App*$^{NL-G-F}$, *App*^{hu} and WT mice (n=3). (A) IHC staining images of TMEM119^{+} inactive microglia. **(B)** TMEM119^{+} microglia density. **(C)** IHC staining images of CD68^{+} activated microglia. **(D)** CD68^{+} microglia density. **(E)** IHC staining images of CD16/32^{+} proinflammatory microglia. **(F)** CD16/32^{+} microglia density. **(G)** IHC staining images of CD163^{+} anti-inflammatory astrocytes. **(H)** CD163^{+} astrocyte density. Columns represent the mean ± SD. Statistical analysis was performed using one-way ANOVA. Density is calculated as cell count/area. Scale bar = 50 µm.

Supplementary Figure 7. Correlation between microglia markers and Aβ in 12-month-old *App*$^{NL-G-F}$ mice (n=3). (A, B) Correlation plots between NAB61^{+} Aβ oligomers and all microglia markers in the frontal cortex **(A)** and hippocampus **(B)**. **(C, D)** Correlation plots between 6C3^{+} Aβ plaques and all microglia markers in the frontal cortex **(C)** and hippocampus **(D)**.

Supplementary Figure 8. Sholl analysis of Aβ pathology and microglia markers in *App*$^{NL-G-F}$ mice of different ages (n=3). (A, B) Sholl analysis of IBA1^{+} microglia and Aβ oligomers **(A)** or Aβ plaques **(B)** in 2.5-month-old mice. **(C, D)** Sholl analysis of TREM2^{+} microglia and Aβ oligomers **(C)** or Aβ plaques **(D)** in 2.5-month-old mice. **(E, F)** Sholl analysis of TMEM119^{+} microglia and Aβ oligomers **(E)** or Aβ plaques **(F)** in 2.5-month-old mice.

For Review Only

(G, H) Sholl analysis of IBA1$^+$ microglia and Aβ oligomers **(G)** or Aβ plaques **(H)** in 12-month-old mice. **(I, J)** Sholl analysis of TREM2$^+$ microglia and Aβ oligomers **(I)** or Aβ plaques **(J)** in 12-month-old mice. **(K, L)** Sholl analysis of TMEM119$^+$ microglia and Aβ oligomers **(K)** or Aβ plaques **(L)** in 12-month-old mice. Columns represent the mean ± SD, statistical analysis was performed using one-way ANOVA.

Supplementary Figure 9. Representative IMC staining images of glial phenotypic clusters. (A) GFAP$^+$APOE$^-$CD68$^-$LMP7$^+$ activated astrocytes. **(B)** GFAP$^+$APOE$^+$CD68$^+$LMP7$^+$ activated astrocytes. **(C)** CD68$^+$LMP7$^+$GFAP$^-$IBA1$^-$ activated glial cells. **(D)** IBA1$^+$PBR$^+$APOE$^+$TREM2$^+$LMP7$^+$ activated microglia. **(E)** NEUN$^+$TREM2$^+$ cluster. **(F)** TREM2$^+$PSD95$^+$SV2A$^+$ cluster. Scale bar = 50 μm.

Supplementary Table 1. PCR reaction setup.

Supplementary Table 2. PCR reaction thermocycling conditions.

Supplementary Table 3. Primary antibody selection for IHC staining.

Supplementary Table 4. Primary antibody selection for IF staining.

Supplementary Table 5. Secondary antibody selection for IF staining.

Supplementary Table 6. Primary antibody cocktail for IMC staining.

Supplementary Table 7. Colocalization area coverage surrounding Aβ plaques or oligomers with Sholl analysis.

Supplementary Table 8. R value summary of correlation plots.

Supplementary File: Code for Sholl analysis in ImageJ.

References

1. Nordengen K, Kirsebom B-E, Henjum K, et al. Glial activation and inflammation along the Alzheimer's disease continuum. *Journal of neuroinflammation.* 2019;16(1):1-13.
2. Bartels T, De Schepper S, Hong S. Microglia modulate neurodegeneration in Alzheimer's and Parkinson's diseases. *Science.* 2020;370(6512):66-69.
3. Liddelow SA, Barres BA. Reactive astrocytes: production, function, and therapeutic potential. *Immunity.* 2017;46(6):957-967.

For Review Only

4. Heneka MT, Rodríguez JJ, Verkhratsky A. Neuroglia in neurodegeneration. *Brain research reviews.* 2010;63(1-2):189-211.
5. Carter SF, Herholz K, Rosa-Neto P, Pellerin L, Nordberg A, Zimmer ER. Astrocyte biomarkers in Alzheimer's disease. *Trends in molecular medicine.* 2019;25(2):77-95.
6. Sierksma A, Lu A, Mancuso R, et al. Novel Alzheimer risk genes determine the microglia response to amyloid-β but not to TAU pathology. *EMBO Molecular Medicine.* 2020;12(3):e10606.
7. Calsolaro V, Matthews PM, Donat CK, et al. Astrocyte reactivity with late-onset cognitive impairment assessed in vivo using 11C-BU99008 PET and its relationship with amyloid load. *Molecular psychiatry.* 2021:1-8.
8. Chen G-f, Xu T-h, Yan Y, et al. Amyloid beta: structure, biology and structure-based therapeutic development. *Acta Pharmacologica Sinica.* 2017;38(9):1205-1235.
9. Brown MR, Radford SE, Hewitt EW. Modulation of β-amyloid fibril formation in Alzheimer's disease by microglia and infection. *Frontiers in Molecular Neuroscience.* 2020;13:228.
10. Santin MD, Vandenberghe ME, Herard A-S, et al. In vivo detection of amyloid plaques by gadolinium-stained MRI can be used to demonstrate the efficacy of an anti-amyloid immunotherapy. *Frontiers in aging neuroscience.* 2016;8:55.
11. Szała-Mendyk B, Molski A. Diverse Aggregation Kinetics Predicted by a Coarse-Grained Peptide Model. *The Journal of Physical Chemistry B.* 2021;125(28):7587-7597.
12. De Felice FG, Vieira MN, Saraiva LM, et al. Targeting the neurotoxic species in Alzheimer's disease: inhibitors of Aβ oligomerization. *The FASEB Journal.* 2004;18(12):1366-1372.
13. Mucke L, Selkoe DJ. Neurotoxicity of amyloid β-protein: synaptic and network dysfunction. *Cold Spring Harbor perspectives in medicine.* 2012;2(7):a006338.
14. Lu R-M, Hwang Y-C, Liu I-J, et al. Development of therapeutic antibodies for the treatment of diseases. *Journal of biomedical science.* 2020;27(1):1-30.
15. Hong S, Beja-Glasser VF, Nfonoyim BM, et al. Complement and microglia mediate early synapse loss in Alzheimer mouse models. *Science.* 2016;352(6286):712-716.
16. Saito T, Matsuba Y, Mihira N, et al. Single App knock-in mouse models of Alzheimer's disease. *Nature neuroscience.* 2014;17(5):661-663.
17. Lu M, Williamson N, Mishra A, et al. Structural progression of amyloid-β Arctic mutant aggregation in cells revealed by multiparametric imaging. *Journal of Biological Chemistry.* 2019;294(5):1478-1487.

For Review Only

18. Sasaguri H, Nilsson P, Hashimoto S, et al. APP mouse models for Alzheimer's disease preclinical studies. *The EMBO journal.* 2017;36(17):2473-2487.
19. Serneels L, T'Syen D, Perez-Benito L, Theys T, Holt MG, De Strooper B. Modeling the β-secretase cleavage site and humanizing amyloid-beta precursor protein in rat and mouse to study Alzheimer's disease. *Molecular Neurodegeneration.* 2020;15(1):1-11.
20. Sampath D, Sathyanesan M, Newton SS. Cognitive dysfunction in major depression and Alzheimer's disease is associated with hippocampal–prefrontal cortex dysconnectivity. *Neuropsychiatric Disease and Treatment.* 2017;13:1509.
21. Graham WV, Bonito-Oliva A, Sakmar TP. Update on Alzheimer's disease therapy and prevention strategies. *Annual review of medicine.* 2017;68:413-430.
22. Shah D, Latif-Hernandez A, De Strooper B, et al. Spatial reversal learning defect coincides with hypersynchronous telencephalic BOLD functional connectivity in APP NL-F/NL-F knock-in mice. *Scientific reports.* 2018;8(1):1-11.
23. Lee EB, Leng L, Zhang B, et al. Targeting Abeta oligomers by passive immunization with a conformation selective monoclonal antibody improves learning and memory in APP transgenic mice. *J Biol Chem.* 2005;281:4292-4299.
24. Woerman AL, Oehler A, Kazmi SA, et al. Multiple system atrophy prions retain strain specificity after serial propagation in two different Tg (SNCA* A53T) mouse lines. *Acta neuropathologica.* 2019;137(3):437-454.
25. Baharlou H, Canete NP, Cunningham AL, Harman AN, Patrick E. Mass cytometry imaging for the study of human diseases—applications and data analysis strategies. *Frontiers in immunology.* 2019;10:2657.
26. Cotman CW, Su JH. Mechanisms of neuronal death in Alzheimer's disease. *Brain Pathology.* 1996;6(4):493-506.
27. Trias E, Díaz-Amarilla P, Olivera-Bravo S, et al. Phenotypic transition of microglia into astrocyte-like cells associated with disease onset in a model of inherited ALS. *Frontiers in cellular neuroscience.* 2013;7:274.
28. Keren-Shaul H, Spinrad A, Weiner A, et al. A Unique Microglia Type Associated with Restricting Development of Alzheimer's Disease. *Cell.* 2017;169(7):1276-1290 e1217.
29. Sideris DI, Danial JS, Emin D, et al. Soluble amyloid beta-containing aggregates are present throughout the brain at early stages of Alzheimer's disease. *Brain communications.* 2021;3(3):fcab147.
30. Morten MJ, Sirvio L, Rupawala H, et al. Quantitative super-resolution imaging of pathological aggregates reveals distinct

For Review Only

toxicity profiles in different synucleinopathies. *Proceedings of the National Academy of Sciences.* 2022;119(41):e2205591119.
31. Venkataraman AV, Mansur A, Rizzo G, et al. Widespread cell stress and mitochondrial dysfunction occur in patients with early Alzheimer's disease. *Science translational medicine.* 2022;14(658):eabk1051.
32. Wyssenbach A, Quintela T, Llavero F, Zugaza JL, Matute C, Alberdi E. Amyloid β-induced astrogliosis is mediated by β1-integrin via NADPH oxidase 2 in Alzheimer's disease. *Aging Cell.* 2016;15(6):1140-1152.
33. Diniz LP, Tortelli V, Matias I, et al. Astrocyte transforming growth factor beta 1 protects synapses against Aβ oligomers in Alzheimer's disease model. *Journal of Neuroscience.* 2017;37(28):6797-6809.
34. Smith AM, Davey K, Tsartsalis S, et al. Diverse human astrocyte and microglial transcriptional responses to Alzheimer's pathology. *Acta neuropathologica.* 2022;143(1):75-91.
35. Tucsek Z, Noa Valcarcel-Ares M, Tarantini S, et al. Hypertension-induced synapse loss and impairment in synaptic plasticity in the mouse hippocampus mimics the aging phenotype: implications for the pathogenesis of vascular cognitive impairment. *Geroscience.* 2017;39(4):385-406.
36. Petralia RS, Mattson MP, Yao PJ. Communication breakdown: the impact of ageing on synapse structure. *Ageing research reviews.* 2014;14:31-42.
37. Subramanian J, Savage JC, Tremblay M-È. Synaptic loss in Alzheimer's disease: mechanistic insights provided by two-photon in vivo imaging of transgenic mouse models. *Frontiers in Cellular Neuroscience.* 2020;14:445.
38. De Wilde MC, Overk CR, Sijben JW, Masliah E. Meta-analysis of synaptic pathology in Alzheimer's disease reveals selective molecular vesicular machinery vulnerability. *Alzheimer's & Dementia.* 2016;12(6):633-644.
39. Latif-Hernandez A, Sabanov V, Ahmed T, et al. The two faces of synaptic failure in App NL-GF knock-in mice. *Alzheimer's Research & Therapy.* 2020;12(1):1-15.
40. Mucke L, Masliah E, Yu G-Q, et al. High-level neuronal expression of Aβ1–42 in wild-type human amyloid protein precursor transgenic mice: synaptotoxicity without plaque formation. *Journal of Neuroscience.* 2000;20(11):4050-4058.
41. Lacor PN, Buniel MC, Furlow PW, et al. Aβ oligomer-induced aberrations in synapse composition, shape, and density provide a molecular basis for loss of connectivity in Alzheimer's disease. *Journal of Neuroscience.* 2007;27(4):796-807.
42. Lian H, Litvinchuk A, Chiang AC-A, Aithmitti N, Jankowsky JL,

For Review Only

Zheng H. Astrocyte-microglia cross talk through complement activation modulates amyloid pathology in mouse models of Alzheimer's disease. *Journal of Neuroscience.* 2016;36(2):577-589.
43. Shankar G, Li S, Mehta T, et al. Soluble amyloid β-protein dimers isolated directly from Alzheimer disease patients potently impair synaptic plasticity and memory. *Nat Med.* 2008;14:837-842.
44. Mehla J, Lacoursiere SG, Lapointe V, et al. Age-dependent behavioral and biochemical characterization of single APP knock-in mouse (APPNL-GF/NL-GF) model of Alzheimer's disease. *Neurobiology of aging.* 2019;75:25-37.
45. Sakakibara Y, Sekiya M, Saito T, Saido TC, Iijima KM. Amyloid-β plaque formation and reactive gliosis are required for induction of cognitive deficits in App knock-in mouse models of Alzheimer's disease. *BMC neuroscience.* 2019;20(1):1-14.
46. Wilhelmsson U, Andersson D, De Pablo Y, et al. Injury leads to the appearance of cells with characteristics of both microglia and astrocytes in mouse and human brain. *Cerebral Cortex.* 2017;27(6):3360-3377.
47. Hardy JA, Higgins GA. Alzheimer's disease: the amyloid cascade hypothesis. *Science.* 1992;256(5054):184-185.
48. Nelson PT, Alafuzoff I, Bigio EH, et al. Correlation of Alzheimer disease neuropathologic changes with cognitive status: a review of the literature. *Journal of Neuropathology & Experimental Neurology.* 2012;71(5):362-381.
49. Stine WB, Jungbauer L, Yu C, LaDu MJ. Preparing synthetic Aβ in different aggregation states. *Alzheimer's Disease and Frontotemporal Dementia: Methods and Protocols.* 2011:13-32.
50. Kreiser RP, Wright AK, Block NR, et al. Therapeutic strategies to reduce the toxicity of misfolded protein oligomers. *International Journal of Molecular Sciences.* 2020;21(22):8651.
51. Grochowska KM, Yuanxiang P, Bär J, et al. Posttranslational modification impact on the mechanism by which amyloid-β induces synaptic dysfunction. *EMBO reports.* 2017;18(6):962-981.
52. Nordengen K, Kirsebom B-E, Henjum K, et al. Glial activation and inflammation along the Alzheimer's disease continuum. *Journal of neuroinflammation.* 2019;16:1-13.

For Review Only

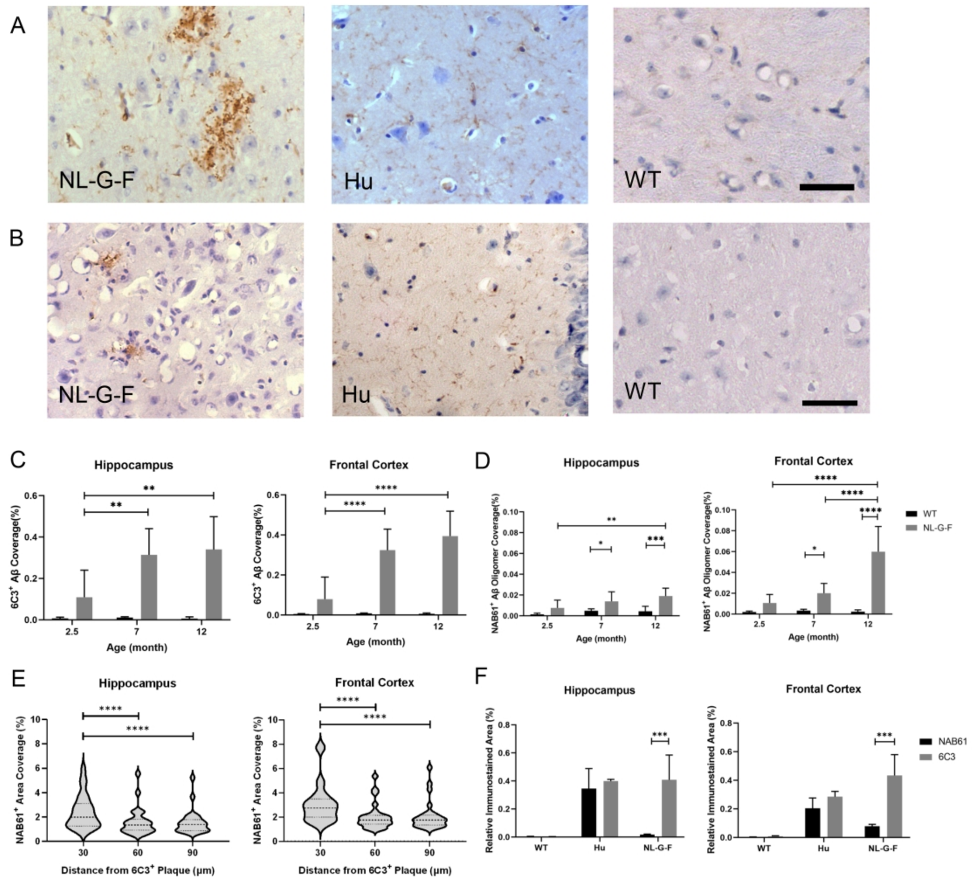

Figure 1. Comparison of Aβ pathology in the hippocampus and frontal cortex of AppNL-G-F, Apphu and WT mice. (A) Representative images of 6C3+ Aβ plaques and oligomers in 12-month-old mice. Scale bar = 50 μm. (B) Representative images of NAB61+ Aβ oligomers in 12-month-old mice. Scale bar = 50 μm. (C, D) Relative areas (%) occupied by 6C3+ Aβ plaque (C) and NAB61+ Aβ oligomer (D) of AppNL-G-F and WT mice (n=6). (E) Sholl analysis using IMC of 6C3+ and NAB61+ Aβ staining in AppNL-G-F mice at 12 months (n=3, one-way ANOVA). (F) Comparison between IHC signals for Aβ plaques and Aβ oligomers in 12-month-old mice (n=3). Columns represent the mean ± SD. Statistical analysis was performed using two-way ANOVA unless specifically labeled.

213x194mm (300 x 300 DPI)

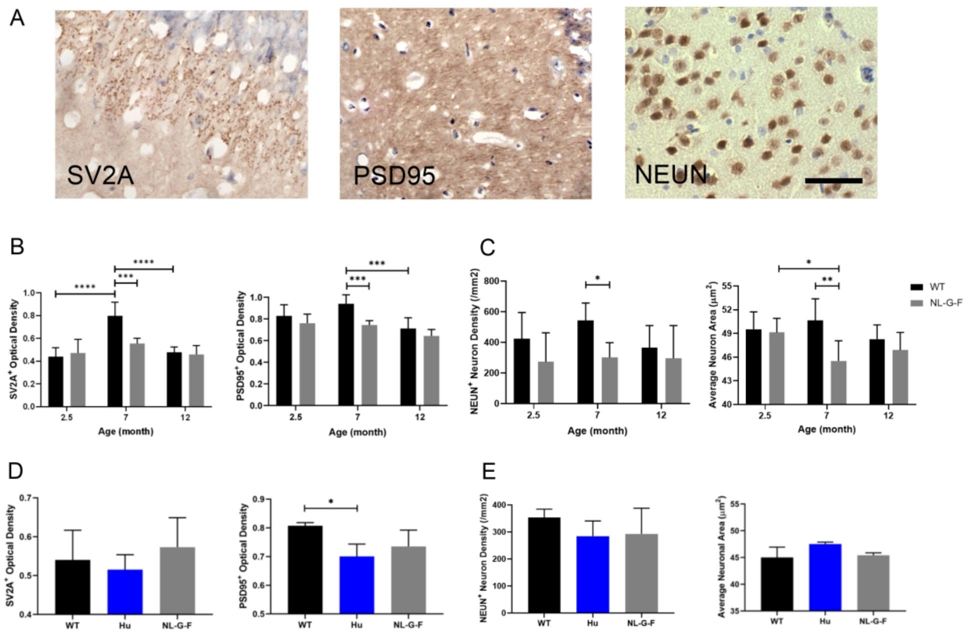

Figure 2. Comparison of neuronal and synaptic changes in the hippocampus and frontal cortex of AppNL-G-F, Apphu and WT mice. (A) Representative images of IHC staining for SV2A+ pre-synapses, PSD95+ post-synapses proteins and NEUN+ neurons. (B) Optical density of SV2A+ pre-synapses and PSD95+ post-synapses in the frontal cortex (n=6). (C) NEUN+ neuronal density and average neuronal area in the frontal cortex (n=6). (D) Optical density of pre-synaptic (SV2A+) and post-synaptic (PSD95+) signals in the hippocampus at 12 months (n=3). (E) NEUN+ neuronal density and average neuronal area in the hippocampus at 12 months (n=3). Columns represent the mean ± SD. Statistical analysis was performed using two-way ANOVA. Density is calculated as cell count/area. Scale bar = 50 µm.

217x143mm (300 x 300 DPI)

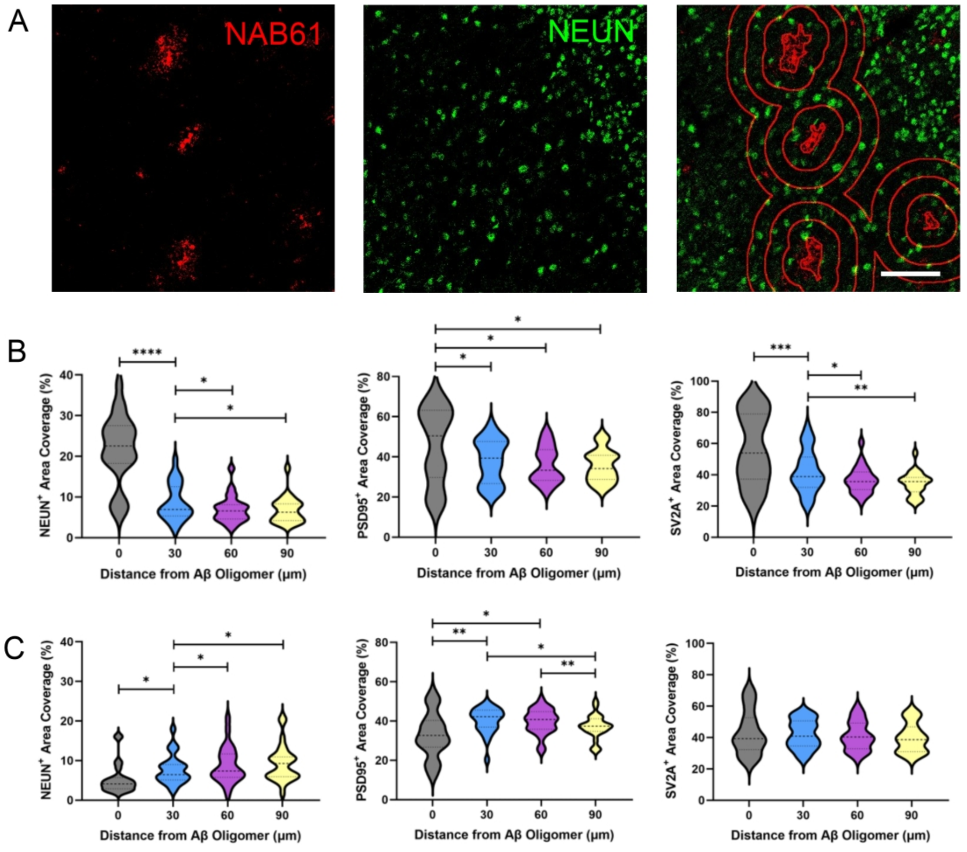

Figure 3. Sholl analysis of Aβ oligomers and neuronal markers in the hippocampus of AppNL-G-F mice (n=3). (A) Representative IMC images with Sholl analysis of NAB61+ Aβ oligomers (red) and NEUN/PSD95/SV2A (green) in a 12-month-old mouse. (B, C) Sholl analysis of NAB61+ Aβ oligomers and NEUN/PSD95/SV2A in 2.5-month-old mice (B) and 12-month-old mice (C). Columns represent the mean ± SD. Statistical analysis was performed using one-way ANOVA. Ring distance = 30 μm. Scale bar = 100 μm.

181x161mm (300 x 300 DPI)

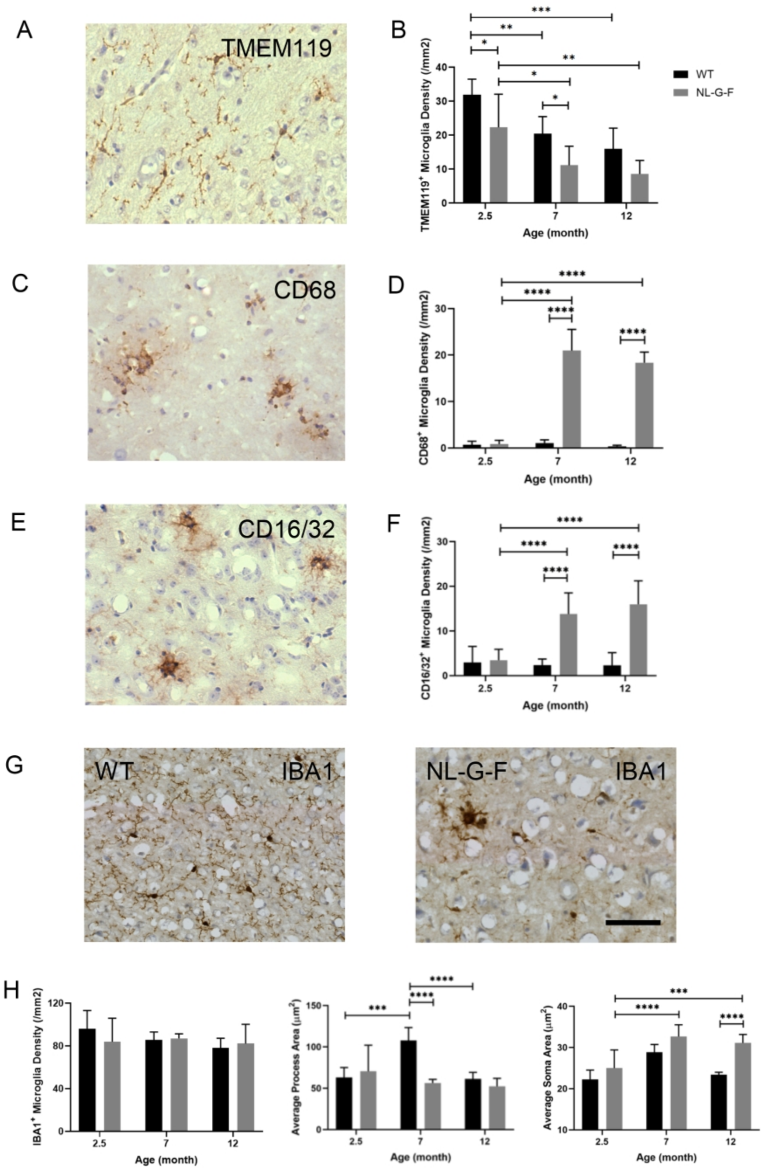

Figure 4. Age-related changes of microglia density and morphology in the frontal cortex of AppNL-G-F and WT mice (n=6). (A) IHC staining images of TMEM119+ inactive microglia. (B) TMEM119+ microglia density. (C) IHC staining images of CD68+ activated microglia. (D) CD68+ microglia density. (E) IHC staining images of CD16/32+ proinflammatory microglia. (F) CD16/32+ microglia density. (G) IHC staining images of IBA1+ microglia in 12-month-old mice. (H) IBA1+ microglia density and morphology. Columns represent the mean ± SD. Statistical analysis was performed using two-way ANOVA. Density is calculated as cell count/area. Scale bar = 50 μm.

181x274mm (300 x 300 DPI)

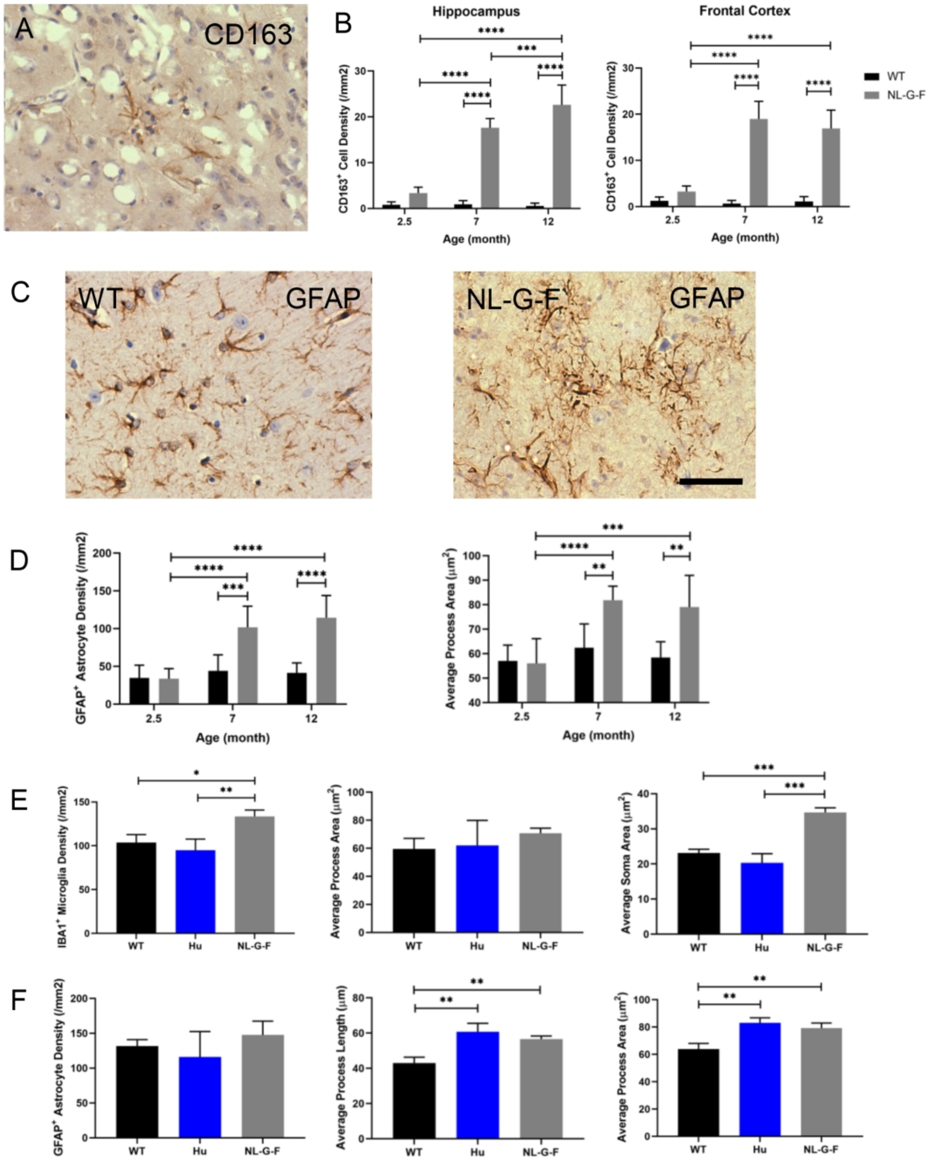

Figure 5. Age-related changes of glial density and morphology in AppNL-G-F, Apphu and WT mice. (A) IHC staining images of CD163+ anti-inflammatory microglia and astrocytes. (B) CD163+ cell density (n=6). (C) IHC staining images of GFAP+ astrocytes in 12-month-old mice. (D) GFAP+ astrocyte density and average process area in the frontal cortex (n=6). (E) IBA1+ microglia density and morphology in the hippocampus of 12-month-old mice (n=3). (F) GFAP+ astrocyte density and morphology in the hippocampus of 12-month-old mice (n=3). Columns represent the mean ± SD. Statistical analysis was performed using two-way ANOVA. Density is calculated as cell count/area. Scale bar = 50 μm.

190x237mm (300 x 300 DPI)

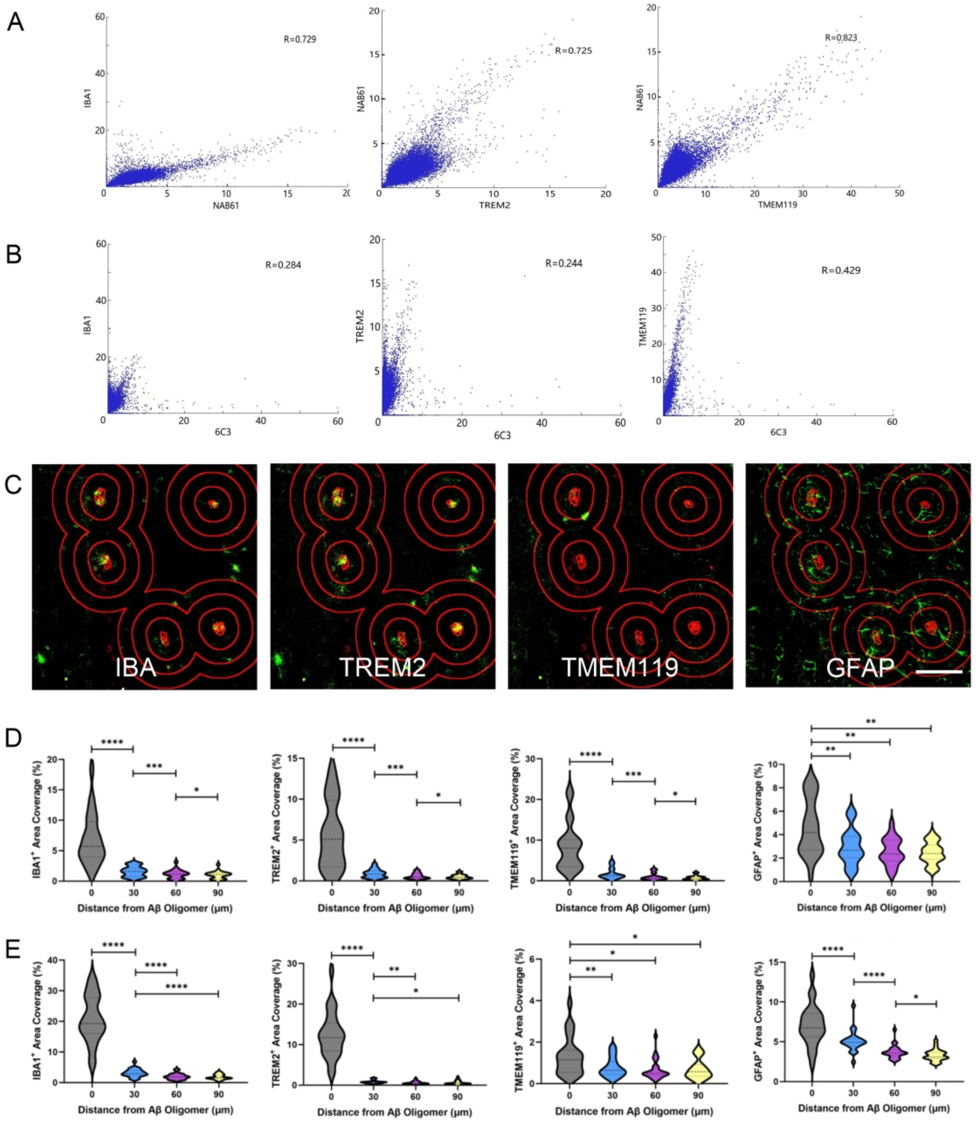

Figure 6. Sholl analysis of Aβ oligomers and glial markers in the hippocampus of AppNL-G-F mice (n=3). (A) Correlation plots between NAB61+ Aβ oligomers and microglia markers in 2.5-month-old mice. (B) Correlation plots between 6C3+ Aβ plaques and microglia markers in 2.5-month-old mice. (C) Representative IMC images with Sholl analysis of NAB61+ Aβ oligomers (red) and glial markers (green) in 12-month-old mice. (D, E) Sholl analysis of NAB61+ Aβ oligomers and glial markers in 2.5-month-old mice (D) and 12-month-old mice (E). Columns represent the mean ± SD, statistical analysis was performed using one-way ANOVA. Ring distance = 30 μm. Scale bar = 100 μm.

217x251mm (300 x 300 DPI)

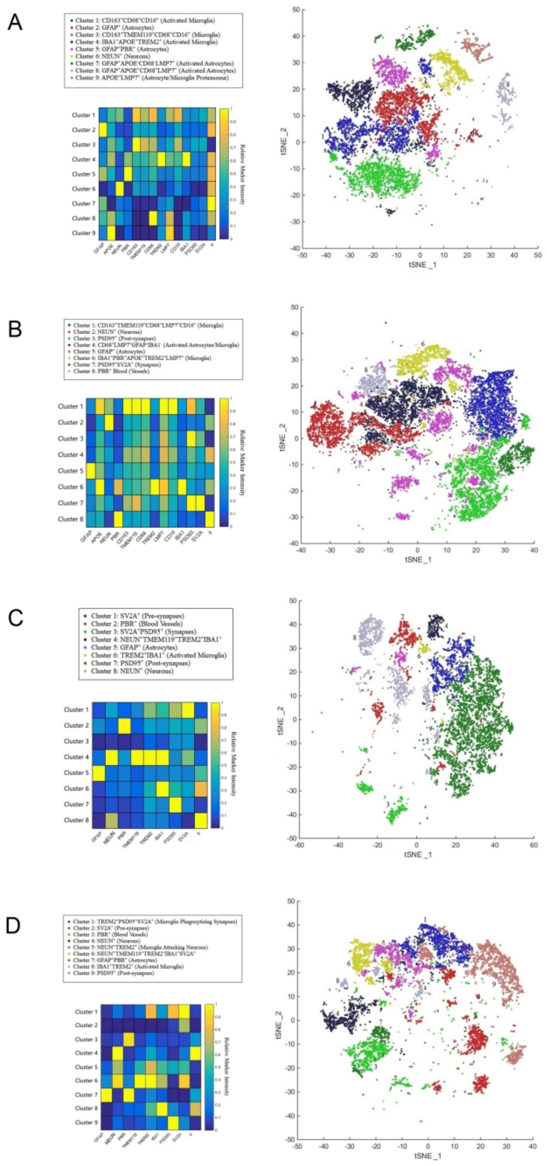

Figure 7. Spatial IMC analysis of cellular markers in AppNL-G-F mice (n=3). (A,B) Heatmap and phenograph clustering with t-distributed stochastic neighbor embedding (tSNE) in the hippocampus (A) and frontal cortex (B) of 12-month-old mice. (C,D) Heatmap and phenograph clustering with tSNE in hippocampus (C) and frontal cortex (D) of 2.5-month-old mice. Ir (intercalator) marks cell nuclei.

126x260mm (300 x 300 DPI)

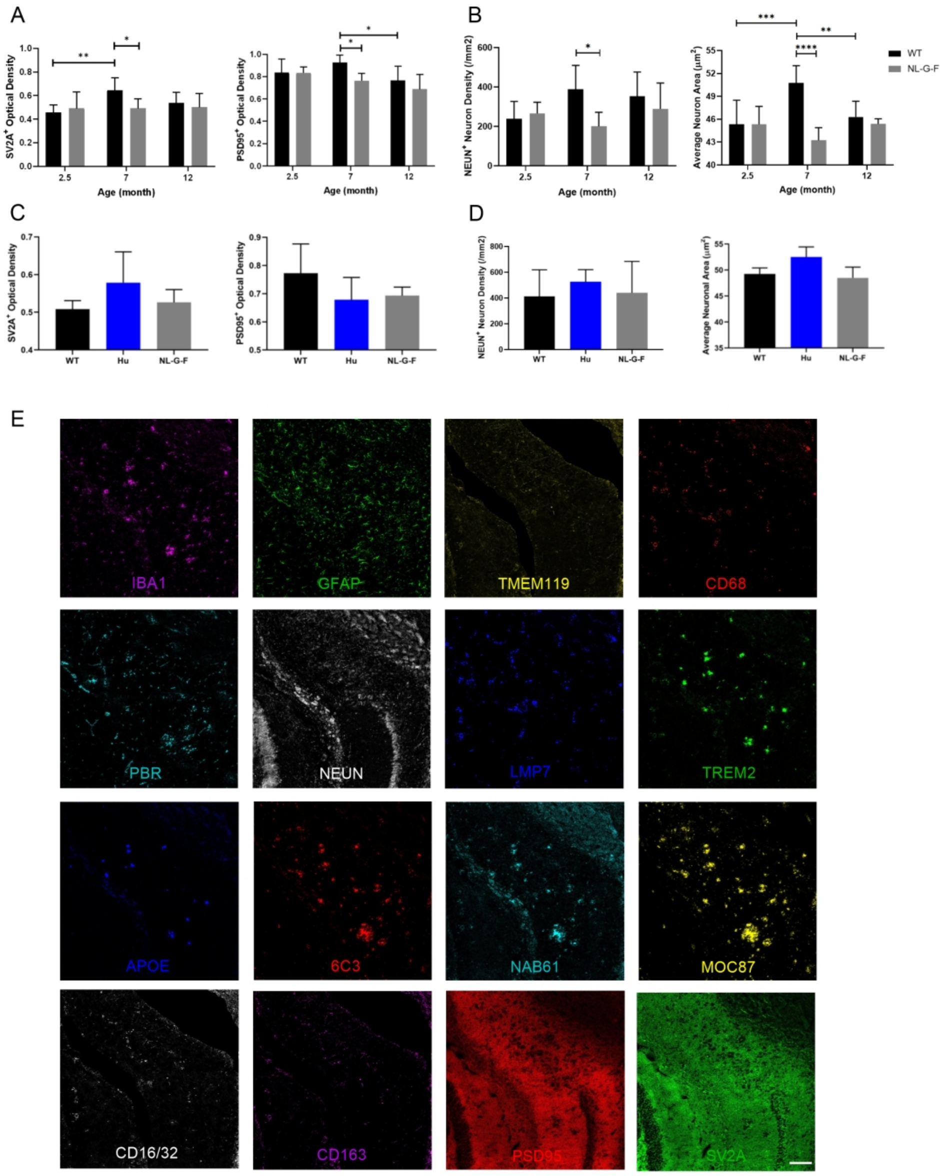

Supplementary Figure 1

218x268mm (300 x 300 DPI)

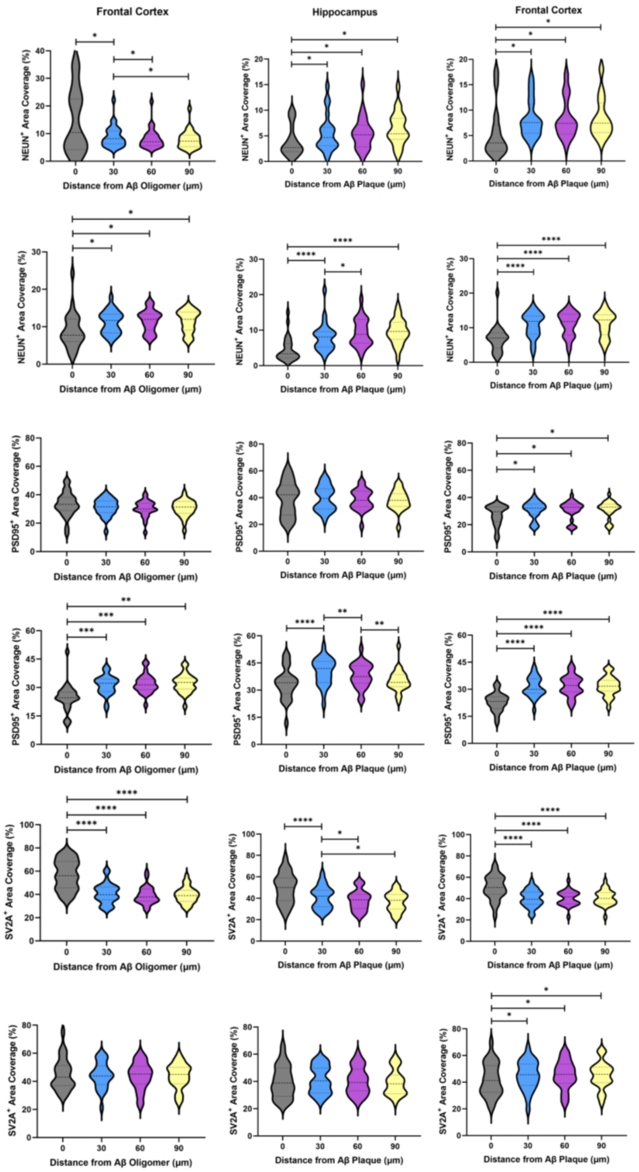

Supplementary Figure 2

158x281mm (300 x 300 DPI)

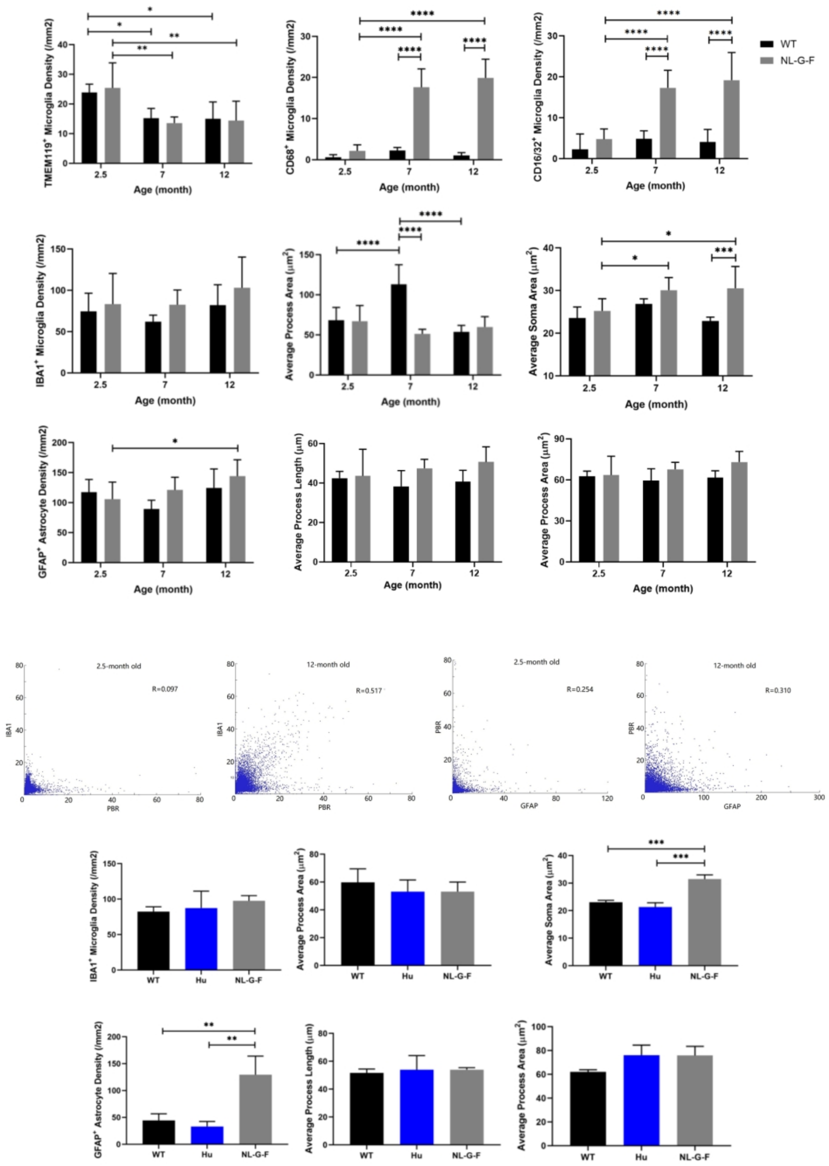

Supplementary Figure 3

189x261mm (300 x 300 DPI)

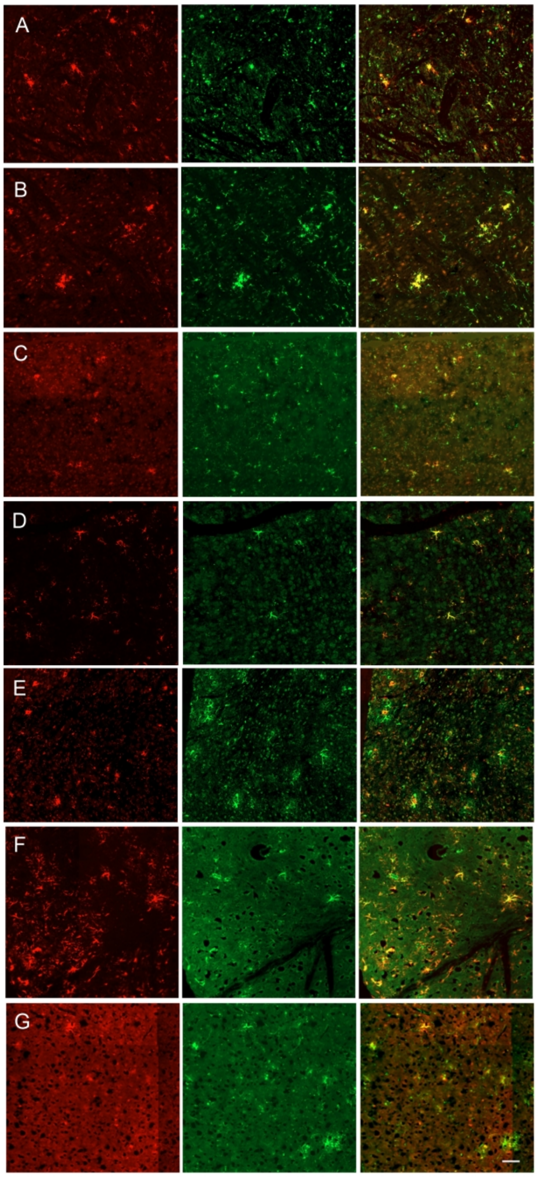

Supplementary Figure 4

132x282mm (300 x 300 DPI)

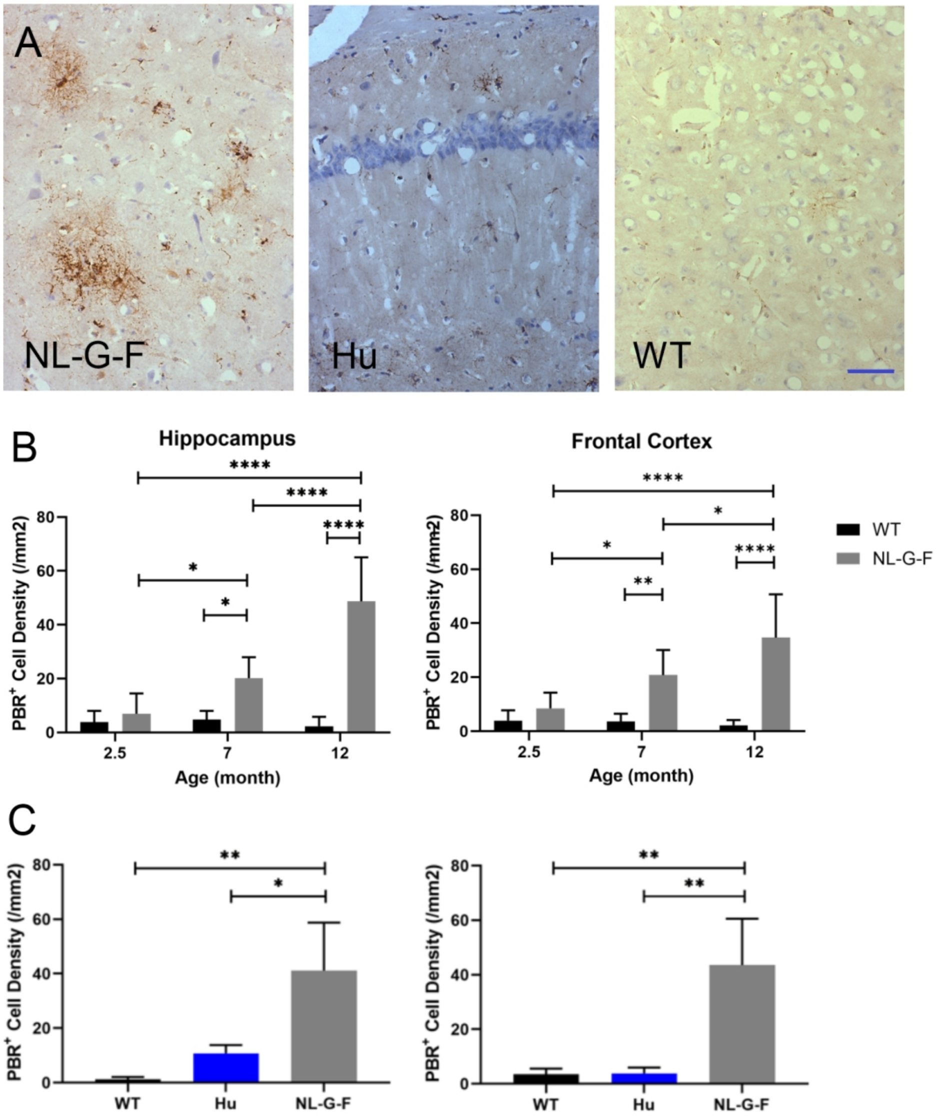

Supplementary Figure 5

134x157mm (300 x 300 DPI)

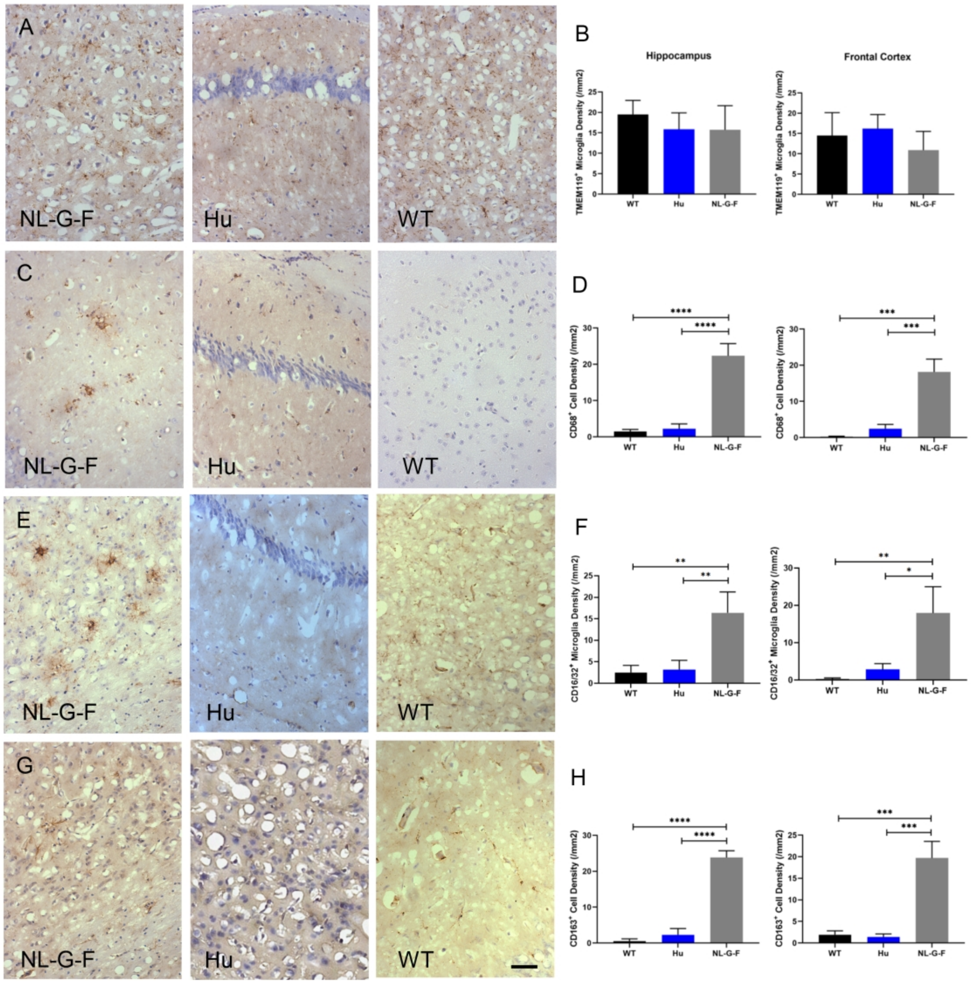

Supplementary Figure 6

219x220mm (300 x 300 DPI)

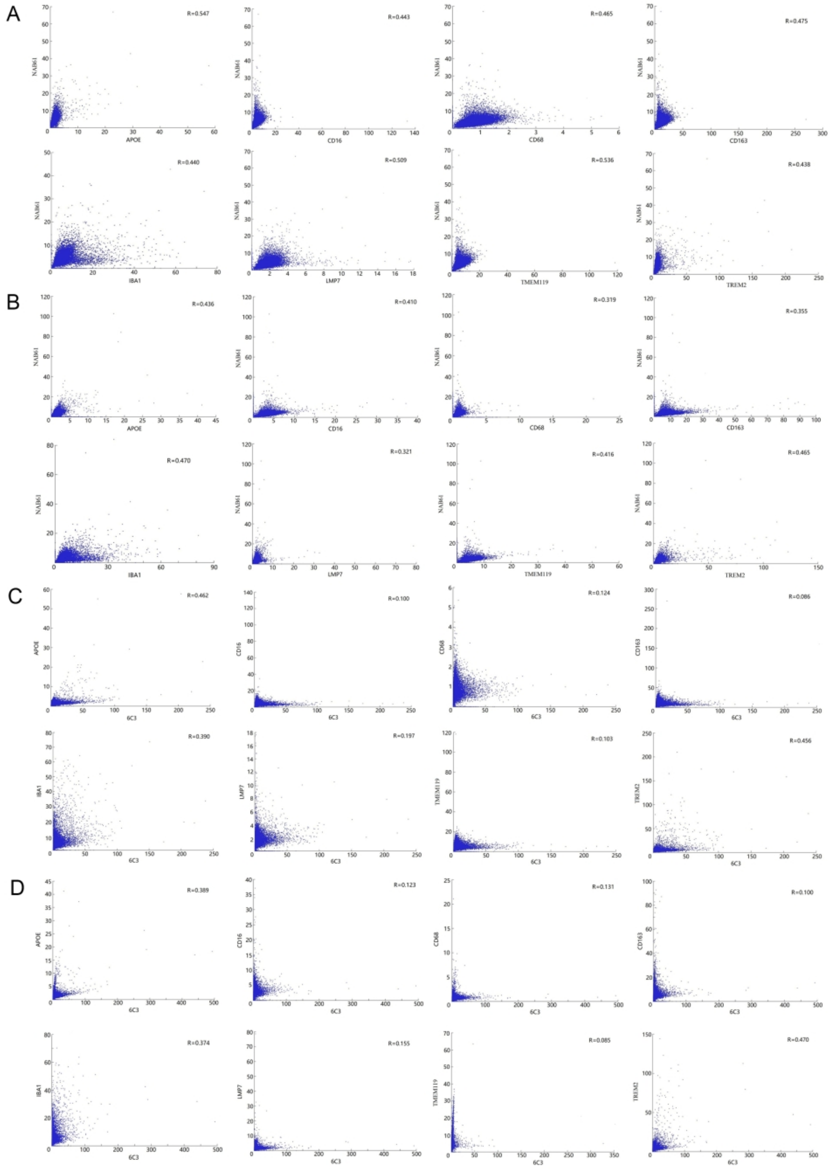

Supplementary Figure 7

200x280mm (300 x 300 DPI)

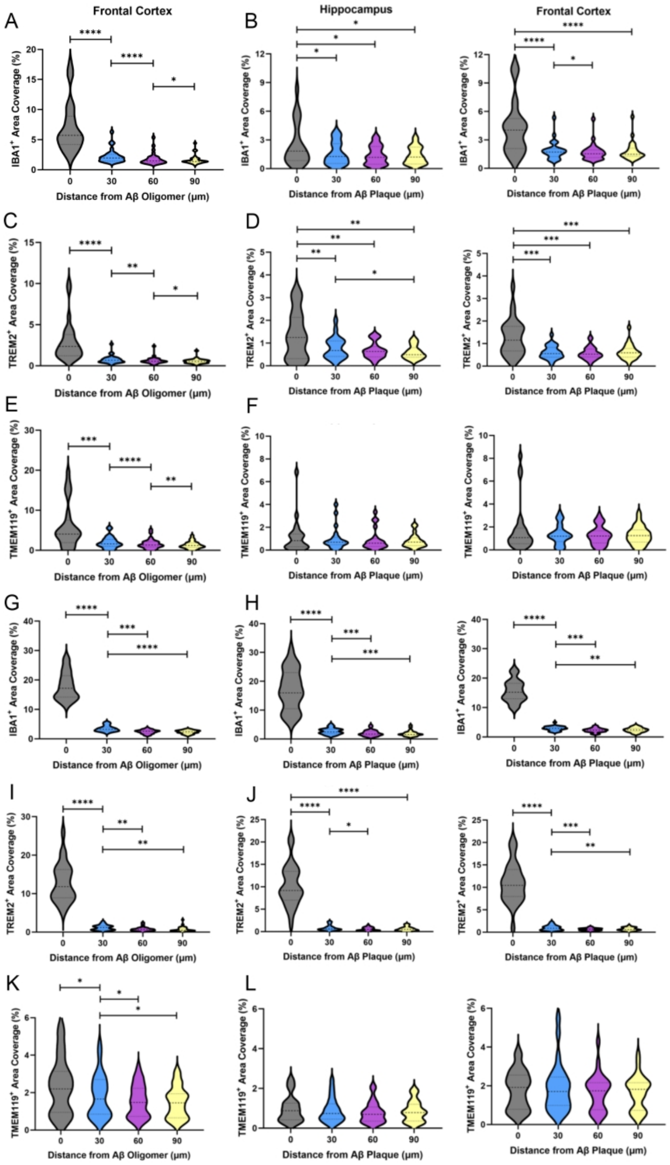

Supplementary Figure 8

166x281mm (300 x 300 DPI)

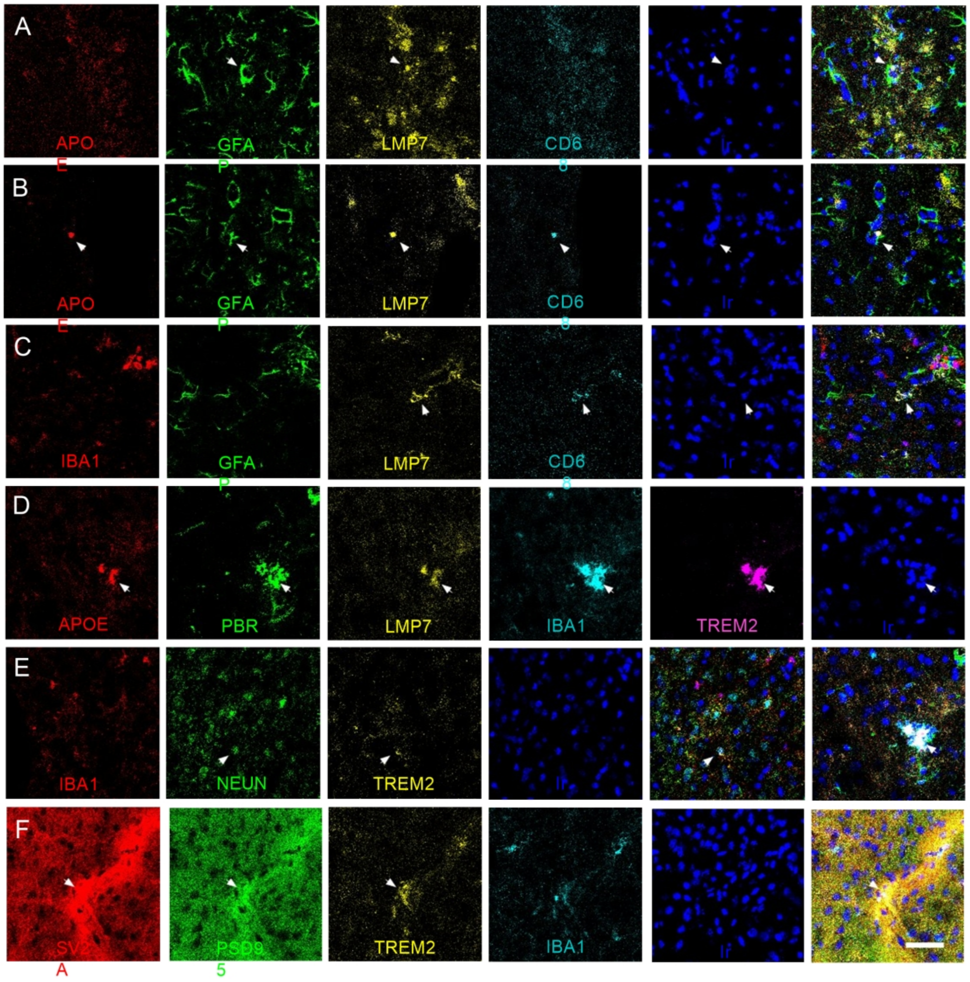

Supplementary Figure 9

195x197mm (300 x 300 DPI)

Supplementary Table 1. **PCR reaction setup.**

Component	25 µl Reaction
Q5 High-Fidelity 2X Master Mix	12.5 µl
10 µM Forward Primer	1.25 µl
10 µM Reverse Primer	1.25 µl
Template DNA	Variable
Nuclease-Free Water	to 25 µl

Supplementary Table 2. **PCR reaction thermocycling conditions.**

Step	Temperature	Time
Initial Denaturation	98°C	30 seconds
30 Cycles	98°C	10 seconds
	52°C	30 seconds
	72°C	30 seconds/kb
Final Extension	72°C	2 minutes
Hold	4–10°C	-

For Review Only

Supplementary Table 3. Primary antibody selection for IHC staining.

Antigen	Host Species	Dilution	Kit	Source	DAB Incubation
IBA1	Rabbit	1:3000	SS	Wako D19-19741	70s
GFAP	Rabbit	1:2000	SS	Dako Z0334	60s
CD16/32	Rat	1:1000	Ip	BD Biosciences 553141	3min
CD163	Rabbit	1:1000	Ip	Bioss Bs-2527R	2min
TMEM119	Rabbit	1:4000	Ip	Abcam ab209064	2min
CD68	Rat	1:2000	Ip	BIO-RAD MCA1957GA	2min
PBR	Rabbit	1:2000	Ip	Abcam ab109497	2min
6C3	Mouse	1:250	SS	Merck MABN254	4min
NAB61	Mouse	1:1000	SS	Dr. Virginia Lee from University of Pennsylvania	7min
NEUN	Mouse	1:2000	Ip	Merck MAB377	6min
SV2A	Rabbit	1:2000	SS	Abcam ab32942	90s
PSD95	Rabbit	1:2000	Ip	Abcam ab269863	30s

Supplementary Table 4. Primary antibody selection for IF staining.

Antigen	Host Species	Dilution	Source	Antigen Retrieval	Primary Incubation
IBA1	Rabbit	1:400	Wako D19-19741	No	2.5h
IBA1	Goat	1:200	Abcam ab5076	10 min FA	overnight
GFAP	Rat	1:400	Invitrogen 13-0300	No	overnight
CD16/32	Rat	1:200	BD Biosciences 553141	No	2.5h
CD163	Rabbit	1:400	Bioss Bs-2527R	No	overnight
TMEM119	Rabbit	1:400	Abcam ab209064	No	2.5h
CD68	Rat	1:200	BIO-RAD MCA1957GA	No	2.5h
PBR	Rabbit	1:200	Abcam ab109497	No	overnight

For Review Only

Supplementary Table 5. Secondary antibody selection for IF staining.

Antibody	Host Species	Dilution	Source	Fluorophore Conjugate
AlexaFluor anti-rabbit	Donkey	1:200	Invitrogen A-21206	488
AlexaFluor anti-goat	Donkey	1:200	Invitrogen A-11057	568
AlexaFluor anti-rat	Donkey	1:200	Invitrogen A-21209	594

Supplementary Table 6. Primary antibody cocktail for IMC staining.

Antigen	Dilution	Source	Metal
IBA1	1:500	Wako D19-19741	169Tm
GFAP	1:500	Dako Z0334	143Nd
CD16/32	1:50	BD Biosciences 553141	164Dy
APOE	1:100	Abcam ab227993	146Nd
6C3	1:500	MABN254	151Eu
NEUN	1:500	Merck MAB377	148Nd
TREM2	1:300	R&D AF1729	161Dy
PBR	1:500	Abcam ab213654	149Sm
SV2A	1:800	Abcam ab32942	171Yb
PSD95	1:500	Abcam ab269863	170Er
NAB61	1:300	Dr. Virginia Lee	166Er
LMP7	1:300	Santa Cruz sc-365699	162Dy
MOC87	1:500	Abcam ab251335	174Yb
CD163	1:50	Bioss Bs-2527R	154Sm
TMEM119	1:100	Abcam ab209064	155Gd
CD68	1:100	BioLegend 137002	159Tb

For Review Only

Supplementary Table 7. Colocalization area coverage surrounding Aβ plaques or oligomers with Sholl analysis.

Marker	Aβ plaques				Aβ oligomers			
	2.5 months		12 months		2.5 months		12 months	
	FC	HIP	FC	HIP	FC	HIP	FC	HIP
IBA1	4.3±2.8%	2.5±2.2%	15.6±3.7%	17.1±7.0%	7.0±4.1%	6.9±4.2%	17.9±4.7%	20.7±7.7%
TREM2	1.3±0.8%	1.4±1.0%	11.0±4.4%	10.3±4.5%	2.9±2.3%	5.7±4.1%	12.8±5.0%	12.4±5.7%
TMEM119	1.8±2.1%	1.2±1.5%	1.8±1.0%	1.0±0.7%	6.0±5.5%	8.5±5.8%	2.3±1.5%	1.3±0.9%
CD16/32	-	-	1.0±0.8%	1.1±0.8%	-	-	1.5±1.3%	1.8±1.3%
APOE	-	-	4.5±2.2%	4.6±3.9%	-	-	5.7±3.0%	7.0±5.1%
LMP7	-	-	3.5±1.4%	2.9±1.3%	-	-	4.7±2.0%	4.1±1.8%
CD68	-	-	1.2±0.7%	0.7±0.5%	-	-	1.4±1.0%	1.1±1.0%
CD163	-	-	2.3±1.2%	1.1±1.0%	-	-	3.0±1.8%	2.0±1.8%
GFAP	1.7±2.1%	3.2±2.2%	6.3±2.2%	6.7±1.9%	1.3±1.3%	4.6±2.4%	6.6±2.7%	7.0±2.5%
PBR	1.5±0.9%	1.1±0.8%	8.3±3.0%	9.7±3.5%	1.2±0.7%	1.5±1.2%	9.1±3.2%	10.2±3.6%
NEUN	5.6±5.1%	4.0±3.0%	6.7±3.7%	4.6±3.5%	14.6±11.4%	21.8±8.4%	8.9±4.7%	5.4±3.8%
SV2A	50.2±11.3%	49.7±14.0%	42.0±12.5%	39.8±11.8%	56.8±12.7%	56.3±22.7%	45.1±11.4%	43.2±14.0%
PSD95	26.9±7.5%	39.4±12.7%	23.0±5.2%	33.7±8.8%	33.3±8.6%	45.6±18.9%	24.6±7.3%	32.8±11.5%
NAB61 (Aβ oligomers)	15.8±8.0%	10.2±7.8%	20.0±6.8%	22.4±7.6%	-	-	-	-

For Review Only

Supplementary Table 8. R value summary of correlation plots.

		12 months old		2.5 months old	
Variable I	Variable II	Frontal Cortex	Hippocampus	Frontal Cortex	Hippocampus
6C3	GFAP	0.175****	0.096****	0.089****	0.021*
6C3	IBA1	0.390****	0.374****	0.152****	0.284****
6C3	CD16/32	0.100****	0.123****	-	-
6C3	CD163	0.086****	0.100****	-	-
6C3	CD68	0.124****	0.131****	-	-
6C3	APOE	0.462****	0.389****	-	-
6C3	LMP7	0.197****	0.155****	-	-
6C3	PBR	0.296****	0.263****	0.035****	0.037****
6C3	TMEM119	0.103****	0.085****	0.172****	0.429****
6C3	TREM2	0.456****	0.470****	0.122****	0.244****
NAB61	GFAP	0.221****	0.134****	0.096****	0.045****
NAB61	IBA1	0.440****	0.470****	0.668****	0.729****
NAB61	CD16/32	0.443****	0.410****	-	-
NAB61	CD163	0.475****	0.355****	-	-
NAB61	CD68	0.465****	0.319****	-	-
NAB61	APOE	0.547****	0.436****	-	-
NAB61	LMP7	0.509****	0.321****	-	-
NAB61	PBR	0.250****	0.220****	0.059****	0.104****
NAB61	TMEM119	0.536****	0.416****	0.795****	0.823****
NAB61	TREM2	0.438****	0.465****	0.609****	0.725****
NAB61	6C3	0.696****	0.732****	0.331****	0.414****
GFAP	APOE	0.255****	0.200****	-	-
GFAP	CD163	0.111****	0.071****	-	-
GFAP	LMP7	0.103****	0.108****	-	-
GFAP	PBR	0.341****	0.258****	0.254****	0.307****
IBA1	PBR	0.517****	0.459****	0.097****	0.148****
IBA1	TREM2	0.667****	0.638****	0.768****	0.738****
IBA1	CD163	0.234****	0.212****	-	-
IBA1	CD16/32	0.277****	0.279****	-	-
IBA1	APOE	0.496****	0.387****	-	-
IBA1	LMP7	0.445****	0.355****	-	-
IBA1	CD68	0.298****	0.251****	-	-

|R|>0.5 is strong correlation. 0.3<|R|<0.5 is moderate correlation. 0.1<|R|<0.3 is weak correlation. **** p<0.0001, *** p<0.001, ** p<0.01, * p<0.05.

For Review Only

Supplementary File: Code for Sholl analysis in ImageJ.

```
Stack.setXUnit("um");
run("Properties...", "channels=1 slices=1 frames=1 pixel_width=1.0000 pixel_height=1.0000 voxel_depth=25400.0508");

getVoxelSize(px, py, pz, unit);

//Dialog.create("Options");
//Dialog.addNumber("Enter numbr of required rings", 3);
//Dialog.addNumber("Enter thickness of rings ("+unit+")", 30);
//Dialog.show();

number_of_rings=3;
increment=30;

//get original ROIs and combine
n=roiManager("count");
orig_rois=Array.getSequence(n);
roiManager("select", orig_rois);
roiManager("Combine");
roiManager("Add");

//remove riginal ROIs
//roiManager("select", orig_rois);
//roiManager("delete");

for (ring=0;ring<number_of_rings;ring++){

//create enlargements
roiManager("select", n+ring);
roiManager("rename", "Region "+ring);
run("Enlarge...", "enlarge="+increment);
roiManager("Add");
}
roiManager("select", n+ring);
roiManager("rename", "Region "+ring);

//create rings
for (ring=0;ring<number_of_rings;ring++){
roiManager("Select", newArray(n+ring,n+ring+1));
roiManager("XOR");
roiManager("Add");
}
```

For Review Only

```
roiManager("deselect");
roiManager("Set Fill Color", "#4d00ff00");
//label rings
for (ring=0;ring<number_of_rings;ring++){
roiManager("select", n+number_of_rings+ring+1);
roiManager("rename", "Ring "+ring+1);
}

roiManager("select", orig_rois);
RoiManager.setPosition(0);
roiManager("Set Color", "white");
roiManager("Set Line Width", 0);

for (ring=0;ring<number_of_rings;ring++){
roiManager("select", n+ring);
RoiManager.setPosition(0);
roiManager("Set Color", "white");
roiManager("Set Line Width", 0);
}

for (ring=0;ring<number_of_rings;ring++){
roiManager("Select", newArray(n+ring,n+ring+1));
RoiManager.setPosition(0);
roiManager("Set Color", "white");
roiManager("Set Line Width", 0);
}

for (ring=0;ring<number_of_rings;ring++){
roiManager("select", n+number_of_rings+ring+1);
RoiManager.setPosition(0);
roiManager("Set Color", "white");
roiManager("Set Line Width", 0);
}

saveAs("Tiff", "");
roiManager("Select", 1);
run("Select All");
roiManager("Deselect");
roiManager("Delete");
close();
```

For Review Only
