# Supplementary material for: Associations of amyloid-β oligomers and plaques with neuropathology in the AppNL-G-F mouse
Source: Brain Commun. 2024 Jun 25;6(4):fcae218. doi: 10.1093/braincomms/fcae218 (PMC11258573; doi:10.1093/braincomms/fcae218)
Supplement: fcae218_Supplementary_Data [file fcae218_supplementary_data.zip › Supplementary_material.pdf]

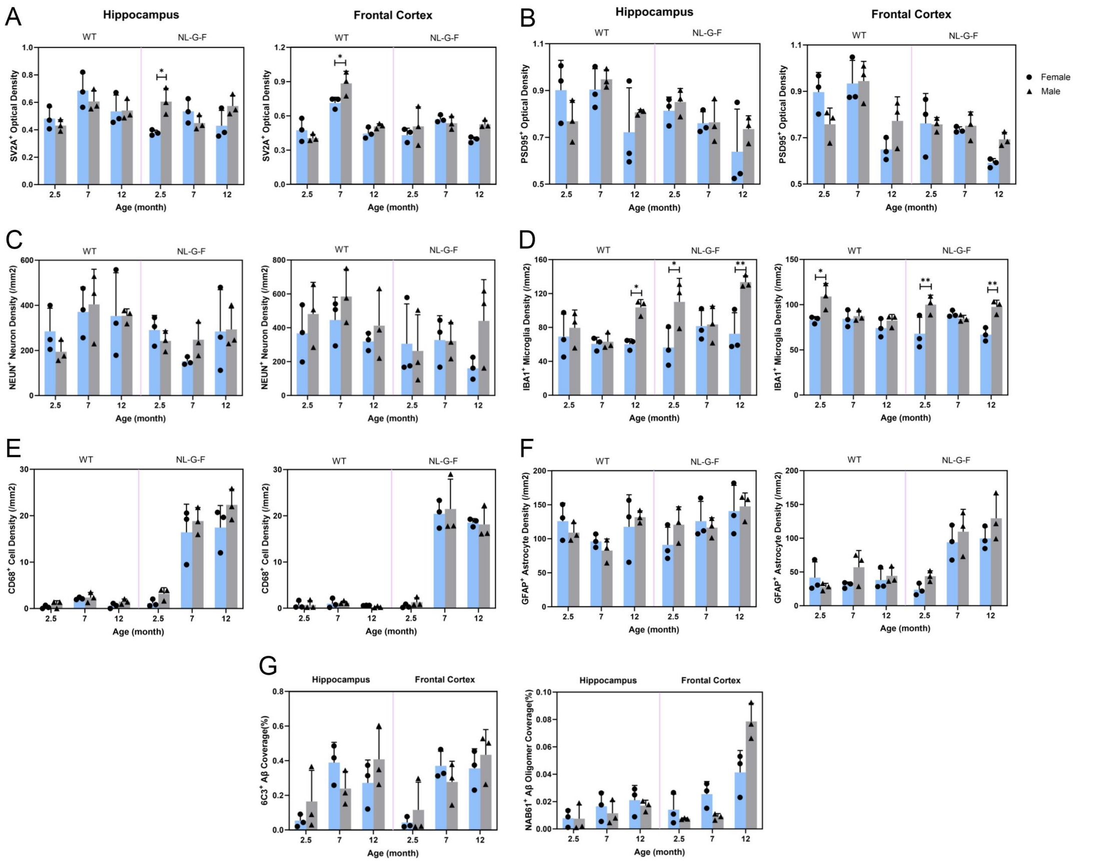

Supplementary Figure 1. Comparisons between pathological measures in male and female App$^{NL\text{-}G\text{-}F}$ and WT mice (n=3 for each). (A) SV2A^{+} pre-synaptic optical density. **(B)** PSD95^{+} post-synaptic optical density. **(C)** NeuN^{+} neuronal density. **(D)** Overall microglia (IBA1^{+}) density. **(E)** Activated microglia (CD68^{+}) density. **(F)** Overall astrocyte (GFAP^{+}) density. **(G)** Aβ coverage. Blue bars and filled circles are from female mice. Grey bars and triangles are from male mice. Columns express mean ± SD, statistical analysis was performed using three-way ANOVA. Density is calculated as cell count/area. Each data point represents a mean from three technical replicates in one mouse.

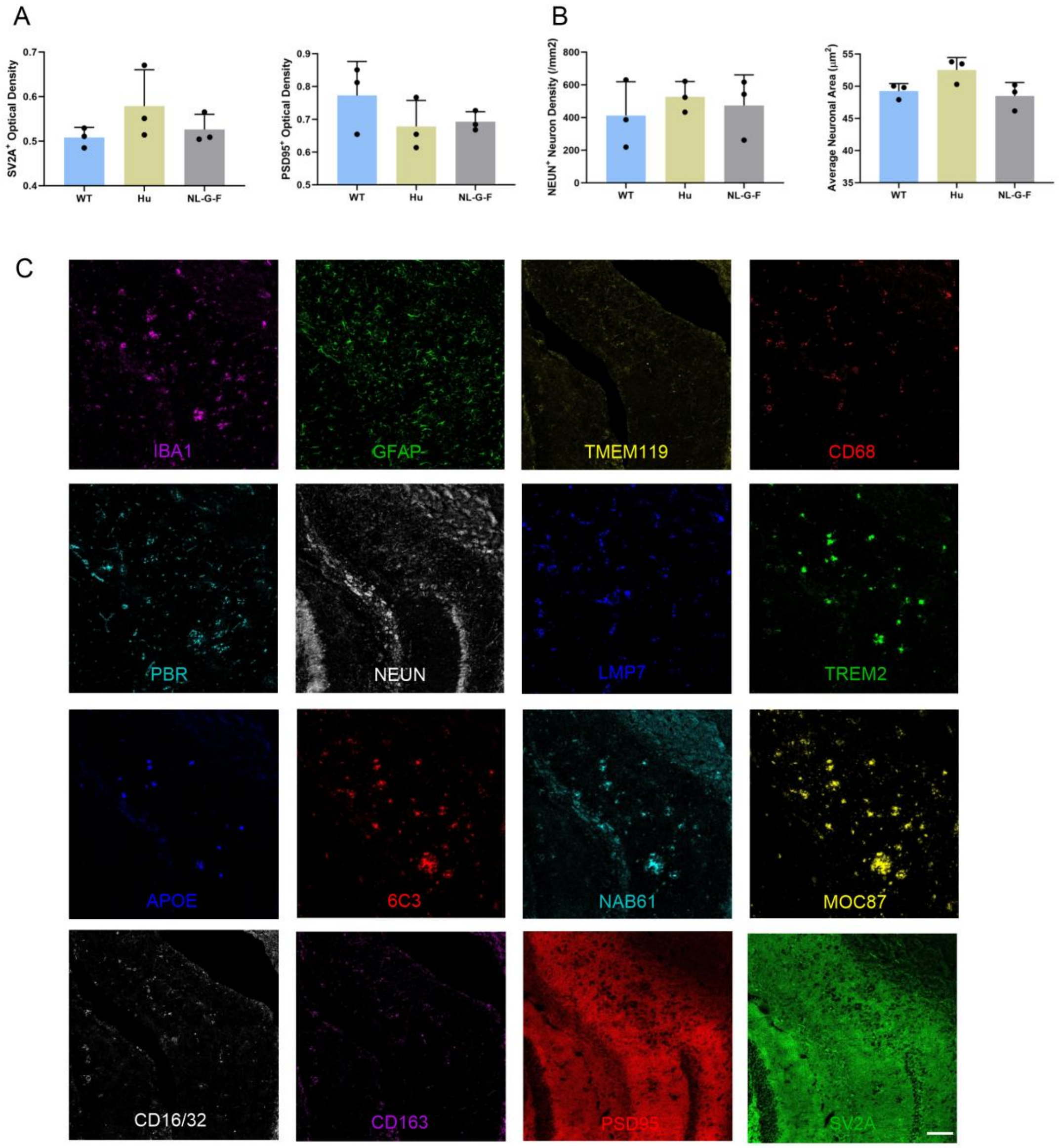

Supplementary Figure 2. Comparison of neuronal and synaptic changes in the hippocampus and frontal cortex of *App*$^{NL\text{-}G\text{-}F}$, *App*^{hu} and WT mice. **(A)** Optical density of the pre-synaptic SV2A^{+} and post-synaptic PSD95^{+} signals in the frontal cortex at 12 months (n=3). SV2A: $F(2,6)=1.44$, $P=0.3083$. PSD95: $F(2,6)=1.28$, $P=0.3444$. **(B)** NEUN^{+} neuronal density and average neuronal area in the frontal cortex at 12 months (n=3). Density: $F(2,6)=0.3378$, $P=0.7261$. Area: $F(2,6)=4.377$, $P=0.0672$. **(C)** Representative IMC images of 16 markers. Columns represent the mean ± SD, statistical analysis was performed using two-way ANOVA. Density is calculated as cell count/area. Scale bar = 100 µm. Each data point represents a mean from three technical replicates in one mouse.

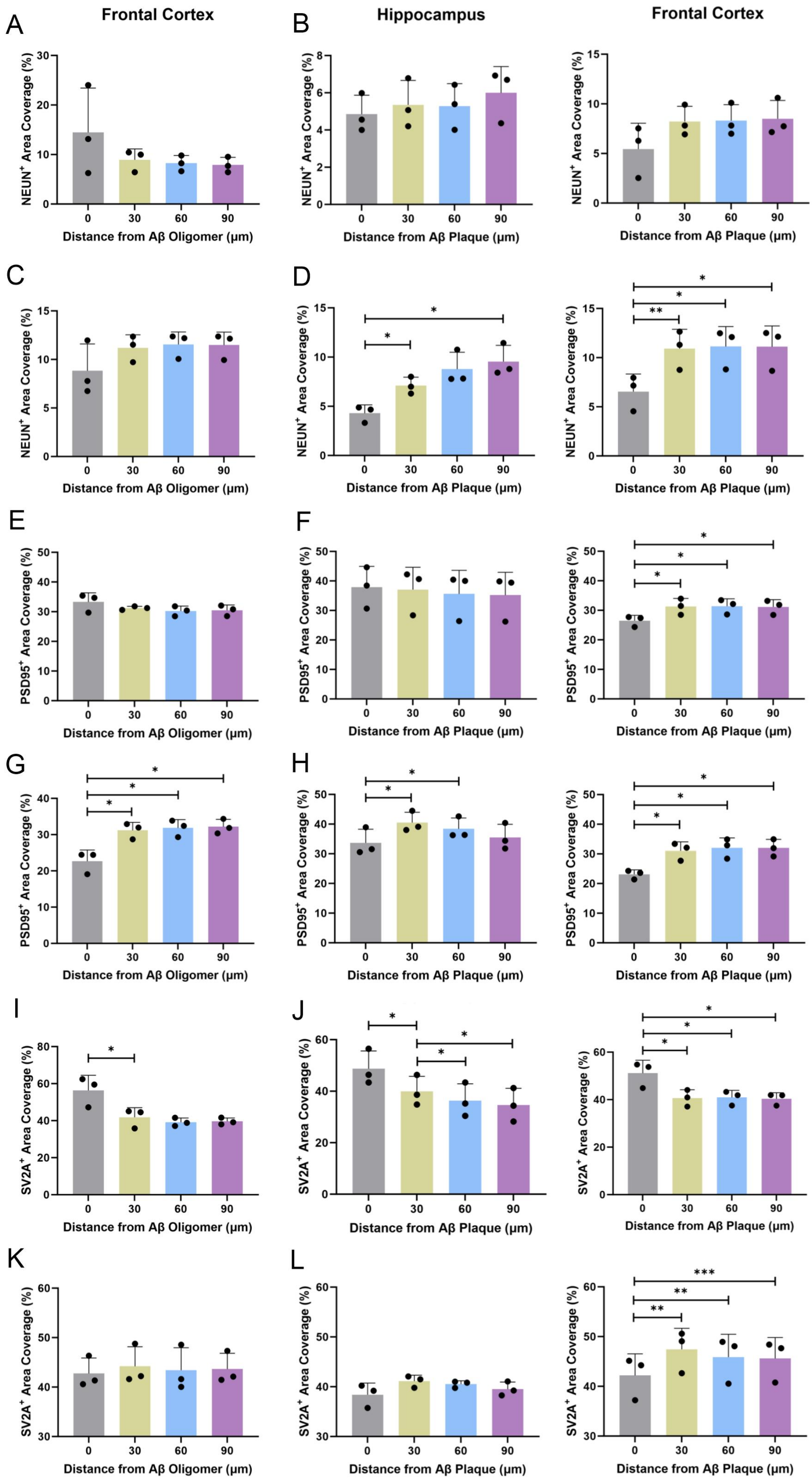

Frontal Cortex
Hippocampus
Frontal Cortex
A
B
C
D
E
F
G
H
I
J
K
L
NEUN+ Area Coverage (%)
PSD95+ Area Coverage (%)
SV2A+ Area Coverage (%)
Distance from Aβ Oligomer (μm)
Distance from Aβ Plaque (μm)

Supplementary Figure 3. Sholl analysis of Aβ proteins and neuronal pathology in *App*$^{NL-G-F}$ mice (n=3). **(A)** Sholl analysis of NEUN$^+$ neurons and Aβ oligomers in 2.5-month-old mice. F(1.001,2.003)=1.676. P=0.3247. **(B)** Sholl analysis of NEUN$^+$ neurons and Aβ plaques in 2.5-month-old mice. HIP: F(3,8)=0.4276, P=0.7388. FC: F(3,8)=1.681, P=0.2474. **(C)** Sholl analysis between NEUN$^+$ neurons and Aβ oligomers in 12-month-old mice. F(1.007,2.014)=4.784, P=0.1595. **(D)** Sholl analysis between NEUN$^+$ neurons and Aβ plaques in 12-month-old mice. HIP: F(1.194,2.388)=37.22, P=0.0164. FC: F(1.042,2.084)=75.36, P=0.0115. **(E)** Sholl analysis of PSD95$^+$ synapses and Aβ oligomers in 2.5-month-old mice. F(1.009,2.017)=1.248, P=0.3801. **(F)** Sholl analysis of PSD95$^+$ synapses and Aβ plaques in 2.5-month-old mice. HIP: F(1.006,2.012)=1.977, P=0.2947. FC: F(1.231,2.462)=98.64, P=0.0047. **(G)** Sholl analysis of PSD95$^+$ synapses and Aβ oligomers in 12-month-old mice. F(1.043,2.085)=97.93, P=0.0087. **(H)** Sholl analysis of PSD95$^+$ synapses and Aβ plaques in 12-month-old mice. HIP: F(1.209,2.419)=30.55, P=0.0198. FC: F(1.341,2.683)=47.88, P=0.0084. **(I)** Sholl analysis of SV2A$^+$ synapses and Aβ oligomers in 2.5-month-old mice. F(1.021,2.041)=21.25, P=0.0423. **(J)** Sholl analysis of SV2A$^+$ synapses and Aβ plaques in 2.5-month-old mice. HIP: F(1.475,2.949)=164.4, P=0.001. FC: F(1.31,2.62)=44.74, P=0.0099. **(K)** Sholl analysis of SV2A$^+$ synapses and Aβ oligomers in 12-month-old mice. F(1.498,2.996)=1.494, P=0.3374. **(L)** Sholl analysis of SV2A$^+$ synapses and Aβ plaques in 12-month-old mice. HIP: F(1.072,2.143)=2.659, P=0.2394. FC: F(1.694,3.388), P=0.0003. Columns represent the mean ± SD, statistical analysis was performed using one-way ANOVA. HIP, hippocampus. FC, frontal cortex. Each data point represents a mean from three technical replicates in one mouse.

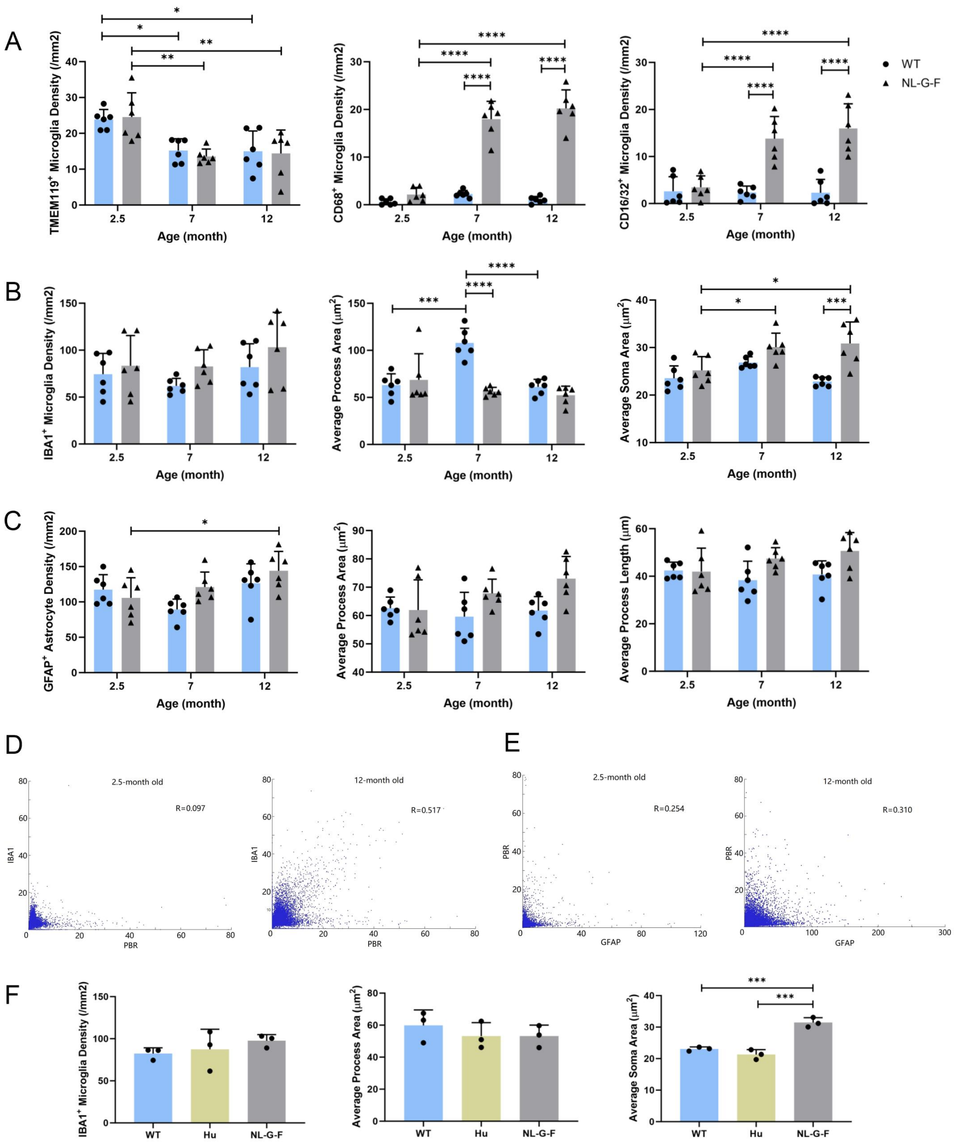

Supplementary Figure 4. Age-related changes of glial density and morphology in *App*$^{NL-G-F}$, *App*^{hu} and WT mice. (A) Phenotypic microglia density in the hippocampus (n=6). TMEM119: F(2,30)=15.8, P<0.0001. CD68: F(2,30)=48.16, P<0.0001. CD16/32: F(2,30)=8.205, P=0.0014. **(B)** IBA1$^+$ microglia density and morphology in the hippocampus (n=6). Density: F(2,30)=0.22, P=0.8038. Process: F(2,30)=16.64, P<0.0001. Soma: F(2,30)=4.261, P=0.0235. **(C)** GFAP$^+$ astrocyte density and morphology in the hippocampus (n=6). Density: F(2,30)=5.253, P=0.0111. Area: F(2,30)=2.216, P=0.1266. Length: F(2,30)=2.122, P=0.1374. **(D, E)** Correlation plots between PBR$^+$ and IBA1$^+$ microglia **(D)** or GFAP$^+$ astrocytes **(E)** in the frontal cortex of *App*$^{NL-G-F}$ mice (n=3). **(F)** IBA1$^+$ microglia density and morphology in the frontal cortex of 12-month-old mice (n=3). Density: F(2,6)=0.8034, P=0.4907. Process: F(2,6)=0.6295, P=0.5647. Soma: F(2,6)=51.08, P=0.0002. Columns represent the mean ± SD. Statistical analysis was performed using two-way ANOVA. Density is calculated as cell count/area. Each data point represents the mean of three technical replicates in one mouse.

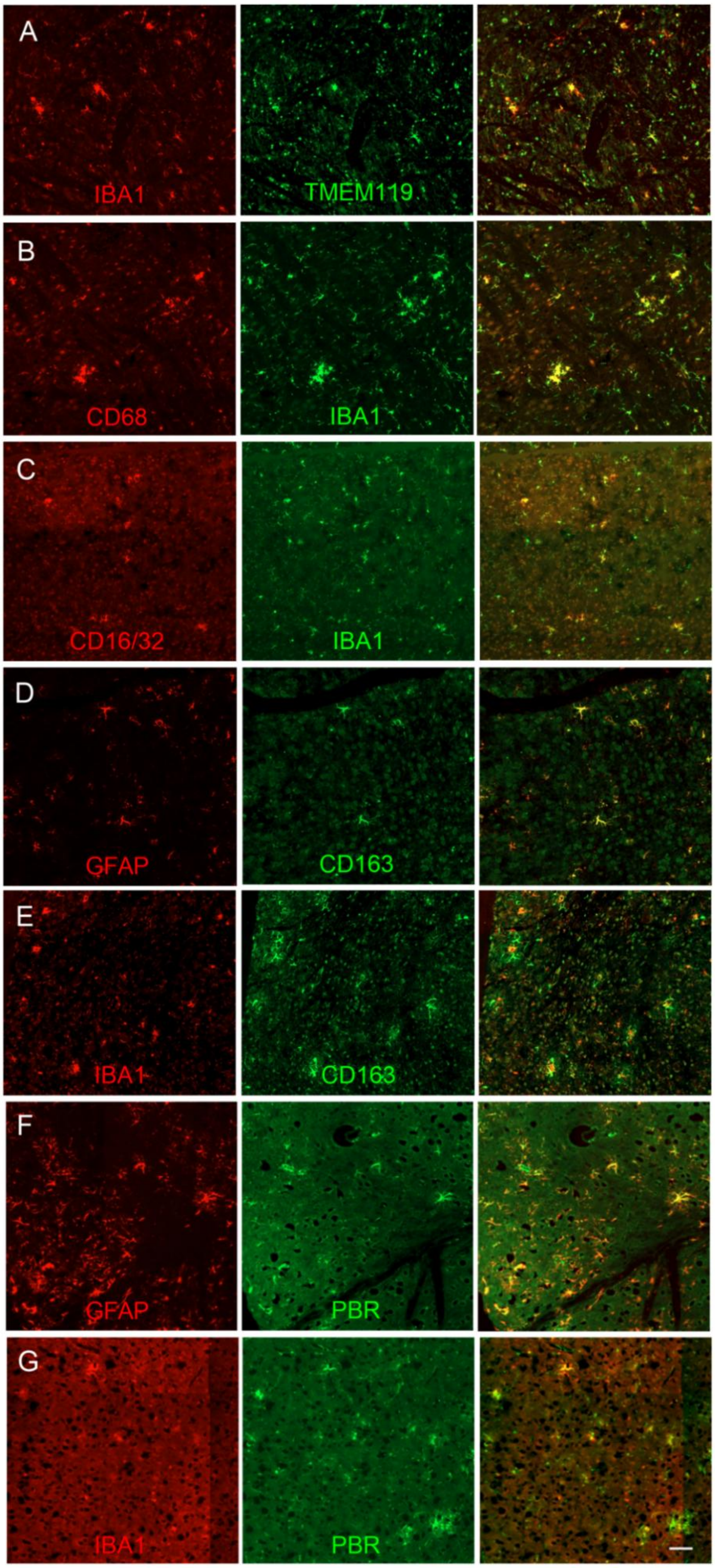
A
IBA1
TMEM119
B
CD68
IBA1
C
CD16/32
IBA1
D
GFAP
CD163
E
IBA1
CD163
F
GFAP
PBR
G
IBA1
PBR

Supplementary Figure 5. Immunofluorescence staining reveals distinct cell types with different patterns of phenotypic marker expression. (A) Double staining images of IBA1 (red) and TMEM119 (green). **(B)** Double staining images of CD68 (red) and IBA1 (green). **(C)** Double staining images of CD16/32 (red) and IBA1 (green). **(D)** Double staining images of GFAP (red) and CD163 (green). **(E)** Double staining images of IBA1 (red) and CD163 (green). **(F)** Double staining images of GFAP (red) and PBR (green). **(G)** Double staining images of IBA1 (red) and PBR (green). Scale bar = 100 μm.

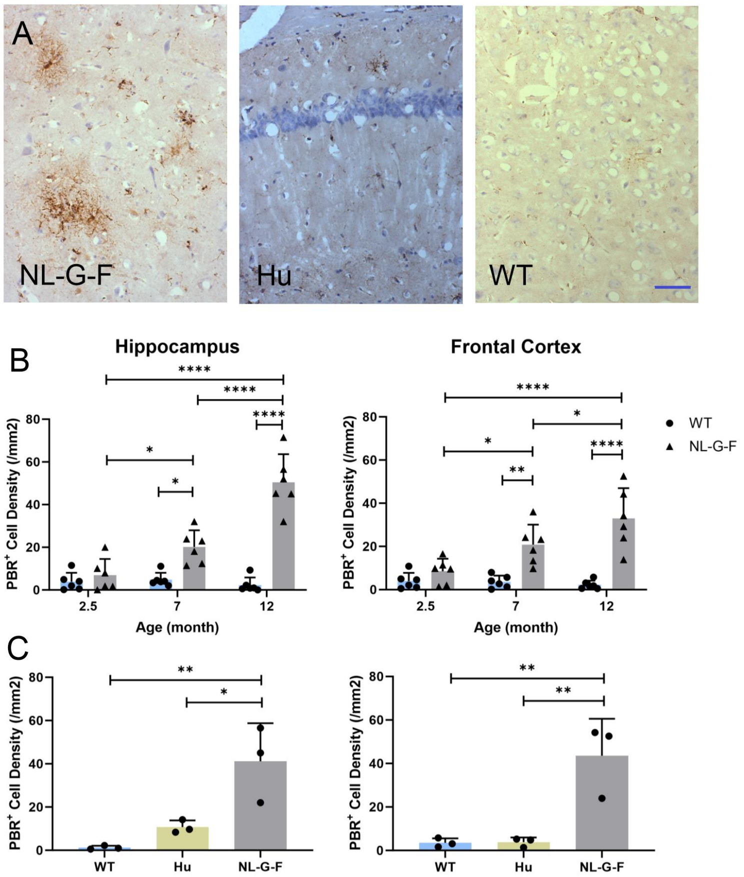

Supplementary Figure 6. Age-related changes of PBR$^+$ glial density in *App*$^{NL\text{-}G\text{-}F}$, *App*^{hu} and WT mice. (A) IHC staining images of PBR$^+$ proinflammatory microglia and astrocytes. **(B)** Age-related changes of PBR$^+$ cell density in *App*$^{NL\text{-}G\text{-}F}$ and WT mice (n=6). HIP: F(2,30)=29.21, P<0.0001. FC: F(2,30)=9.141, P=0.0008. **(C)** PBR$^+$ cell density in 12-month-old *App*$^{NL\text{-}G\text{-}F}$, *App*^{hu} and WT mice. HIP: F(2,6)=12.26, P=0.0076. FC: F(2,6)=16.05, P=0.0039. Columns represent the mean ± SD. Statistical analysis was performed using two-way ANOVA. Density is calculated as cell count/area. Scale bar = 50 μm. HIP, hippocampus. FC, frontal cortex. Each data point represents a mean from three technical replicates in one mouse.

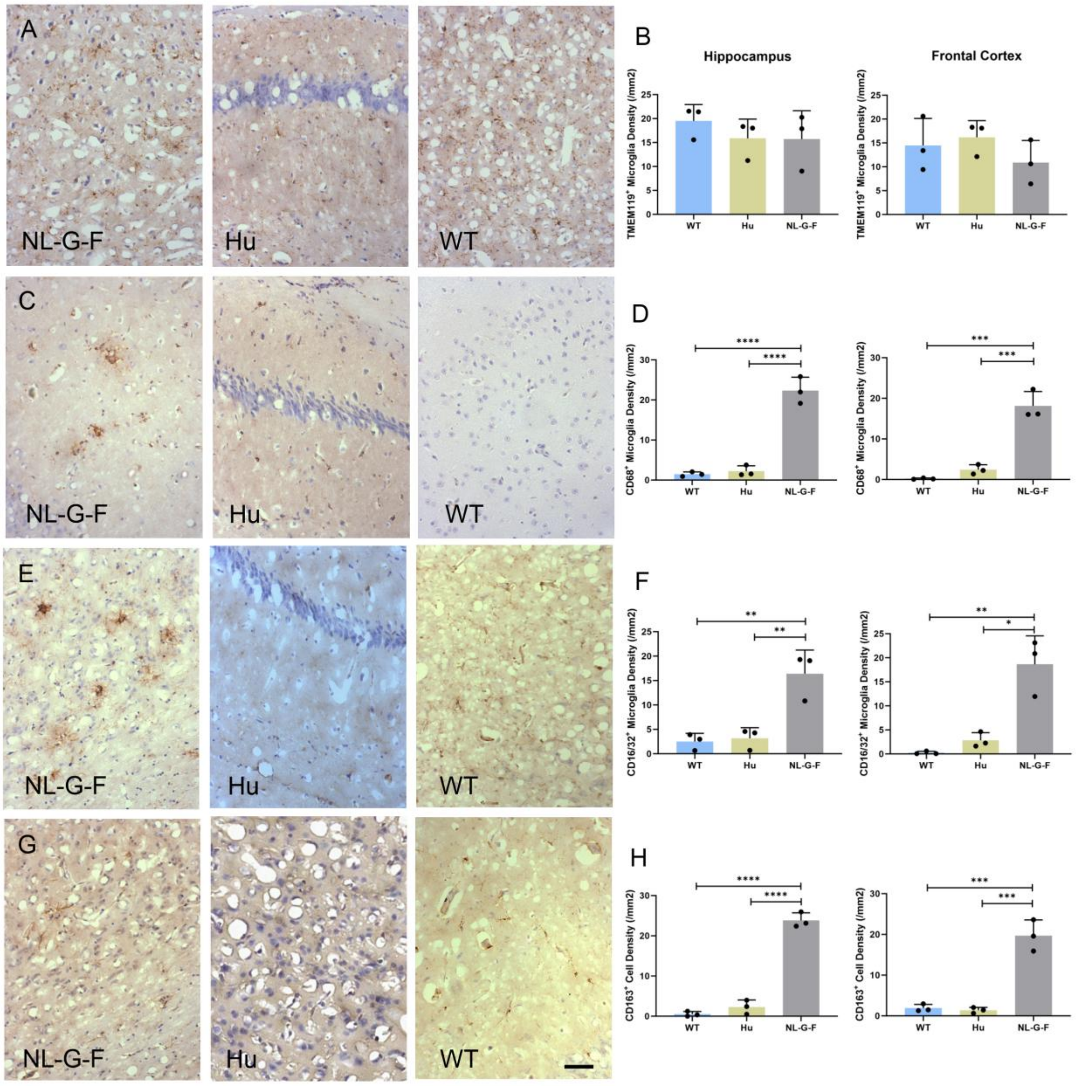

Supplementary Figure 7. Comparison of different glial phenotypes in the hippocampus and frontal cortex of *App*$^{NL\text{-}G\text{-}F}$, *App*^{hu} and WT mice (n=3). (A) IHC staining images of TMEM119$^+$ inactive microglia. **(B)** TMEM119$^+$ microglia density. HIP: $F(2,6)=0.6584$, $P=0.5514$. FC: $F(2,6)=0.996$, $P=0.4231$. **(C)** IHC staining images of CD68$^+$ activated microglia. **(D)** CD68$^+$ microglia density. HIP: $F(2,6)=94$, $P<0.0001$. FC: $F(2,6)=61.38$, $P=0.0001$. **(E)** IHC staining images of CD16/32$^+$ proinflammatory microglia. **(F)** CD16/32$^+$ microglia density. HIP: $F(2,6)=17.89$, $P=0.003$. FC: $F(2,6)=23.84$, $P=0.0014$. **(G)** IHC staining images of CD163$^+$ anti-inflammatory glial cells. **(H)** CD163$^+$ cell density. HIP: $F(2,6)=217$, $P<0.0001$. FC: $F(2,6)=60.4$, $P=0.0001$. Columns represent the mean ± SD. Statistical analysis was performed using one-way ANOVA. Density is calculated as cell count/area. Scale bar = 50 µm. HIP, hippocampus. FC, frontal cortex. Each data point represents a mean from three technical replicates in one mouse.

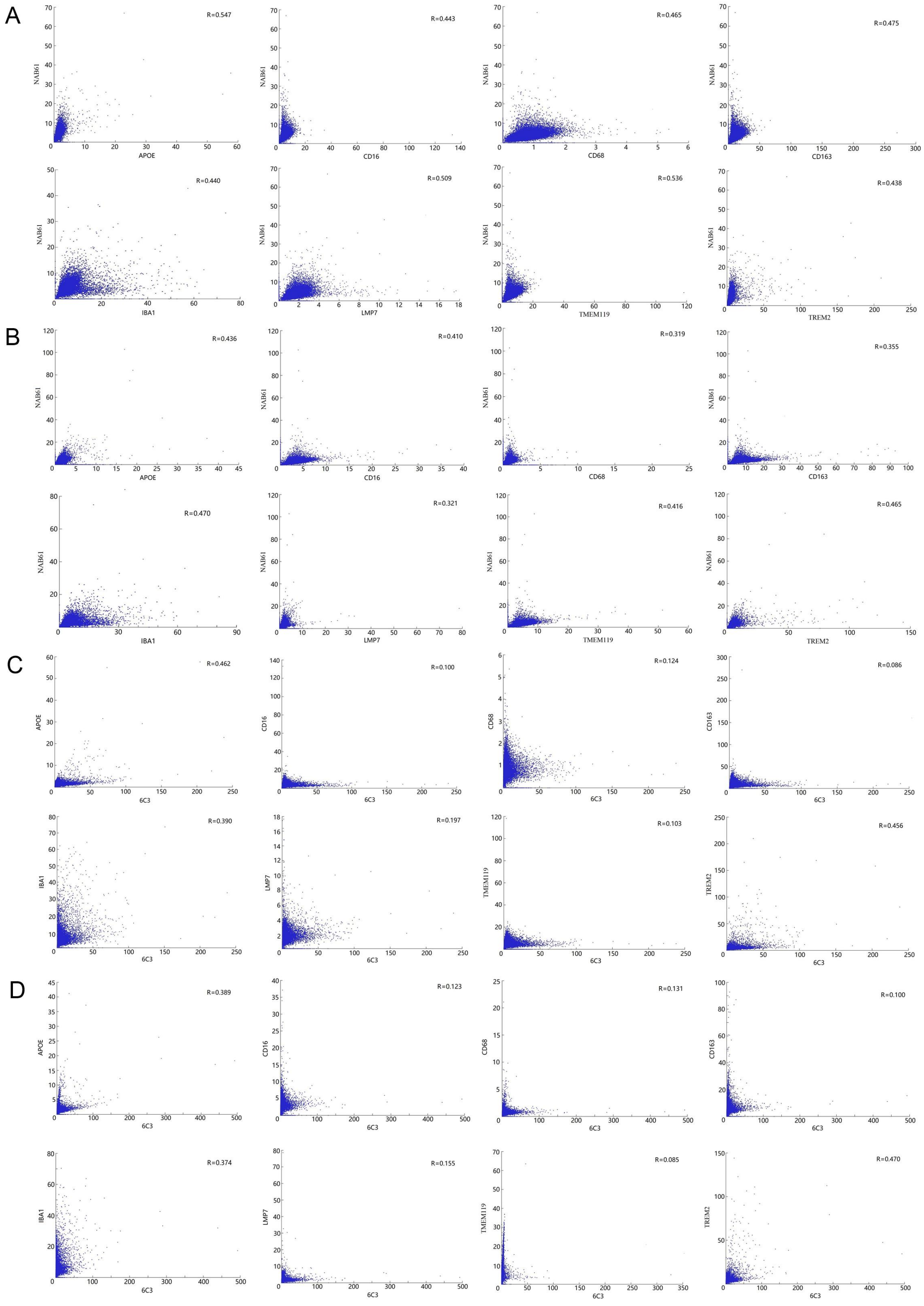

Supplementary Figure 8. Correlation between microglia markers and Aβ in 12-month-old *App*$^{NL\text{-}G\text{-}F}$ mice (n=3). (A, B) Correlation plots between NAB61^{+} Aβ oligomers and all microglia markers in the frontal cortex **(A)** and hippocampus **(B)**. **(C, D)** Correlation plots between 6C3^{+} Aβ plaques and all microglia markers in the frontal cortex **(C)** and hippocampus **(D)**.

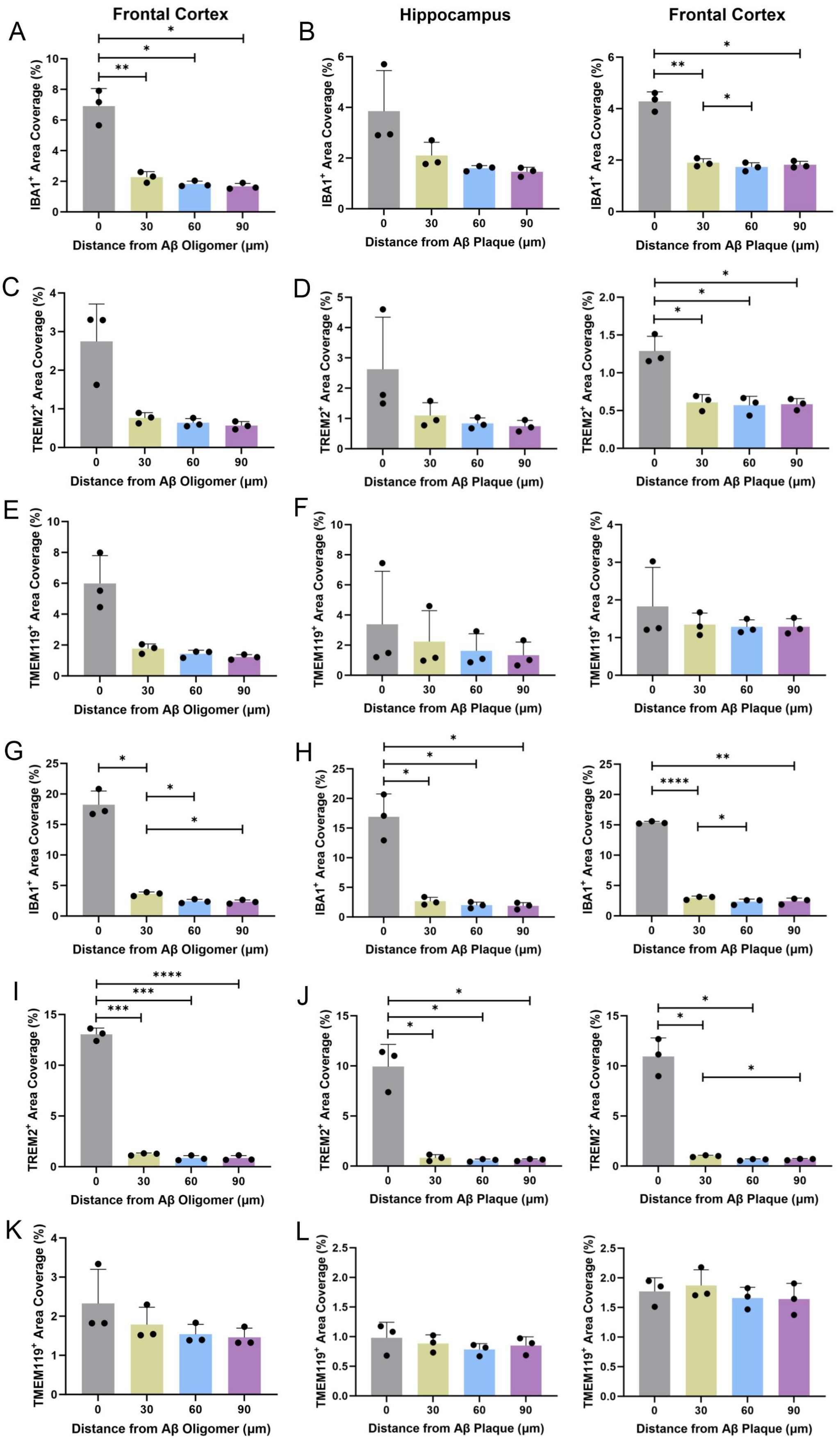
Frontal Cortex
Hippocampus
Frontal Cortex
A
B
C
D
E
F
G
H
I
J
K
L
IBA1+ Area Coverage (%)
TREM2+ Area Coverage (%)
TMEM119+ Area Coverage (%)
Distance from Aβ Oligomer (μm)
Distance from Aβ Plaque (μm)
0
30
60
90
*
**
***
****

Supplementary Figure 9. Sholl analysis of Aβ pathology and microglia markers in *App*$^{NL\text{-}G\text{-}F}$ mice of different ages (n=3). (A) Sholl analysis of IBA1^{+} microglia and Aβ oligomers in 2.5-month-old mice. F(1.013,2.026)=62.36, P=0.0151. **(B)** Sholl analysis of IBA1^{+} microglia and Aβ plaques in 2.5-month-old mice. HIP: F(1.002,2.003)=5.73, P=0.1388. FC: F(1.03,2.06)=270.5, P=0.0032. **(C)** Sholl analysis of TREM2^{+} microglia and Aβ oligomers in 2.5-month-old mice. F(1.003,2.006)=15.38, P=0.059. **(D)** Sholl analysis of TREM2^{+} microglia and Aβ plaques in 2.5-month-old mice. HIP: F(3,8)=2.898, P=0.1017. FC: F(3,5.235)=21.22, P=0.0024. **(E)** Sholl analysis of TMEM119^{+} microglia and Aβ oligomers in 2.5-month-old mice. F(1.01,2.02)=21.05, P=0.0436. **(F)** Sholl analysis of TMEM119^{+} microglia and Aβ plaques in 2.5-month-old mice. HIP: F(1.002,2.003)=1.697, P=0.3224. FC: F(1.059,2.118)=1.204, P=0.3876. **(G)** Sholl analysis of IBA1^{+} microglia and Aβ oligomers in 12-month-old mice. F(3,2.239)=134.8, P=0.0046. **(H)** Sholl analysis of IBA1^{+} microglia and Aβ plaques in 12-month-old mice. HIP: F(1.01,2.021)=51.41, P=0.0184. FC: F(1.044,2.089)=1182, P=0.0007. **(I)** Sholl analysis of TREM2^{+} microglia and Aβ oligomers in 12-month-old mice. F(1.434,2.868)=1551, P<0.0001. **(J)** Sholl analysis of TREM2^{+} microglia and Aβ plaques in 12-month-old mice. HIP: F(1.013,2.027)=59.88, P=0.0157. FC: F(1.007,2.015)=87.52, P=0.011. **(K)** Sholl analysis of TMEM119^{+} microglia and Aβ oligomers in 12-month-old mice. F(1.001,2.001)=5.275, P=0.1484. **(L)** Sholl analysis of TMEM119^{+} microglia and Aβ plaques in 12-month-old mice. HIP: F(1.143,2.287)=2.83, P=0.2237. FC: F(1.176,2.353)=2.066, P=0.2785. Columns represent the mean ± SD, statistical analysis was performed using one-way ANOVA. HIP, hippocampus. FC, frontal cortex. Each data point represents a mean from three technical replicates in one mouse.

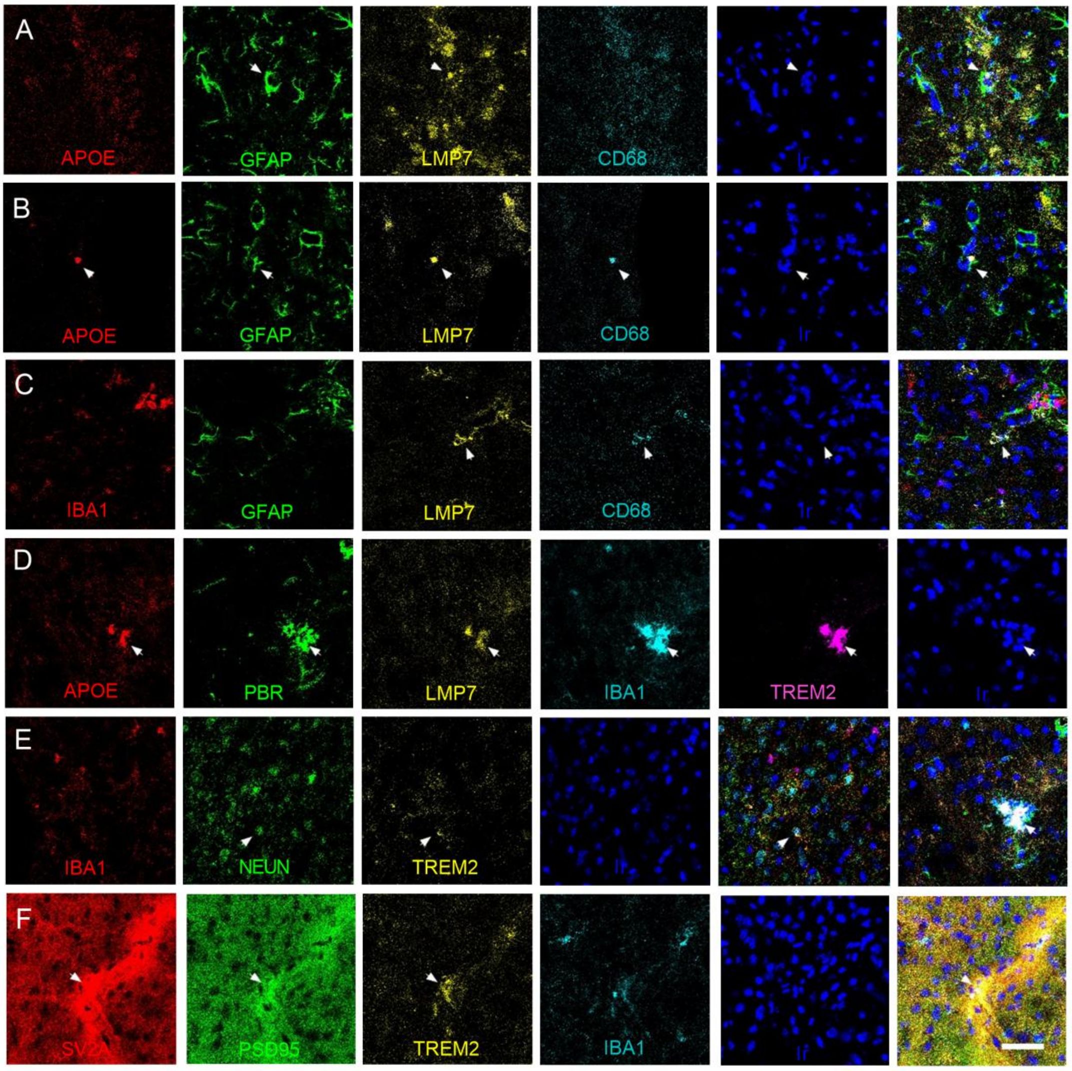

Supplementary Figure 10. Representative IMC staining images of glial phenotypic clusters. (A) GFAP^{+}APOE^{-}CD68^{-}LMP7^{+} activated astrocytes. **(B)** GFAP^{+}APOE^{+}CD68^{+}LMP7^{+} activated astrocytes. **(C)** CD68^{+}LMP7^{+}GFAP^{-}IBA1^{-} activated glial cells. **(D)** IBA1^{+}PBR^{+}APOE^{+}TREM2^{+}LMP7^{+} activated microglia. **(E)** NEUN^{+}TREM2^{+} cluster. **(F)** TREM2^{+}PSD95^{+}SV2A^{+} cluster. Scale bar = 50 µm.

Supplementary Table 1. **PCR reaction setup.**

Component	25 µl Reaction
Q5 High-Fidelity 2X Master Mix	12.5 µl
10 µM Forward Primer	1.25 µl
10 µM Reverse Primer	1.25 µl
Template DNA	Variable

Component	25 µl Reaction
Nuclease-Free Water	to 25 µl

Supplementary Table 2. PCR reaction thermocycling conditions.

Step	Temperature	Time
Initial Denaturation	98°C	30 seconds
30 Cycles	98°C	10 seconds
	52°C	30 seconds
	72°C	30 seconds/kb
Final Extension	72°C	2 minutes
Hold	4–10°C	-

Supplementary Table 3. Primary antibody selection for IHC staining.

Antigen	Host Species	Dilution	Kit	Source	DAB Incubation	RRID
IBA1	Rabbit	1:3000	SS	Wako D19-19741	70s	839504
GFAP	Rabbit	1:2000	SS	Dako Z0334	60s	10013382
CD16/32	Rat	1:1000	Ip	BD Biosciences 553141	3min	394656
CD163	Rabbit	1:1000	Ip	Bioss Bs-2527R	2min	10856166
TMEM119	Rabbit	1:4000	Ip	Abcam ab209064	2min	2800343
CD68	Rat	1:2000	Ip	BIO-RAD MCA1957GA	2min	324217
PBR	Rabbit	1:2000	Ip	Abcam ab109497	2min	10862345
6C3	Mouse	1:250	SS	Merck MABN254	4min	2895168
NAB61	Mouse	1:1000	SS	Dr. Virginia Lee from University of Pennsylvania	7min	N/A
NEUN	Mouse	1:2000	Ip	Merck MAB377	6min	177621
SV2A	Rabbit	1:2000	SS	Abcam ab32942	90s	778192
PSD95	Rabbit	1:2000	Ip	Abcam ab269863	30s	2895158

Supplementary Table 4. Primary antibody selection for IF staining.

Antigen	Host Species	Dilution	Source	Antigen Retrieval	Primary Incubation	RRID
IBA1	Rabbit	1:400	Wako D19-19741	No	2.5h	839504
IBA1	Goat	1:200	Abcam ab5076	10 min FA	overnight	2224402
GFAP	Rat	1:400	Invitrogen 13-0300	No	overnight	86543
CD16/32	Rat	1:200	BD Biosciences 553141	No	2.5h	394656
CD163	Rabbit	1:400	Bioss Bs-2527R	No	overnight	10856166
TMEM119	Rabbit	1:400	Abcam ab209064	No	2.5h	2800343
CD68	Rat	1:200	BIO-RAD MCA1957GA	No	2.5h	324217
PBR	Rabbit	1:200	Abcam ab109497	No	overnight	10862345

Supplementary Table 5. Secondary antibody selection for IF staining.

Antibody	Host Species	Dilution	Source	Fluorophore Conjugate	RRID
AlexaFluor anti-rabbit	Donkey	1:200	Invitrogen A-21206	488	2535792
AlexaFluor anti-goat	Donkey	1:200	Invitrogen A-11057	568	142581
AlexaFluor anti-rat	Donkey	1:200	Invitrogen A-21209	594	2535795

Supplementary Table 6. Primary antibody cocktail for IMC staining.

Antigen	Dilution	Source	Metal	RRID
IBA1	1:500	Wako D19-19741	169Tm	839504
GFAP	1:500	Dako Z0334	143Nd	10013382
CD16/32	1:50	BD Biosciences 553141	164Dy	394656
APOE	1:100	Abcam ab227993	146Nd	N/A
6C3	1:500	Merck MABN254	151Eu	2895168
NEUN	1:500	Merck MAB377	148Nd	177621
TREM2	1:300	R&D AF1729	161Dy	354956
PBR	1:500	Abcam ab213654	149Sm	10862345
SV2A	1:800	Abcam ab32942	171Yb	778192
PSD95	1:500	Abcam ab269863	170Er	2895158
NAB61	1:300	Dr. Virginia Lee from University of Pennsylvania	166Er	N/A
LMP7	1:300	Santa Cruz sc-365699	162Dy	10846323
MOC87	1:500	Abcam ab251335	174Yb	N/A
CD163	1:50	Bioss Bs-2527R	154Sm	10856166
TMEM119	1:100	Abcam ab209064	155Gd	2800343
CD68	1:100	BioLegend 137002	159Tb	2044004

Supplementary Table 7. Colocalization area coverage surrounding Aβ plaques or oligomers with Sholl analysis.

Marker	Aβ plaques				Aβ oligomers			
	2.5 months		12 months		2.5 months		12 months	
	FC	HIP	FC	HIP	FC	HIP	FC	HIP
IBA1	4.3±2.8%	2.5±2.2%	15.6±3.7%	17.1±7.0%	7.0±4.1%	6.9±4.2%	17.9±4.7%	20.7±7.7%
TREM2	1.3±0.8%	1.4±1.0%	11.0±4.4%	10.3±4.5%	2.9±2.3%	5.7±4.1%	12.8±5.0%	12.4±5.7%
TMEM119	1.8±2.1%	1.2±1.5%	1.8±1.0%	1.0±0.7%	6.0±5.5%	8.5±5.8%	2.3±1.5%	1.3±0.9%
CD16/32	-	-	1.0±0.8%	1.1±0.8%	-	-	1.5±1.3%	1.8±1.3%
APOE	-	-	4.5±2.2%	4.6±3.9%	-	-	5.7±3.0%	7.0±5.1%
LMP7	-	-	3.5±1.4%	2.9±1.3%	-	-	4.7±2.0%	4.1±1.8%
CD68	-	-	1.2±0.7%	0.7±0.5%	-	-	1.4±1.0%	1.1±1.0%
CD163	-	-	2.3±1.2%	1.1±1.0%	-	-	3.0±1.8%	2.0±1.8%
GFAP	1.7±2.1%	3.2±2.2%	6.3±2.2%	6.7±1.9%	1.3±1.3%	4.6±2.4%	6.6±2.7%	7.0±2.5%
PBR	1.5±0.9%	1.1±0.8%	8.3±3.0%	9.7±3.5%	1.2±0.7%	1.5±1.2%	9.1±3.2%	10.2±3.6%
NEUN	5.6±5.1%	4.0±3.0%	6.7±3.7%	4.6±3.5%	14.6±11.4%	21.8±8.4%	8.9±4.7%	5.4±3.8%
SV2A	50.2±11.3%	49.7±14.0%	42.0±12.5%	39.8±11.8%	56.8±12.7%	56.3±22.7%	45.1±11.4%	43.2±14.0%
PSD95	26.9±7.5%	39.4±12.7%	23.0±5.2%	33.7±8.8%	33.3±8.6%	45.6±18.9%	24.6±7.3%	32.8±11.5%
NAB61 (Aβ oligomers)	15.8±8.0%	10.2±7.8%	20.0±6.8%	22.4±7.6%	-	-	-	-

Supplementary Table 8. R value summary of correlation plots.

		12 months old		2.5 months old	
Variable I	Variable II	Frontal Cortex	Hippocampus	Frontal Cortex	Hippocampus
6C3	GFAP	0.175****	0.096****	0.089****	0.021*
6C3	IBA1	0.390****	0.374****	0.152****	0.284****
6C3	CD16/32	0.100****	0.123****	-	-
6C3	CD163	0.086****	0.100****	-	-
6C3	CD68	0.124****	0.131****	-	-
6C3	APOE	0.462****	0.389****	-	-
6C3	LMP7	0.197****	0.155****	-	-
6C3	PBR	0.296****	0.263****	0.035****	0.037****
6C3	TMEM119	0.103****	0.085****	0.172****	0.429****
6C3	TREM2	0.456****	0.470****	0.122****	0.244****
NAB61	GFAP	0.221****	0.134****	0.096****	0.045****
NAB61	IBA1	0.440****	0.470****	0.668****	0.729****
NAB61	CD16/32	0.443****	0.410****	-	-
NAB61	CD163	0.475****	0.355****	-	-
NAB61	CD68	0.465****	0.319****	-	-
NAB61	APOE	0.547****	0.436****	-	-
NAB61	LMP7	0.509****	0.321****	-	-
NAB61	PBR	0.250****	0.220****	0.059****	0.104****
NAB61	TMEM119	0.536****	0.416****	0.795****	0.823****
NAB61	TREM2	0.438****	0.465****	0.609****	0.725****
NAB61	6C3	0.696****	0.732****	0.331****	0.414****
GFAP	APOE	0.255****	0.200****	-	-
GFAP	CD163	0.111****	0.071****	-	-
GFAP	LMP7	0.103****	0.108****	-	-
GFAP	PBR	0.341****	0.258****	0.254****	0.307****
IBA1	PBR	0.517****	0.459****	0.097****	0.148****
IBA1	TREM2	0.667****	0.638****	0.768****	0.738****
IBA1	CD163	0.234****	0.212****	-	-
IBA1	CD16/32	0.277****	0.279****	-	-
IBA1	APOE	0.496****	0.387****	-	-
IBA1	LMP7	0.445****	0.355****	-	-
IBA1	CD68	0.298****	0.251****	-	-

$|R|>0.5$ is strong correlation. $0.3<|R|<0.5$ is moderate correlation. $0.1<|R|<0.3$ is weak correlation. **** $p<0.0001$, *** $p<0.001$, ** $p<0.01$, * $p<0.05$.

Supplementary File: Code for Sholl analysis in ImageJ.

```
Stack.setXUnit("um");
run("Properties...", "channels=1 slices=1 frames=1 pixel_width=1.0000 pixel_height=1.0000 voxel_depth=25400.0508");

getVoxelSize(px, py, pz, unit);

//Dialog.create("Options");
//Dialog.addNumber("Enter numbr of required rings", 3);
//Dialog.addNumber("Enter thickness of rings ("+unit+")", 30);
//Dialog.show();

number_of_rings=3;
increment=30;

//get original ROIs and combine
n=roiManager("count");
orig_rois=Array.getSequence(n);
roiManager("select", orig_rois);
roiManager("Combine");
roiManager("Add");

//remove riginal ROIs
//roiManager("select", orig_rois);
//roiManager("delete");

for (ring=0;ring<number_of_rings;ring++){

//create enlargements
roiManager("select", n+ring);
roiManager("rename", "Region "+ring);
run("Enlarge...", "enlarge="+increment);
roiManager("Add");
}
roiManager("select", n+ring);
roiManager("rename", "Region "+ring);

//create rings
for (ring=0;ring<number_of_rings;ring++){
roiManager("Select", newArray(n+ring,n+ring+1));
roiManager("XOR");
roiManager("Add");
}
```

```
roiManager("deselect");
roiManager("Set Fill Color", "#4d00ff00");
//label rings
for (ring=0;ring<number_of_rings;ring++){
roiManager("select", n+number_of_rings+ring+1);
roiManager("rename", "Ring "+ring+1);
}

roiManager("select", orig_rois);
RoiManager.setPosition(0);
roiManager("Set Color", "white");
roiManager("Set Line Width", 0);

for (ring=0;ring<number_of_rings;ring++){
roiManager("select", n+ring);
RoiManager.setPosition(0);
roiManager("Set Color", "white");
roiManager("Set Line Width", 0);
}

for (ring=0;ring<number_of_rings;ring++){
roiManager("Select", newArray(n+ring,n+ring+1));
RoiManager.setPosition(0);
roiManager("Set Color", "white");
roiManager("Set Line Width", 0);
}

for (ring=0;ring<number_of_rings;ring++){
roiManager("select", n+number_of_rings+ring+1);
RoiManager.setPosition(0);
roiManager("Set Color", "white");
roiManager("Set Line Width", 0);
}

saveAs("Tiff", "");
roiManager("Select", 1);
run("Select All");
roiManager("Deselect");
roiManager("Delete");
close();
```
