# Supplementary material for: Associations of amyloid-β oligomers and plaques with neuropathology in the AppNL-G-F mouse
Source: Brain Commun. 2024 Jun 25;6(4):fcae218. doi: 10.1093/braincomms/fcae218 (PMC11258573; doi:10.1093/braincomms/fcae218)
Supplement: fcae218_Supplementary_Data [file Original_submission_decision_letter.pdf › Original_submission_decision_letter.pdf]

From: brcoms.editorialoffice@oup.com
To:
CC:
BCC:
Subject: Brain Communications - Decision on Manuscript ID BRAINCOM-2023-360
Body: 04-Oct-2023

Dear Dr. Matthews,

Thank you for your recent submission BRAINCOM-2023-360 entitled "Neuropathology Associated with Amyloid-β Oligomers in the AppNL-G-F Mouse" to Brain Communications.

Your manuscript has been reviewed, and while the reviewers and editorial team appreciate many aspects of the paper, they raise some concerns that you will need to address in order for the paper to be considered for publication.

If you can address these concerns, I invite you to submit a revised manuscript and a response to each of the reviewers' comments. The reviewer(s)' comments are included at the bottom of this message.

In addition to the reviewers' comments, please also address our Scientific Editor's comments added below to comply with our requirements for presentation of scientific data at Brain Communications.

Please format your revised manuscript according to our Instructions to Authors https://academic.oup.com/braincomms/pages/General_Instructions
A summary of key points is attached to help with this. Please note that there can be substantial delays to publication if you don't resubmit in line with our requirements.

To revise your manuscript, click the link below or log into https://mc.manuscriptcentral.com/braincom and enter your Author Centre, where you will find your manuscript title listed under "Manuscripts with Decisions." Under "Actions," click on "Create a Revision." Your manuscript number will be appended to denote a revision.

*** PLEASE NOTE: This is a two-step process. After clicking on the link, you will be directed to a webpage to confirm. ***

https://mc.manuscriptcentral.com/braincom?URL_MASK=54c3a0afba774943b37f3d2bfd584c32

Please upload a revised manuscript with changes tracked with track changes or in coloured text.

When submitting your revised manuscript, you will be asked to respond to the reviewer(s) comments. Please be as specific as possible in your response to all of the comments.

The editorial team at Brain Communications aims to increase transparency in scientific publishing and to demonstrate the value that peer review brings to the scientific process. Please therefore consider whether you are willing to have reviewer(s)' comments on your paper and your response published as supplementary data with the paper if it is accepted. This is entirely optional and will only be done if both the authors and reviewers agree. While submitting your revision there will be an opportunity to review or update the answer you gave previously, if necessary.

IMPORTANT: Your original files are available to you when you upload your revised manuscript. Please delete any redundant files before completing the submission.

Because we are trying to facilitate timely publication of manuscripts submitted to Brain Communications, we would appreciate your revised manuscript as soon as possible. If it is not possible for you to resubmit within 3 months, the option to submit a revision will expire, and we would then consider your revised paper as a new submission. However, if you need a little more time, please get in touch and we can extend this deadline for you.

Once again, thank you for submitting your manuscript to Brain Communications and I look forward to receiving your revision.

Best wishes

Professor Tara Spires-Jones
Founding Editor, Brain Communications

Reviewer(s)' Comments to Author:

Reviewer: 1

Comments to the Author
In this study, Tang et al. demonstrate, using a combination of transgenic mouse models, an interesting difference in the properties of Aβ oligomers when associated with plaques ('APP NL-G-F') as opposed to non-plaque associated Aβ ('APP-Hu'). Experimental outcome measures include descriptive analyses of classical glial and synaptic markers implicated in reactive gliosis and synapse loss, respectively—both of which represent highly disease-relevant processes in amyloid-associated neuropathies, such as Alzheimer's Disease (AD).

The idea of Aβ oligomers differing in their toxic properties on the basis of their aggregation state represents a novel and timely contribution to the field that complements work investigating the relationship between proto-fibrillar conformation and assembly state in mediating toxicity (Vadukul et al., 2020; FEBS Letters). Furthermore, the study submitted here holds potential translational utility in highlighting Aβ oligomer functional heterogeneity, which may inform on oligomer-targeting therapies—particularly in light of the recent FDA approval of proto-fibril targeting Lecanemab for the treatment of patients presenting with mild cognitive impairment (MCI).

Overall, although this study does demonstrate potentially interesting findings, there appears to be shortcomings in the collection and presentation of data that make said data difficult to interpret and, on a wider scale, generalisable. These are highlighted below in the form of bullet-points.

Methods:

- Was there a technical reason as to why images analysed in Ilastik were saved in Jpeg format? This involves lossy compression and important data will be lost from the raw images.

- How did the authors train Ilastik? What was the sample size used for training and was there any QC done to confirm predictive accuracy?

- For statistical analyses, the authors have not mentioned what post-hoc test was used to provide the pairwise comparisons shown in the graphs.

- The authors have not clarified why some experiments have n = 6 and others have n = 3? Was there a technical reason for this?

- For statistical analyses, an n = 3 is the minimum required for residual variance in running linear statistical models (e.g. ANOVA). Without knowing the distribution of the data, or seeing data points in graphs, it is unclear whether the ANOVA output can be interpreted with confidence.

- Related to the above, did the authors check the data distribution? One-way ANOVA carries a distributional assumption of data falling within a normal distribution. If the authors did check data distribution, this should be included in the statistical methods section.

- Likewise, Pearson correlations also carry a similar assumption — was the data normally distributed? If not, a non-parametric Spearman correlation would better fit the data.

- In experiments where n = 3, all mice are male. This importantly misses potential sex-dependent effects that hold great relevance when assessing diseases that present with significant sexual dimorphism (e.g. Alzheimer's Disease, which occurs more commonly in females).

- Was there justification for using just the right hemisphere for the study?

- Was there a technical reason for incubating some antibodies overnight and others for 2.5 hours for IF staining? (Supplementary Table 4).

- It may also be helpful to include traceable RRID codes for each antibody to the tables.

Results:

General (Overall) Comments:

- The presentation of figures could be improved (see below).

- In terms of general presentation, some figures lack cohesiveness and are difficult to read/interpret. For example, the layout within Figure 5 alone is inconsistent, with Figure 5A-B being side-by-side, and C-D being top-bottom. Comparing between figures, the presentation format is also generally inconsistent. Ideally, the authors should format figures in a similar manner throughout to aid in reading.

- For representative images, DAB staining should be similarly white-balanced to allow for easier visual comparison. This appears inconsistent across the manuscript, as Figure 4A, C, E, G and Figure 5 A, C show a general balance (although there is some slight difference) while Figure 1 A, B shows poor balancing.

- Critically, none of the graphs include datapoints. These should always be included as, at current, it is impossible for the reader to ascertain what the distributional properties of the data are. Data points for each sample should be represented as either mean/median based on the averaging of technical replicates within biological samples.

- Ideally, graphs showing similar types of data should be consistent in format throughout. It is unclear why some graphs are represented as bar charts while others are violin plots. Violin plots can help the reader to visually assess the distribution of the data; however, without data points, this is difficult to interpret alone. Overall, the data presented here should be consistently shown throughout—either as bar charts, violin plots or box-whisker plots.

- Could the authors clarify what is being plotted in the violin plots (e.g. Figure 3B, C) in terms of data points? The sample size is reported as n = 3 mice; however, shape of the plots suggests more than 3 data points?

- Throughout the manuscript, the authors refer to the hippocampus quite generally. Was quantification carried out on the whole hippocampus or stratified by anatomical sub-region? If the former is the case, did the authors account for e.g. differences in baseline cell count in regions such as e.g. the dentate gyrus, which will present with higher cell body density?

Figure 1:

- Fig 1 A: it would be good to include representative images from all three age time-points (2.5, 7 & 12 m.o) to demonstrate the timescale of plaque growth vs. oligomer accumulation. Especially as this forms the key focus of data presented in associated Fig 1B.

- Fig 1 C, D: can the authors clarify why they only took measures over the lifespan from WT and NLGF mice? Why not also include the APPHu model, as this is included later on in the 12 month-old mice?

Figure 2:

- Fig 2 A: I'm unsure whether optical density from paraffin staining is convincing as a method for assessing synapse density. Due to light diffraction with tissue depth, optical density is likely to be heavily influenced by potential confounders such as tissue thickness and staining quality. While likely sufficient for larger structures, such as NeuN for neuronal nuclei, it is likely

that any optical density measures for small structures, such as synapses, are only reliable up to a certain depth before light is scattered.

- Limitations of this technique are particularly highlighted by the representative image shown for PSD-95, which appears to show wide-spread diffuse staining with no clearly defined puncta. I think a technique with higher resolving limit is required to quantify synaptic density with greater confidence.

- What region are the representative images taken from? Are they hippocampus or frontal cortex?

- Fig 2 D-E: the figure legend suggests a 2-way ANOVA was used for all analysis although the data looks suited to a 1-way ANOVA?

- It would be useful to include the hippocampal lifespan data from Supplementary Fig. 2A in the main figure. It also may be useful to have representative images from each age time-point.

Figure 3:

- Please see previous comments e.g. about violin plots.

Figure 4:

- Can the authors clarify why APPHu mice were not included for microglial analysis in Figure 4? These mice are included for astrocyte analysis later in Figure 5? Supplementary Figure 3 shows frontal cortex IBA1 data for APPHu mice but this is not in the main figure.

Figure 5:

- Fig 5A: which genotype is this representative image taken from and what brain region? Ideally, it would be good to show both regions (frontal cortex + hippocampus) and both genotypes being compared (WT vs. NL-G-F).

- Fig 5D: for reliably measuring parameters such as process length, 3D analysis would be strongly encouraged. At the moment, measures taken from 2D images are not entirely convincing — how are overlapping fragments of processes distinguished from one-another? Furthermore, some processes may move in/out of the z-plane and appear fragmented.

- I think the frontal cortex data from supplementary Figure 3 F-G should be included here— especially as Fig. 5B is illustrating data from this region.

Figure 6:

- Fig 6A-B: were the results from Pearson correlation found to be significant? The correlation plots don't include p-values.

Figure 7:

- Increasing general text size throughout this figure would aid in legibility — it is difficult to read the names for each cluster at the current size.

- The data for younger (2.5 month old mice) is shown in Fig 7 C-D, while older mice (12 months old) are shown earlier in Fig 7 A-B. The figure may flow better by presenting data chronologically, with younger mice first, followed by older, or alternatively having 2.5 vs 12 month old t-SNE plots side-by-side, so the reader can more easily visually identify the age-associated increase in clusters reported.

Supplementary Figures

General (Overall) Comments:

- Some figures lack sub-categories, making them difficult to interpret e.g. Figures S2-S3 lack 'A' and 'B' labelling, although this is mentioned in the figure legends.

- Figure S4 should ideally have the markers labelled within the images themselves, in a similar fashion to what was presented in Figure 9.

Reviewer: 2

Comments to the Author

This study investigates the relationship between Aβ oligomers, plaques, glial activation, and neurodegeneration markers in AppNL-G-F and Apphu mouse models of Alzheimer's disease (AD) using immunohistochemistry and imaging mass cytometry (IMC). The Apphu mice, which have abundant Aβ oligomers but lack plaques, show astrocyte activation without microglial activation or synaptic/neuronal pathology. In contrast, the AppNL-G-F mice exhibit high levels of Aβ oligomers and plaques, accompanied by significant astrocytic and microglial activation. The increase in Aβ oligomers over time in Apphu mice correlates more with astrocytic activation. Spatial analysis indicates a closer association between activated microglia and Aβ oligomers rather than plaques in AppNL-G-F mice.

The novelty of the work lies in using an Aβ oligomer-specific antibody to map and characterize the accumulation of Aβ oligomers in these relatively new models and identifying spatial relationships using IMC.

Overall, the findings are significant, but the article may benefit from addressing the following concerns:

Major concerns:

1-The article's main conclusion is somewhat ambiguous. The abstract mentions the dependency of glial cell activation on the aggregation state of Aβ, supported only by correlations drawn from immunohistochemistry findings across different models. However,

spatial analysis indicates that in AppNL-G-F mice, microglia (and not astroglia) are more associated with oligomers. The authors might consider refining and reflecting their conclusion in the abstract and the title for clarity and accuracy.
.
2- The results solely depend on the assumption that NAB61 identifies oligomers. While this was shown in previous articles using this particular antibody, it is known that there are many different kinds of Aβ aggregation intermediates. The results could be antibody-dependent if the antibody is selective to one specific intermediate. The authors discussed this limitation, but the study findings will be stronger if any of the experimental findings can be replicated using a different oligomeric Aβ antibody.

3- there needs to be a more comprehensive discussion regarding the absence of a correlation between astrocytic markers and Aβ oligomers in the AppNL-G-F model.

Minor Comments:
In the abstract, consider replacing "expression" of Aβ oligomers with "levels." Also, the term "Human Aβ proteins" might be confusing, and an alternative like "Aβ aggregates" might convey the concept more clearly.

Oligomers and plaque are formed and not expressed.
Similarly, the last sentence in the abstract is not entirely accurate. "..depends on nature and aggregation state of Aβ oligomeric species expressed". Aβ peptide aggregation states include dimers, Aβ oligomers, Aβ protofibrils, and Aβ plaque. So this sentence can be replaced with "depends on nature and aggregation state of Aβ peptides."

Please add appropriate labels (6C3 or NAB61) to the figures in panels A and B of Figure 2.

For Figure 2, panel B, clarify whether the statistics were conducted using the number of animals or images/experiments. Also,

Throughout the manuscript, it would be beneficial if the figures displayed the number of animals to illustrate variability between subjects better.

The authors should focus on discussing the significance and limitations of the main conclusions.

Comments from the Scientific Editor:

-Please supply a graphical abstract. A graphical abstract is a visual representation of the central finding or methodology of the paper. It will be displayed in the table of contents. Authors should strive to make the graphical abstract informative, interesting, visually appealing, and straightforward (no caption needed, no abbreviations except gene names). The technical requirements for the graphical abstracts are:
- Size: 120 x 120 mm (300-600dpi) or 3000 x 3000 pixels
- Font: Arial 12-16 pt
- File type: tiff or PDF

- Aspect ratio: 1:1 preferred

-Please add an Abbreviated Summary (up to 50 words) of your paper that captures the main purpose and conclusions of the work. The summary should state the main results written in the third person ('[1st author surname] et al. report that ...') and conclusion. This summary will be used in the contents list online and should not contain any abbreviations other than those on the accepted abbreviations list and accepted gene/protein names, making it accessible to the non-specialist. Please upload it as a Word document. Allowed abbreviations: AIDS; ANOVA; AMPA; ATP; A,T,C,G; CNS; CSF; CT; DNA; ECG; EEG; EMG; GABA; HIV; MRI; NMDA; PET; PCR; RNA.

- Please include the appropriate checklist to comply with our ethical standards (see Reporting Guideline section within 'Instructions to authors').

-Please include a paragraph about statistical analysis in the 'Materials and Methods' section.

-for each statistical analysis in a figure, please report in the figure legend, where missing the test used and relevant test statistic values alongside p values (for example F or t values for ANOVAs and t-tests). Also, please, provide details of the experimental unit for each group analysed in these plots, and if the number is <10 show the single data-points.   Please note: We do not accept analyses where individual measurements are the experimental unit when there is likely to be biological variability between animals, culture replicates, etc. For this type of data, we require either that a mean or median is taken per animal/culture replicate followed by statistical analyses, or that more sophisticated analyses are performed such as mixed effects modelling which can account for random effects.
