# Supplementary material for: Associations of amyloid-β oligomers and plaques with neuropathology in the AppNL-G-F mouse
Source: Brain Commun. 2024 Jun 25;6(4):fcae218. doi: 10.1093/braincomms/fcae218 (PMC11258573; doi:10.1093/braincomms/fcae218)
Supplement: fcae218_Supplementary_Data [file Original_submission_Response_to_reviewers_comments.pdf › Original_submission_Response_to_reviewers_comments.pdf]

We are grateful to the reviewers and have responded to points raised as below.

Reviewer: 1

Comments to the Author
In this study, Tang et al. demonstrate, using a combination of transgenic mouse models, an interesting difference in the properties of Aβ oligomers when associated with plaques (‘APP NL-G-F’) as opposed to non-plaque associated Aβ (‘APP-Hu’). Experimental outcome measures include descriptive analyses of classical glial and synaptic markers implicated in reactive gliosis and synapse loss, respectively—both of which represent highly disease-relevant processes in amyloid-associated neuropathies, such as Alzheimer’s Disease (AD).

The idea of Aβ oligomers differing in their toxic properties on the basis of their aggregation state represents a novel and timely contribution to the field that complements work investigating the relationship between proto-fibrillar conformation and assembly state in mediating toxicity (Vadukul et al., 2020; FEBS Letters). Furthermore, the study submitted here holds potential translational utility in highlighting Aβ oligomer functional heterogeneity, which may inform on oligomer-targeting therapies—particularly in light of the recent FDA approval of proto-fibril targeting Lecanemab for the treatment of patients presenting with mild cognitive impairment (MCI).

Overall, although this study does demonstrate potentially interesting findings, there appears to be shortcomings in the collection and presentation of data that make said data difficult to interpret and, on a wider scale, generalisable. These are highlighted below in the form of bullet-points.

Methods:

- Was there a technical reason as to why images analysed in Ilastik were saved in Jpeg format? This involves lossy compression and important data will be lost from the raw images.

We thank the reviewer for raising this important point. Yes, there is a technical reason. The tiff format can cause errors when processing with Ilastik. We found the jpeg format more reliable. The resolution of IMC image is 1 pixel, so we did meaningful raw data in this format. We have tried to clarify this in the revised manuscript.

- How did the authors train Ilastik? What was the sample size used for training and was there any QC done to confirm predictive accuracy?

We have now included the following line regarding training in the methods:

“We used ~2500 contiguous pixels (50 × 50 pixels matrix) for training for on each brain section, applying the following feature selection parameters: color/intensity, 10; edge, 10; texture, 10.”

QC was performed using automated tools available in Ilastik, which was carried out after pixel segmentation.

- For statistical analyses, the authors have not mentioned what post-hoc test was used to provide the pairwise comparisons shown in the graphs.

We thank the reviewer for raising this important point. We have now included the following line in the methods:

“The Tukey test as implemented in GraphPad Prism 8.4 software was used.”

- The authors have not clarified why some experiments have n = 6 and others have n = 3? Was there a technical reason for this?

We apologize for not making this clear. At the start, we initially used 6 mice in each age group (3 male and 3 female) to compare $App^{NL\text{-}G\text{-}F}$ and WT mice. With these we compared pathology between sexes. As we found no differences in these measures, we proceeded using only 3 male mice for subsequent observations to minimize animal numbers used.

- For statistical analyses, an n = 3 is the minimum required for residual variance in running linear statistical models (e.g. ANOVA). Without knowing the distribution of the data, or seeing data points in graphs, it is unclear whether the ANOVA output can be interpreted with confidence.

We thank the reviewer for raising this important point. We have redrawn figures to represent all data points in the revised manuscript.

- Related to the above, did the authors check the data distribution? One-way ANOVA carries a distributional assumption of data falling within a normal distribution. If the authors did check data distribution, this should be included in the statistical methods section.

We thank the reviewer for raising this important point. We have now included the following line in the methods:

“Data were tested for normality with a Shapiro-Wilk test.”

- Likewise, Pearson correlations also carry a similar assumption — was the data

normally distributed? If not, a non-parametric Spearman correlation would better fit the data.

We thank the reviewer for raising this important point. We did check data for normality. We have now added this information to the manuscript with descriptions of the Pearson correlations.

- In experiments where n = 3, all mice are male. This importantly misses potential sex-dependent effects that hold great relevance when assessing diseases that present with significant sexual dimorphism (e.g. Alzheimer's Disease, which occurs more commonly in females).

We agree with the reviewer that Alzheimer's Disease may show sex differences and that these could be manifest in this β-amyloid KI model. Our research started by using 6 mice (3 male and 3 female), and we did not see a major effect between sex with Aβ and glial and neuronal markers (data not shown). We then proceeded with male mice only to explore for major effects in this specific study. We acknowledge that sex specific effects in microglial numbers were suggested in a previous report that explored 6 mice of each sex at 3 and 6 months of age (PMID: 34831483), but note that this claim was not supported by statistical analyses and is not obvious from the data presented.

- Was there justification for using just the right hemisphere for the study?

As there is no evidence of hemispheric asymmetry in App^{NL-G-F} mouse brain pathology reported to date, we assumed symmetry and only used one hemisphere for this research. The other hemisphere was used for another set of experiments to minimize animal use.

- Was there a technical reason for incubating some antibodies overnight and others for 2.5 hours for IF staining? (Supplementary Table 4).

We tested various incubation times from 1 hour to overnight, as well as different incubation temperature (RT or 4°C), to optimize signal to noise ratio. The incubation times indicated in supplementary tables was the optimized protocol to show the best results.

"The incubation conditions were optimized for the best signal to noise ratio in each case."

- It may also be helpful to include traceable RRID codes for each antibody to the tables.

We thank the reviewer for raising this important point. We have now included the

RRID codes to the antibody tables.

Results:

General (Overall) Comments:

- The presentation of figures could be improved (see below).

- In terms of general presentation, some figures lack cohesiveness and are difficult to read/interpret. For example, the layout within Figure 5 alone is inconsistent, with Figure 5A-B being side-by-side, and C-D being top-bottom. Comparing between figures, the presentation format is also generally inconsistent. Ideally, the authors should format figures in a similar manner throughout to aid in reading.

We thank the reviewer for raising this important point. However, Figure 5AB shows CD163, which were very rare in the WT mouse brains so we only showed a representative image from an App^{NL-G-F} mouse brain. Figure 5CD shows GFAP, which we detected in both App^{NL-G-F} and WT mice, although there were clear difference between the two genotypes. For this reason we chose to show representative images from both genotypes and use a different layout. The comparative images between different genotypes were shown in Supplementary Figure 5 and 6 for all other glial markers.

- For representative images, DAB staining should be similarly white-balanced to allow for easier visual comparison. This appears inconsistent across the manuscript, as Figure 4A, C, E, G and Figure 5 A, C show a general balance (although there is some slight difference) while Figure 1 A, B shows poor balancing.

We thank the reviewer for raising this important point. We have adjusted the white-balance in the new manuscript.

- Critically, none of the graphs include datapoints. These should always be included as, at current, it is impossible for the reader to ascertain what the distributional properties of the data are. Data points for each sample should be represented as either mean/median based on the averaging of technical replicates within biological samples.

We thank the reviewer for raising this important point. We have represented all data points in the new manuscript.

- Ideally, graphs showing similar types of data should be consistent in format throughout. It is unclear why some graphs are represented as bar charts while others are violin plots. Violin plots can help the reader to visually assess the distribution of the data; however, without data points, this is difficult to interpret alone. Overall, the

data presented here should be consistently shown throughout—either as bar charts, violin plots or box-whisker plots.

We apologize for inconsistent graph format. We have changed all to bar charts in the revised manuscript.

- Could the authors clarify what is being plotted in the violin plots (e.g. Figure 3B, C) in terms of data points? The sample size is reported as n = 3 mice; however, shape of the plots suggests more than 3 data points?

We apologize for using an improper representation. The violin plots were plotted with all 27 technical replicates (3 brain sections in each mice, and 3 ablation regions in each brain sections). We have corrected this in the revised manuscript.

- Throughout the manuscript, the authors refer to the hippocampus quite generally. Was quantification carried out on the whole hippocampus or stratified by anatomical sub-region? If the former is the case, did the authors account for e.g. differences in baseline cell count in regions such as e.g. the dentate gyrus, which will present with higher cell body density?

We thank the reviewer for raising this important point. The quantification was carried out on the whole hippocampus. We agree with the reviewer that the cell density could be different with anatomical sub-regions, but we only calculated the cell density with the whole region.

Figure 1:

- Fig 1 A: it would be good to include representative images from all three age time-points (2.5, 7 & 12 m.o) to demonstrate the timescale of plaque growth vs. oligomer accumulation. Especially as this forms the key focus of data presented in associated Fig 1B.

- Fig 1 C, D: can the authors clarify why they only took measures over the lifespan from WT and NLGF mice? Why not also include the APPHu model, as this is included later on in the 12 month-old mice?

We apologize for not measuring over the lifespan with App^{hu} model. Initially we were not expecting differences relative to the WT based on a report from the group that generated the mouse. We have now included the following line in the discussion:

"Future work may benefit from more detailed measures over the lifespan with App^{hu} mice."

Figure 2:

- Fig 2 A: I'm unsure whether optical density from paraffin staining is convincing as a method for assessing synapse density. Due to light diffraction with tissue depth, optical density is likely to be heavily influenced by potential confounders such as tissue thickness and staining quality. While likely sufficient for larger structures, such as NeuN for neuronal nuclei, it is likely that any optical density measures for small structures, such as synapses, are only reliable up to a certain depth before light is scattered.

- Limitations of this technique are particularly highlighted by the representative image shown for PSD-95, which appears to show wide-spread diffuse staining with no clearly defined puncta. I think a technique with higher resolving limit is required to quantify synaptic density with greater confidence.

We thank the reviewer for raising this important point. Just to clarify, we were using fixed frozen tissue, instead of paraffin tissue, as described in method section. We agree that there are multiple confounds to accurate determination of synapse density from immunohistochemistry at lower resolutions. In our estimations based on immunostaining optical density, we worked to minimize the impact of some of the confounds by using a consistent tissue section thickness, carrying out the staining with all samples at the same time to reduce batch effect of staining quality and performing three replicates for all brains. We have now included the following line in the methods:

"For the staining with SV2A and PSD95, all samples were carried out at the same time to reduce optical density batch effects."

- What region are the representative images taken from? Are they hippocampus or frontal cortex?

The SV2A and PSD95 images were taken from hippocampus, and NEUN image was taken from frontal cortex. Different brain regions were chosen for the representative images to interpret the markers in a better way, since SV2A and PSD95 showed better contrast in hippocampus, while NEUN was expressed more in frontal cortex. We analyzed all markers in both hippocampus and frontal cortex.

- Fig 2 D-E: the figure legend suggests a 2-way ANOVA was used for all analysis although the data looks suited to a 1-way ANOVA?

We apologize for this mistake. We have corrected this information and checked for all figures to avoid similar mistakes in the revised manuscript.

- It would be useful to include the hippocampal lifespan data from Supplementary Fig. 1A in the main figure. It also may be useful to have representative images from

each age time-point.

We thank the reviewer for raising this important point. The hippocampal data have been included in the main figure in the new manuscript. Representative images are only shown in 7-month WT mice. Differences in appearance across timepoint are not easily visually apparent at this resolution.

Figure 3:

- Please see previous comments e.g. about violin plots.

Figure 4:

- Can the authors clarify why APPHu mice were not included for microglial analysis in Figure 4? These mice are included for astrocyte analysis later in Figure 5? Supplementary Figure 3 shows frontal cortex IBA1 data for APPHu mice but this is not in the main figure.

We thank the reviewer for raising this important point. We have re-arranged the figures and included App^{hu} mice in Figure 4 in the new manuscript.

Figure 5:

- Fig 5A: which genotype is this representative image taken from and what brain region? Ideally, it would be good to show both regions (frontal cortex + hippocampus) and both genotypes being compared (WT vs. NL-G-F).

We thank the reviewer for raising this important point. The representative image is taken from frontal cortex in 12-month-old $App^{NL\text{-}G\text{-}F}$ mice, and we have now included this information in the figure legend. As CD163 shows little expression in WT mice, we only showed a representative image from $App^{NL\text{-}G\text{-}F}$ mice.

- Fig 5D: for reliably measuring parameters such as process length, 3D analysis would be strongly encouraged. At the moment, measures taken from 2D images are not entirely convincing — how are overlapping fragments of processes distinguished from one-another? Furthermore, some processes may move in/out of the z-plane and appear fragmented.

We apologize for this limitation. We tried to maximize our data reliability by using HALO to measure the process area, as well as process length. Microglial processes covered an area with a diameter of approximately 50 µm in the mouse with microglia soma being approximately 10 µm in diameter. However, as the processes of different microglia can overlap (in ways not always able to be resolved by 3D analyses), only 10 µm around the cell soma has been defined to measure process area/length. We

have attached an image here to show our image analysis strategy, and we have included the following line in the methods:

"Process length and process area were measured within 10 μm around the cell soma."

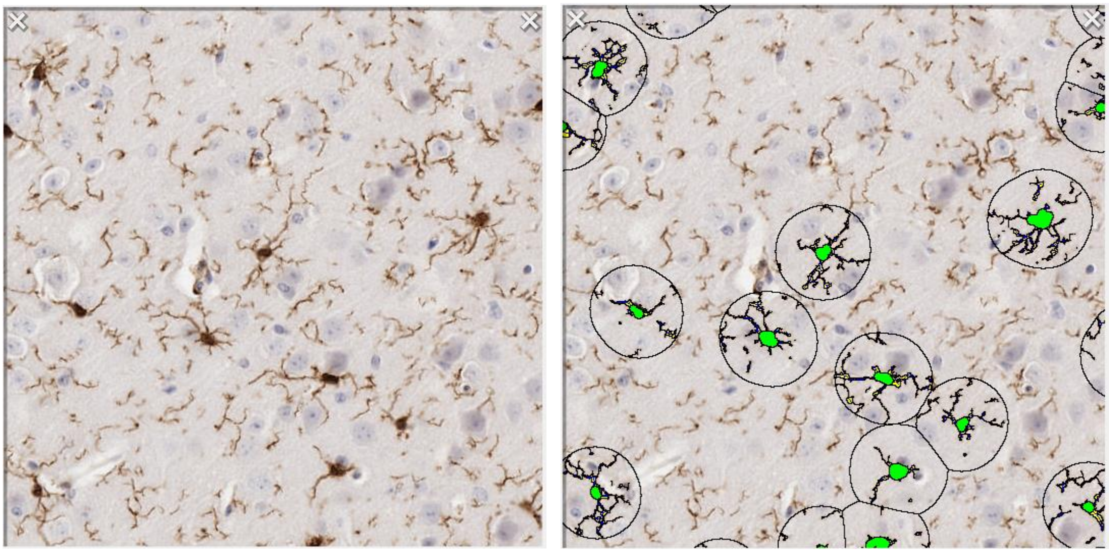

- I think the frontal cortex data from supplementary Figure 3 F-G should be included here—especially as Fig. 5B is illustrating data from this region.

We thank the reviewer for raising this important point. We have added the frontal cortex data to Figure 5 in the new manuscript.

Figure 6:

- Fig 6A-B: were the results from Pearson correlation found to be significant? The correlation plots don't include p-values.

Yes, the results are significant. The p-values are shown in Supplementary Table 8. We have added this information in Fig 6 legend in the revised manuscript.

Figure 7:

- Increasing general text size throughout this figure would aid in legibility — it is difficult to read the names for each cluster at the current size.

We thank the reviewer for raising this important point. We have increased the text size in the revised manuscript.

- The data for younger (2.5 month old mice) is shown in Fig 7 C-D, while older mice (12 months old) are shown earlier in Fig 7 A-B. The figure may flow better by presenting data chronologically, with younger mice first, followed by older, or

alternatively having 2.5 vs 12 month old t-SNE plots side-by-side, so the reader can more easily visually identify the age-associated increase in clusters reported.

We thank the reviewer for raising this important point. We have changed the figure order in the revised manuscript.

Supplementary Figures

General (Overall) Comments:

- Some figures lack sub-categories, making them difficult to interpret e.g. Figures S2-S3 lack 'A' and 'B' labelling, although this is mentioned in the figure legends.

We have looked at Figures S2-S3 carefully and believe that our submitted version should have an A and B.

- Figure S4 should ideally have the markers labelled within the images themselves, in a similar fashion to what was presented in Figure 9.

We thank the reviewer for raising this important point. We have added the marker labels within the images in the new manuscript.

Reviewer: 2

Comments to the Author

This study investigates the relationship between Aβ oligomers, plaques, glial activation, and neurodegeneration markers in AppNL-G-F and Apphu mouse models of Alzheimer's disease (AD) using immunohistochemistry and imaging mass cytometry (IMC). The Apphu mice, which have abundant Aβ oligomers but lack plaques, show astrocyte activation without microglial activation or synaptic/neuronal pathology. In contrast, the AppNL-G-F mice exhibit high levels of Aβ oligomers and plaques, accompanied by significant astrocytic and microglial activation. The increase in Aβ oligomers over time in Apphu mice correlates more with astrocytic activation. Spatial analysis indicates a closer association between activated microglia and Aβ oligomers rather than plaques in AppNL-G-F mice.

The novelty of the work lies in using an Aβ oligomer-specific antibody to map and characterize the accumulation of Aβ oligomers in these relatively new models and identifying spatial relationships using IMC.

Overall, the findings are significant, but the article may benefit from addressing the following concerns:

Major concerns:

1-The article's main conclusion is somewhat ambiguous. The abstract mentions the dependency of glial cell activation on the aggregation state of Aβ, supported only by correlations drawn from immunohistochemistry findings across different models. However, spatial analysis indicates that in AppNL-G-F mice, microglia (and not astroglia) are more associated with oligomers. The authors might consider refining and reflecting their conclusion in the abstract and the title for clarity and accuracy.

We thank the reviewer for raising this important point. We have added the following line in the abstract:

"Although the glial and neuronal pathology is more strongly associated with Aβ oligomers than Aβ plaques, Aβ plaques also seem to play a role."

2- The results solely depend on the assumption that NAB61 identifies oligomers. While this was shown in previous articles using this particular antibody, it is known that there are many different kinds of Aβ aggregation intermediates. The results could be antibody-dependent if the antibody is selective to one specific intermediate. The authors discussed this limitation, but the study findings will be stronger if any of the experimental findings can be replicated using a different oligomeric Aβ antibody.

We apologize for this limitation. We explored use of A11 (Abcam, ab126892) but were not able to obtain strong immunostaining with our samples. By contrast, we found the NAB61 antibody provided reliable staining and recognized the face validity conferred by results showing that intravenous treatment with the antibody modified pathology progression in the mouse (PMID: 20205640). Dr Virginia Lee's lab in University of Pennsylvania also showed neurodegenerative changes related to the target Aβ species.

3- there needs to be a more comprehensive discussion regarding the absence of a correlation between astrocytic markers and Aβ oligomers in the AppNL-G-F model.

We apologize for not making this clear. Although the correlation plot did not show significant result, we found correlations between astrocytic markers and Aβ oligomers by the Sholl analysis. Generally, in 12-month $App^{NL\text{-}G\text{-}F}$ mice, microglia were mostly proximal to Aβ oligomers and plaques, whereas astrocytes were associated with amyloid species in a more scattered pattern (Fig. 6CDE). We have now added this information in the discussion.

Minor Comments:
In the abstract, consider replacing "expression" of Aβ oligomers with "levels." Also, the term "Human Aβ proteins" might be confusing, and an alternative like "Aβ

aggregates" might convey the concept more clearly.

Oligomers and plaque are formed and not expressed.
Similarly, the last sentence in the abstract is not entirely accurate. "..depends on nature and aggregation state of Aβ oligomeric species expressed". Aβ peptide aggregation states include dimers, Aβ oligomers, Aβ protofibrils, and Aβ plaque. So this sentence can be replaced with "depends on nature and aggregation state of Aβ peptides."

We thank the reviewer for raising this important point. We have now changed the phrase in the new manuscript.

Please add appropriate labels (6C3 or NAB61) to the figures in panels A and B of Figure 2.

We thank the reviewer for raising this important point. We have now added the marker labels in the figure.

For Figure 2, panel B, clarify whether the statistics were conducted using the number of animals or images/experiments.

We thank the reviewer for raising this important point. We have shown the datapoints in the figures in the new manuscript, and each datapoints is a different animal.

Throughout the manuscript, it would be beneficial if the figures displayed the number of animals to illustrate variability between subjects better.

We thank the reviewer for raising this important point. We have represented all data points in the figures in the new manuscript.

The authors should focus on discussing the significance and limitations of the main conclusions.

We thank the reviewer for raising this important point. We have included the following lines in the discussion:

"Future work may benefit from more detailed measures over the lifespan with App^{hu} mice."

"Aβ oligomers have been attracting our attention due to the neurotoxic properties, and our study is a novel and timely contribution to the field to claim the neurotoxicity on the basis of aggregation state."

Comments from the Scientific Editor:

-Please supply a graphical abstract. A graphical abstract is a visual representation of the central finding or methodology of the paper. It will be displayed in the table of contents. Authors should strive to make the graphical abstract informative, interesting, visually appealing, and straightforward (no caption needed, no abbreviations except gene names). The technical requirements for the graphical abstracts are:
- Size: 120 x 120 mm (300-600dpi) or 3000 x 3000 pixels
- Font: Arial 12-16 pt
- File type: tiff or PDF
- Aspect ratio: 1:1 preferred

-Please add an Abbreviated Summary (up to 50 words) of your paper that captures the main purpose and conclusions of the work. The summary should state the main results written in the third person ('[1st author surname] et al. report that ...') and conclusion. This summary will be used in the contents list online and should not contain any abbreviations other than those on the accepted abbreviations list and accepted gene/protein names, making it accessible to the non-specialist. Please upload it as a Word document. Allowed abbreviations: AIDS; ANOVA; AMPA; ATP; A,T,C,G; CNS; CSF; CT; DNA; ECG; EEG; EMG; GABA; HIV; MRI; NMDA; PET; PCR; RNA.

- Please include the appropriate checklist to comply with our ethical standards (see Reporting Guideline section within 'Instructions to authors').

We thank the editor for the comments. The graphical abstract, abbreviated summary and checklist have all been submitted with the revised manuscript.

-Please include a paragraph about statistical analysis in the 'Materials and Methods' section.

Our submitted manuscript included a sub-section named 'Statistical Analysis' in the 'Materials and Methods' section. We hope that this provides adequate detail, but, if not, can augment it.

-for each statistical analysis in a figure, please report in the figure legend, where missing the test used and relevant test statistic values alongside p values (for example F or t values for ANOVAs and t-tests). Also, please, provide details of the experimental unit for each group analysed in these plots, and if the number is <10 show the single data-points. Please note: We do not accept analyses where individual measurements are the experimental unit when there is likely to be biological variability between animals, culture replicates, etc. For this type of data, we require either that a mean or median is taken per animal/culture replicate followed by statistical analyses, or that more sophisticated analyses are performed

such as mixed effects modelling which can account for random effects.

We thank the editor for raising this important point. We have reported all F values in the figure legend alongside p values and shown datapoints in all plots. Each datapoint is one animal, with values representing the means from measures in three brain sections (replicates).
