# Supplementary material for: Associations of amyloid-β oligomers and plaques with neuropathology in the AppNL-G-F mouse
Source: Brain Commun. 2024 Jun 25;6(4):fcae218. doi: 10.1093/braincomms/fcae218 (PMC11258573; doi:10.1093/braincomms/fcae218)
Supplement: fcae218_Supplementary_Data [file Revision_1_ Response_to_reviewers_comments.pdf › Revision_1_ Response_to_reviewers_comments.pdf]

**Responses to reviewers:** Associations of Amyloid-β Oligomers and Plaques with Neuropathology in the *App*$^{NL-G-F}$ Mouse

We are grateful to the reviewers and have responded to points raised as below.

**Reviewer: 1**

Comments to the Author

I thank the authors for their response to my previous comments and acknowledge that a number of changes have been made that have improved the manuscript. I still however have some concerns that I feel could be addressed:

Methods

- Can the authors provide supplemental data of the sex comparison that was carried out for amyloid, glial and neuronal markers? It would be ideal to see empirical evidence that the variables tested in this study indeed demonstrated no difference. Although I still have some concerns with excluding female mice from the study, I think evidence supporting this decision would be beneficial.

We thank the reviewer for raising this important point. We have now added a supplementary figure, and included the following line in the methods:

"Sex comparison was conducted with pathology, and no consistent difference has been shown (Supplementary Fig. 1)."

Figure 1A-B: I'm still not confident that the images are entirely white balanced as some appear to have a different hue from others.

We apologize for the inconsistent white balance. We have adjusted the figure by retaking some images.

- Figure 2: The authors mention that they performed 3 technical replicates for quantifying synapse optical density. Could they clarify what the 6 datapoints in B-E represent? I assume that the 3 datapoints in F and G represent these replicates?

We are sorry if this was unclear. We studied 6 mice in each genotype group as indicated by the "n=6" in figure legends. Each data point represents the mean of 3 technical replicates. This now is clarified in the caption.

- Figure 2: Synaptophysin represents the gold standard for measuring pre-synaptic density. Has SV2A been compared against this? If not, could the authors provide any supporting evidence for the use of SV2A as a presynaptic marker here?

We thank the reviewer for raising this important point. Recent work has argued that synaptic vesicle glycoprotein 2A (SV2A) is expressed in all synaptic terminals (Rossi et al, 2022). Finnema et al. (2016) previously validate SV2A is an alternative presynaptic density marker to synaptophysin by showing a linear correlation between the two markers.

References:
Finnema S J, Nabulsi N B, Eid T, et al. Imaging synaptic density in the living human brain. Science translational medicine, 2016, 8(348): 348ra96-348ra96.

Rossi R, Arjmand S, Bærentzen S L, et al. Synaptic vesicle glycoprotein 2A: features and functions. Frontiers in Neuroscience, 2022, 16: 864514.

- Figure 2A: The representative brain regions are not consistent here. Although the authors state that images from different regions were chosen on the basis of better contrast, it would still be ideal to include a second panel just showing frontal cortex (SV2A, PSD-95 and NeuN) and include NeuN from the hippocampus.

We thank the reviewer for raising this important point. We have included a second panel to show representative images in two different brain regions in the revised manuscript.

- Figure 5A: Although CD163 shows little expression in WT mice, it would still be useful to have a representative image side-by-side to allow for visual comparison.

We thank the reviewer for raising this important point. We have put side-by-side images in Supplementary Figure 7 for the four glial markers, including CD163.

**Reviewer: 2**
Comments to the Author
The authors have addressed the majority of my concerns, but please address the two minor comments below:

In the newly added sentence in the abstract
"Although the glial and neuronal pathology is more strongly associated with Aβ oligomers than Aβ plaques, Aβ plaques also seem to play a role.

Please be more specific and describe Aβ plaques' role in a few words.

We thank the reviewer for raising this important point. We have changed the sentence to be more specific on the role of Aβ plaques in microglia activation:

“Astrocyte activation and neuronal pathology appeared to be more strongly associated with Aβ oligomers than Aβ plaques, although Aβ plaques were associated with microglia activation.”

2- Please modify the title to highlight that this study mainly focused on the differential effects of oligomers and amyloid plaque. The current title needs to be more specific.

We thank the reviewer for raising this important point. We have modified the title to be the following line:

“Associations of Amyloid-β Oligomers and Plaques with Neuropathology in the $App^{NL\text{-}G\text{-}F}$ Mouse.”

Comments from the Scientific Editor:

-Please include the page numbers corresponding to the items listed in the reporting guideline checklist.

We thank the editor for raising this important point. The page numbers are included in the revised manuscript.

- For all the bar charts in figures, please, specify in the legend what each data point represents.

We thank the editor for raising this important point. We have now included the following line in all legends related to bar charts:

“Each data point represents the mean of three technical replicates in one mouse.”
