# Supplementary material for: Associations of amyloid-β oligomers and plaques with neuropathology in the AppNL-G-F mouse
Source: Brain Commun. 2024 Jun 25;6(4):fcae218. doi: 10.1093/braincomms/fcae218 (PMC11258573; doi:10.1093/braincomms/fcae218)
Supplement: fcae218_Supplementary_Data [file Revision_1_decision_letter.pdf › Revision_1_decision_letter.pdf]

From: brcoms.editorialoffice@oup.com
To:
CC:
BCC:
Subject: Brain Communications - Decision on Manuscript ID BRAINCOM-2023-360.R1
Body: 24-Jan-2024

Dear Paul,

Thank you for your recent submission BRAINCOM-2023-360.R1
entitled "Neuropathology Associated with Amyloid-β Oligomers in the App Mouse" to Brain Communications.

Your manuscript has been reviewed, and while the reviewers had largely positive comments, they have some constructive critiques that I think you could address in a revision of the manuscript. Therefore, I invite you to respond to the reviewer(s)' comments and revise your manuscript. The reviewer(s)' comments are included at the bottom of this message.

In addition to the reviewers' comments, please also address our Scientific Editor's comments added below to comply with our requirements for presentation of scientific data at Brain Communications.

Please format your revised manuscript according to our Instructions to Authors
https://academic.oup.com/braincomms/pages/General_Instructions
A summary of key points is attached to help with this. Please note that there can be substantial delays to publication if you don't resubmit in line with our requirements.

To revise your manuscript, click the link below or log into
https://mc.manuscriptcentral.com/braincom and enter your Author Centre, where you will find your manuscript title listed under "Manuscripts with Decisions." Under "Actions," click on "Create a Revision." Your manuscript number will be appended to denote a new revision.

*** PLEASE NOTE: This is a two-step process. After clicking on the link, you will be directed to a webpage to confirm. ***

https://mc.manuscriptcentral.com/braincom?URL_MASK=074e064644994b3d86f99a2459885bc6

Please upload a revised manuscript with changes tracked using track changes or in coloured text.

When submitting your revised manuscript, you will be asked to respond to the reviewer(s) comments. Please be as specific as possible in your response to all of the comments.

The editorial team at Brain Communications also aims to increase transparency in scientific

publishing and to demonstrate the value that peer review brings to the scientific process. Please therefore consider whether you are willing to have reviewer comments on your paper and your response published as supplementary data with the paper if it is accepted. This is entirely optional and will only be done if both the authors and reviewers agree. When submitting your revision you will have the opportunity to update the answer you entered previously.

IMPORTANT: Your original files are available to you when you upload your revised manuscript. Please delete any redundant files before completing the submission.

Because we are trying to facilitate timely publication of manuscripts submitted to Brain Communications, we would appreciate your revised manuscript as soon as possible. If it is not possible for you to resubmit within 3 months, the option to submit a revision will expire, and we would then consider your revised paper as a new submission. However, if you need a little more time, please get in touch and we can extend this deadline for you.

Once again, thank you for submitting your manuscript to Brain Communications and I look forward to receiving your revision.

Best wishes

Professor Tara Spires-Jones
Founding Editor, Brain Communications

Reviewer(s)' Comments to Author:

Reviewer: 2

Comments to the Author
The authors have addressed the majority of my concerns, but please address the two minor comments below:

In the newly added sentence in the abstract
"Although the glial and neuronal pathology is more strongly associated with Aβ oligomers than Aβ plaques, Aβ plaques also seem to play a role.

Please be more specific and describe Aβ plaques' role in a few words.

2- Please modify the title to highlight that this study mainly focused on the differential effects of oligomers and amyloid plaque. The current title needs to be more specific.

Reviewer: 1

Comments to the Author
I thank the authors for their response to my previous comments and acknowledge that a number of changes have been made that have improved the manuscript. I still however have some concerns that I feel could be addressed:

Methods

- Can the authors provide supplemental data of the sex comparison that was carried out for amyloid, glial and neuronal markers? It would be ideal to see empirical evidence that the variables tested in this study indeed demonstrated no difference. Although I still have some concerns with excluding female mice from the study, I think evidence supporting this decision would be beneficial.

- Figure 1A-B: I'm still not confident that the images are entirely white balanced as some appear to have a different hue from others.

- Figure 2: The authors mention that they performed 3 technical replicates for quantifying synapse optical density. Could they clarify what the 6 datapoints in B-E represent? I assume that the 3 datapoints in F and G represent these replicates?

- Figure 2: Synaptophysin represents the gold standard for measuring pre-synaptic density. Has SV2A been compared against this? If not, could the authors provide any supporting evidence for the use of SV2A as a presynaptic marker here?

- Figure 2A: The representative brain regions are not consistent here. Although the authors state that images from different regions were chosen on the basis of better contrast, it would still be ideal to include a second panel just showing frontal cortex (SV2A, PSD-95 and NeuN) and include NeuN from the hippocampus.

- Figure 5A: Although CD163 shows little expression in WT mice, it would still be useful to have a representative image side-by-side to allow for visual comparison.

Comments from the Scientific Editor:

-Please include the page numbers corresponding to the items listed in the reporting guideline checklist.

- For all the bar charts in figures, please, specify in the legend what each data point represents.
